# Supplementary material for: Durvalumab and guadecitabine in advanced clear cell renal cell carcinoma: results from the phase Ib/II study BTCRC-GU16-043
Source: Nat Commun. 2024 Feb 1;15:972. doi: 10.1038/s41467-024-45216-z (PMC10834488; doi:10.1038/s41467-024-45216-z)
Supplement: Supplementary file 1 — Supplementary Information [file 41467_2024_45216_MOESM1_ESM.pdf]

Durvalumab and Guadecitabine in Advanced Clear Cell Renal Cell Carcinoma: Results from the Phase Ib/II Study, BTCRC-GU16-043

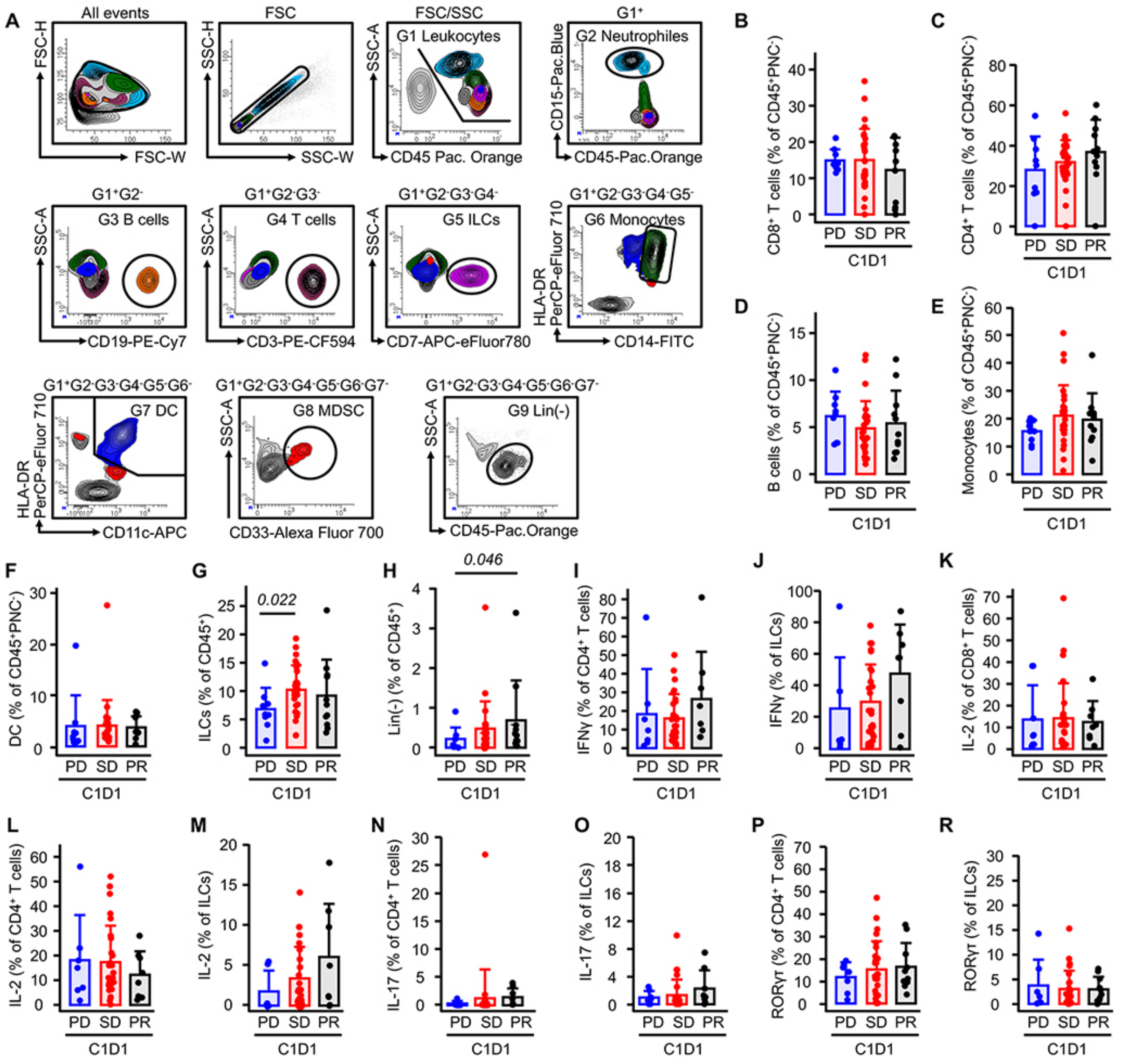

Supplementary Figure 1. The relationship between immune cell phenotype and response.

(A) Gating strategy of flow cytometry analysis of peripheral blood mononuclear cells.
(B - H) Response assessment was performed using RECIST1.1 and allowed patients to be grouped as those with progressive disease (PD n=9), stable disease (SD n=28), and partial or complete response (PR n=11). The distribution of basic immune cell subsets (B - H), cytokine production (I-O) and RORyt expression (P-R) by lymphocytes and ILC cells in patients with different response rates. Results are expressed as mean + SEM.

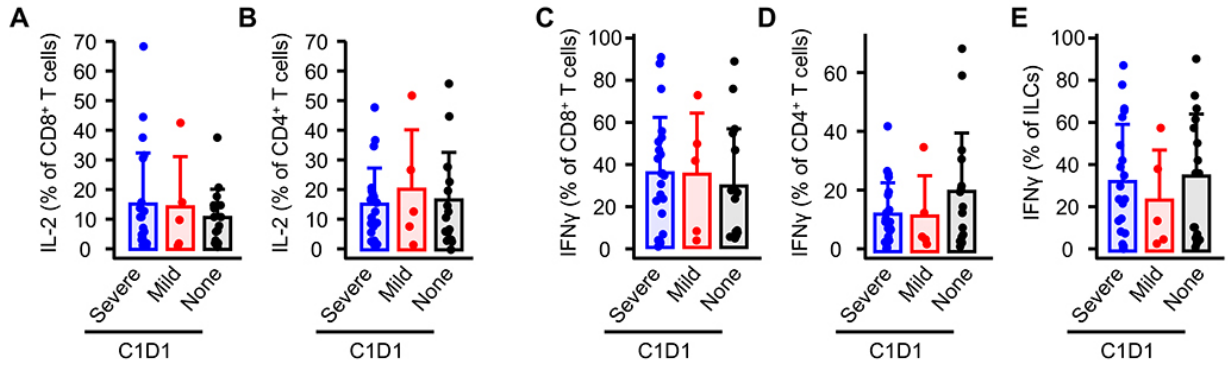

Supplementary Figure 2. The relationship between immune cell phenotype and immune-mediated toxicity.

Mononuclear cells isolated from peripheral blood collected before treatment (C1D1) were analyzed by flow cytometry. Patients were categorized based on their CTCAE grade of immune-mediated toxicity into Grade 1-2 (mild n=5), Grade 3-5 (severe n=24) and no immune-mediated toxicity (none) (n=19). Results are expressed as mean + SEM, one-sided paired t-test.

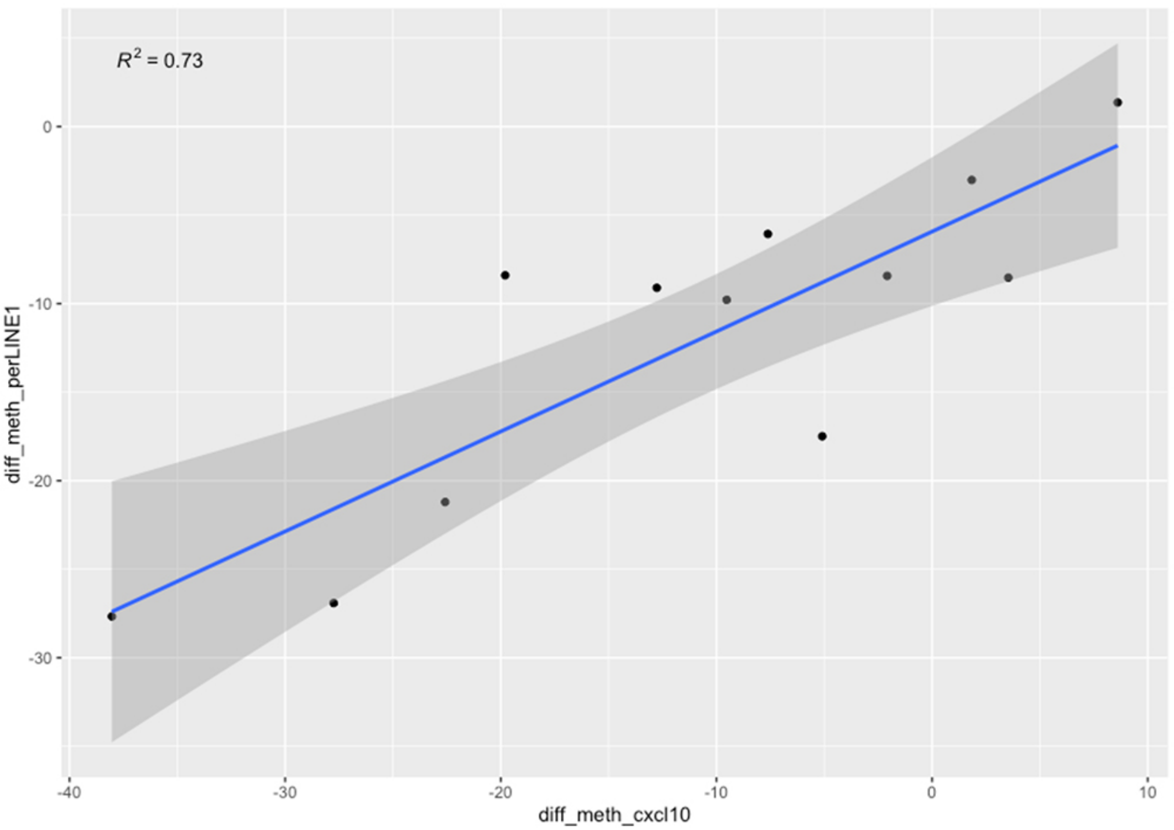

Supplementary Figure 3: Correlation of change in methylation between LINE-1 and CXCL10 regions for the selected samples (n= 12) with more or less than 15% change in plasma CXCL10.

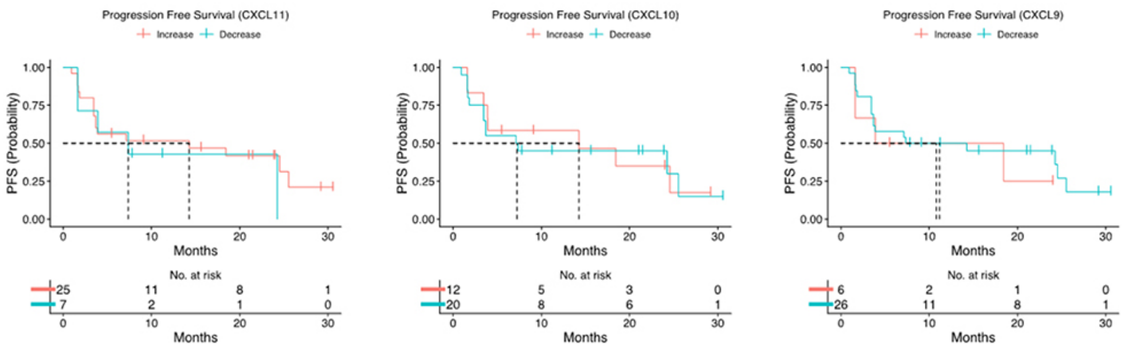

Progression-free survival in correlation to CXCL 9, 10, 11 changes

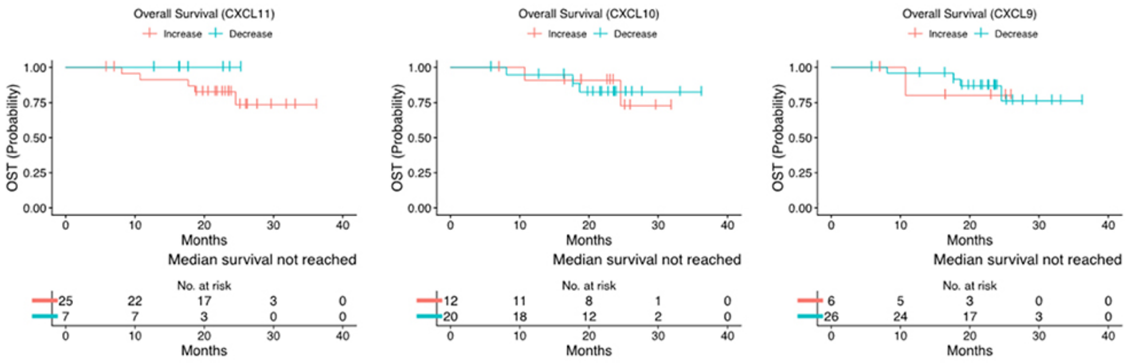

Overall survival in correlation to CXCL 9, 10, 11 changes

Supplementary Figure 4. The association between chemokines' (CXCL9, 10 and 11) serum levels and patient's clinical outcome as measure by progression free survival (PFS) and overall survival (OS).

Supplementary Table 1: Efficacy.

Characteristic	C1 (N=36)	C2 (N=15)
**Best RECIST Response**		
CR	1 (2.7%)	0 (0%)
PR	7(20%)	1 (7%)
SD	16 (44%)	9 (60%)
PD	12 (33%)	5 (33%)
**Best irRC**		
irCR	1 (2.7%)	0 (0%)
irPR	7 (20%)	1 (7%)
irSD	14 (39%)	9 (60%)
irPD	14 (39%)	5 (33%)

RECIST: Response Evaluation Criteria in Solid Tumors; irRC: immune-related Response Criteria

Supplementary Table 2: Increase and decrease denote positive and negative differences between C2D8 and C1D1 in log-scale.

Characteristic	CXCL 11		CXCL 10		CXCL 9	
	Increase	Decrease	Increase	Decrease	Increase	Decrease
**Best RECIST Response**						
CR	0	1	0	1	0	1
PD	2	5	2	4	2	4
PR	2	3	3	2	1	4
SD	9	10	8	12	4	16
**Total**	**13**	**19**	**13**	**19**	**7**	**25**

CXCL11, CXCL10, and CXCL9 were analyzed by Luminex in serum collected before (C1D1) and at 5 weeks (C2D8) of treatment. Response assessment was performed using RECIST1.1 and allowed patients to be grouped as those with progressive disease (PD), stable disease (SD), and partial or complete response (PR/ CR)

SD, PR and CR are grouped together vs PD.

Based on the binomial proportion test (p-value <0.1), increased CXCL11 patient IDs are related to Best RECIST response of (SD, PR, and CR) group, (P= 0.000208).

Based on the binomial proportion test (p-value <0.1), increased CXCL10 patient IDs are related to Best RECIST response of (SD, PR, and CR) group, (P= 0.000208).

Based on the binomial proportion test (p-value <0.1), increased CXCL9 patient IDs are related to Best RECIST response of (SD, PR, and CR) group, (P= 0.054).

Supplementary Table 3

Participating Institutions Institutional review boards (IRBs)
University of Iowa Holden Comprehensive Cancer Center
Rutgers Cancer Institute of New Jersey
Penn State Cancer Institute
University of Illinois at Chicago
University of Michigan

Supplementary Table 4: Antibodies used for flow cytometry.

Target	Fluorchrome	Clone	Cat. number	Company
CD4	Pacyfic Blue	RM4-5	MCD0428	ThermoFisher
CD3	PE-CF594	UCHT1	562280	BD Biosciences
CD3	PE-Cyanine7	UCHT1	25-0038-42	ThermoFisher
CD3	Pacyfic Orange	UCHT1	CD0330	ThermoFisher
CD7	APC-eFluor® 780	124-1D1	47-0079-42	ThermoFisher
CD8	Alexa Fluor 700	RPA-T8	557945	BD Biosciences
CD8	PerCP-Cyanine5.5	RPA-T8	560662	BD Biosciences
CD11c	APC	B-ly6	559877	BD Biosciences
CD14	FITC	M5E2	555397	BD Biosciences
CD15	eFluor 450	MMA	48-0158-42	ThermoFisher
CD19	PE-Cyanine7	SJ25C1	50-154-71	ThermoFisher
CD33	Alexa Fluor 700	WM-53	56-0338-42	ThermoFisher
CD45	Pacyfic Orange	HI30	MHCD4530	ThermoFisher
CD274	PE	MIH1	557924	BD Biosciences
CD279	PE	MIH4	558694	BD Biosciences
HLA-DR	PerCP-eFluor 710	L243	46-9952-42	ThermoFisher
FoxP3	eFluor 450	236A/E7	48-4777-42	ThermoFisher
GATA3	PE-CF594	L50-823	563510	BD Biosciences
RORyt	PE	Q21-559	563081	BD Biosciences
T-bet	PerCP-Cyanine5.5	4B10	45-5825-82	ThermoFisher
Ki-67	Alexa Fluor 488	SolA15	53-5698-82	ThermoFisher
Gran. B	PE-CF594	GB11	562462	BD Biosciences
IL-2	PE	MQ1-17H12	554566	BD Biosciences
IL-17	Alexa Fluor 700	N49-653	560613	BD Biosciences
IL-22	APC	4F1	generated in lab	
IFNy	PE-Cyanine7	B27	557643	BD Biosciences
TNFa	FITC	MAb11	552889	BD Biosciences

Single Arm Phase Ib/II Study of Durvalumab and Guadecitabine in Advanced Kidney Cancer: Big Ten Cancer Research Consortium BTCRC-GU16-043

Sponsor Investigator
Ajjai Alva, MD
University of Michigan
ajjai@med.umich.edu

Co-Investigators
Weiping Zou, MD, Ph.D.
University of Michigan

Statistician
Ryan Ross, MS
University of Michigan

Trial Management Provided by
Big Ten CRC Administrative Headquarters at Hoosier Cancer Research Network, Inc.
7676 Interactive Way, Suite 120
Indianapolis, IN 46278

Trial Supported by
AstraZeneca Pharmaceuticals, LP
(ESR-16-12275)

Astex Pharmaceuticals, Inc.
(EP23)

Investigational New Drug (IND) Number:
136079

Initial Protocol Version Date: 12MAY2017

Protocol Amendment Version Date:
13AUG2017
10APR2018
12DEC2018
13SEP2019 (FDA only)
06FEB2020
14DEC2020 (current)

PROTOCOL SIGNATURE PAGE

Single arm Phase Ib/II Study of Durvalumab and Guadecitabine in Advanced Kidney Cancer:
Big Ten Cancer Research Consortium BTCRC-GU16-043

VERSION DATE: 14DEC2020

I confirm I have read this protocol, I understand it, and I will work according to this protocol and to the ethical principles stated in the latest version of the Declaration of Helsinki, the applicable guidelines for good clinical practices, whichever provides the greater protection of the individual. I will accept the monitor's overseeing of the study. I will promptly submit the protocol to applicable ethical review board(s).

________________________________ ______________________
Signature of Site Investigator Date

____
Site Investigator Name (printed)

____
Site Investigator Title

____
Name of Facility

____
Location of Facility (City and State)

PLEASE EMAIL COMPLETED FORM TO
BIG TEN CRC ADMINISTRATIVE HEADQUARTERS

SYNOPSIS

**TITLE**	Single Arm Phase Ib/II study of Durvalumab and Guadecitabine in Advanced Kidney Cancer
**PHASE**	Phase Ib/II
**OBJECTIVES**	Phase Ib<br>Primary Objective<br>1. To estimate the safety and toxicities of durvalumab in combination with guadecitabine in metastatic clear cell renal cell carcinoma<br>Phase II<br>Primary Objective:<br>1. To assess the efficacy (primary endpoint: Objective Response Rate (CR + PR) as measured by RECIST 1.1) of durvalumab plus guadecitabine in patients with advanced RCC in Cohort 1 (patients with no prior anti-PD-1/PD-L1/CTLA4 therapy).<br>Secondary Objectives:<br>1. To assess the efficacy of durvalumab plus guadecitabine in patients with advanced RCC. Secondary endpoints include: the 2-year overall survival proportion, duration of response (DoR), 12-month progression-free survival (PFS), clinical benefit rate (proportion of patients with a best response of partial/complete response/stable disease for at least 6 months), complete response (CR) rate, objective response rate (CR + PR) as measured by the immune related response criteria (irRC) and the objective response rate (CR + PR) of patients treated with durvalumab and guadecitabine in cohort 2<br>• Phase II secondary endpoints will be evaluated separately for cohort 1 and 2 and will be based on RECIST 1.1 unless otherwise specified.<br>2. To evaluate the safety of guadecitabine in combination with durvalumab (secondary endpoint: toxicity by CTCAE ver 4 including events of special interest such as immune mediated toxicities) in advanced RCC (Cohorts 1 and 2).<br>Exploratory Objectives:<br>1. To correlate changes in CXCL9 and CXCL10 in tissue and serum as well as and LINE-1 methylation in blood with clinical response to the combination therapy<br>2. To correlate CD3+/CD8+ tumor infiltrating lymphocytes (TILs) and PD-L1 expression in baseline tumor tissue/TILs as well as immune cell subsets in the serum with clinical response to the combination therapy<br>3. To correlate tumor mutational burden and epigenetic changes with response to the combination therapy.<br>4. Analyze and correlate changes in methylation status and correlate tumor mutation profile in fresh tissue with response to the combination therapy
**STUDY DESIGN**	This is a single arm, multi-centre (via Big Ten Cancer Research Consortium) phase Ib/II study of patients treated with durvalumab 1500 mg

	IV q 4 weeks in combination with guadecitabine at the recommended phase 2 dose subcutaneously for 5 consecutive days. Eligible patients will have metastatic RCC with a clear cell component, ECOG performance status of 0-1, have received 0-1 prior therapy but no prior anti-PD-1/PD-L1/CTLA4 (Cohort 1, 36 subjects). Study treatment may continue until disease progression or unacceptable toxicity.<br><br>A smaller Cohort 2 (16 subjects) will comprise patients who have received up to 2 prior therapies including one of them necessarily an anti-PD-1/PD-L1 therapy but did not respond to the anti-PD-1/PD-L1 therapy.<br><br>We will start with a phase Ib study to estimate the safety and toxicity of the combination of durvalumab and guadecitabine.<br><br>Phase Ib Portion:<br>Patients from either cohort are eligible for enrollment in the phase 1b portion of the study and will be counted towards the efficacy analysis (in addition to the safety/toxicity analysis) of their respective phase II cohorts if treated at the eventual recommended phase II dose.<br><br>6 patients will be treated at dose level 0 and observed for toxicity. If 2 or fewer patients experience a dose limiting toxicity, then the study will continue to the phase II portion using dose level 0 as the treatment dose. If 3 or more patients have a dose limiting toxicity, then another 6 patients will be accrued at the lower dose (dose -1). If 2 or fewer patients have a dose limiting toxicity then we continue to phase II at this dose, otherwise the trial stops.<br><br>Dose Level 0: Patients will receive guadecitabine 60 mg/m^2 subcutaneously for 5 consecutive days starting day 1 of a 28-day cycle and durvalumab 1500mg IV on day 8.<br><br>Dose Level -1: Patients will receive guadecitabine 45 mg/m^2 subcutaneously for 5 consecutive days starting day 1 of a 28-day cycle and durvalumab 1500mg IV on day 8.
**KEY ELIGIBILITY CRITERIA**	1. Age ≥ 18years at the time of consent.<br>2. ECOG Performance Status 0-1 within 28 days prior to registration.<br>3. Histological diagnosis of clear cell renal cell carcinoma (pure or mixed) with radiologic or histologic evidence of metastatic disease<br>4. At least 1 lesion, not previously irradiated that can be accurately measured at baseline as ≥ 10 mm in the longest diameter (except lymph nodes which must have a short axis ≥ 15mm) with a computed tomography (CT) or magnetic resonance imaging (MRI) and that is suitable for accurate repeated measurements as per Response Evaluation Criteria in Solid Tumors, version 1.1 (RECIST 1.1) guidelines

5. Patients with suspected brain metastases at screening should have an MRI (preferred) or CT each preferably with IV contrast of the brain prior to study entry. Patients whose brain metastases have been treated may be considered if they have completed their treatment for brain metastasis at least 4 weeks prior to study registration provided they show radiographic stability (defined as 1 brain image, obtained after treatment to the brain metastases). In addition, any neurologic symptoms that developed either as a result of the brain metastases or their treatment must have resolved or be stable without the use of steroids for at least 14 days prior to the start of treatment.
6. For cohort 1, subjects may have received up to 1 and no more than 1 prior line of systemic therapy (not counting any neoadjuvant/adjuvant therapy) including anti-VEGF, VEGFR inhibitor, MET inhibitor or mTOR inhibitor for metastatic disease. They cannot have received any prior anti-PD-1/PD-L1/CTLA4 therapy.
7. For cohort 2, subjects may have received up to 2 prior systemic therapies which should include 1 (and only 1) prior anti-PD-1/PD-L1 therapy but did not have an objective response to the prior anti-PD-1/PD-L1 therapy. The treating investigator must document that the patient did not have an objective response to prior anti-PD-1/PD-L1 therapy. They may have received prior anti-CTLA4 therapy.
8. Subjects may not have had radiotherapy treatment to more than 30% of the bone marrow or with a wide field of radiation within 4 weeks of the first dose of study drug.
9. Prior cancer treatment must be completed at least 14 days prior to start of protocol specified therapy and the subject must have recovered from all reversible acute toxic effects of the regimen (other than alopecia) to ≤Grade 1 or baseline.
10. Demonstrate adequate organ function as defined in the table below; all screening labs to be obtained within 28 days prior to registration.

System	Laboratory Value
**Hematological**	
White blood cell (WBC)	≥ 3 K/mm^3
Absolute Neutrophil Count (ANC)	≥ 1.5 K/mm^3
Hemoglobin (Hgb)	≥ 9 g/dL
Platelets (Plt)	≥ 100,000/mm^3
**Renal**	
Calculated creatinine clearance	≥ 40 cc/min using the Cockcroft-Gault formula
**Hepatic**	
Bilirubin	≤ 1.5 × upper limit of normal (ULN)
Aspartate aminotransferase (AST)	≤ 2.5 × ULN
Alanine aminotransferase (ALT)	≤ 2.5 × ULN

**STATISTICAL CONSIDERATIONS**	Phase Ib portion:<br>6 patients will be treated at dose level 0 and observed for toxicity. If 2 or fewer patients experience a dose limiting toxicity, then the study will continue to the phase II portion using dose level 0 as the treatment dose. If 3 or more patients have a dose limiting toxicity, then another 6 patients will be accrued at the lower dose (dose -1). If 2 or fewer patients have a dose limiting toxicity then we continue to phase II at this dose, otherwise the trial stops.<br><br>Dose Level 0: Patients will receive guadecitabine 60 mg/m^2 subcutaneously for 5 consecutive days starting day 1 of a 28-day cycle and durvalumab 1500mg IV on day 8.<br><br>Dose Level -1: Patients will receive guadecitabine 45 mg/m^2 subcutaneously for 5 consecutive days starting day 1 of a 28-day cycle and durvalumab 1500mg IV on day 8.<br><br>Patients from either cohort are eligible for enrollment in the phase 1b portion of the study and will be counted towards the efficacy analysis (in addition to the safety/toxicity analysis) of their respective phase II cohorts if treated at the eventual recommended phase II dose.<br><br>Phase II portion:<br>Based on the toxicities observed in the Phase Ib subjects and new data from other ongoing clinical trials with guadecitabine, the recommended phase II dose of guadecitabine will be 45 mg/m^2. Although formal DLT definitions were not met in the phase Ib portion, three of five Phase Ib subjects who received dose of 60 mg/m^2/d guadecitabine were found to have significant neutropenia (grade 3 or 4) on Cycle 2 Day 1 requiring delay/skipping of Cycle 2 guadecitabine. Data from the other guadecitabine studies in combination with durvalumab (e.g. Guadecitabine and Durvalumab in Treating Patients With Advanced Liver, Pancreatic, Bile Duct, or Gallbladder Cancer [NCT03257761]; SGI-110 Plus Durvalumab/Tremelimumab in SCLC [NCT03085849]) support a 45 mg/m^2 dose. Therefore, the Phase II starting dose of guadecitabine will be 45 mg/m^2 subcutaneous daily on days 1-5 with a fixed dose of durvalumab at 1500 mg IV on day 8.<br><br>Primary endpoint for the phase II portion is Objective Response Rate (CR+PR = ORR) or proportion as measured by RECIST 1.1 in advanced kidney cancer patients treated with durvalumab and guadecitabine in Cohort 1.<br><br>Assuming an ORR of 25% in patients treated with durvalumab alone (akin to published data from another anti-PD-1 therapy, nivolumab), a sample size of **36 patients in cohort 1** can detect an improvement to 45% ORR in patients treated with the combination of durvalumab + guadecitabine with

	80% power assuming a one-sided 4.6% type I error with an exact binomial test. Any patient in the phase Ib portion treated at the eventual recommended phase II dose of both drugs will be included in the phase II efficacy analysis.<br><br>Additionally, 16 patients will be enrolled on to cohort 2 (patients who did not respond to prior anti-PD-1/PD-L1 therapy) with 80% power to detect an ORR of 25% compared to an expected ORR of 5% or less with a type I error of 4.3% with an exact binomial test.<br><br><u>Primary Endpoint Analysis:</u><br>The primary endpoint analysis will use a binomial exact test to compare the ORR (as measured by RECIST 1.1) proportion in patients treated with durvalumab + guadecitabine in Cohort 1 compared to an expected proportion of 25% with durvalumab alone. The proportion of patients who have disease response of CR or PR will be reported with the 90% exact binomial confidence interval. A similar analysis will be completed in cohort 2 comparing cohort 2 to an expected proportion of 5%.<br><br><u>Secondary Endpoints Analysis:</u><br>Overall survival, progression-free survival and duration of response will be assessed using Kaplan-Meier estimates including the 95% confidence band separately for cohort 1 and for cohort 2. Overall survival proportions at 2 years will be reported with 95% confidence intervals from the Kaplan-Meier estimates. Complete response rate, clinical benefit rate (CR+PR+SD for 6 months) objective response rate as measured by immune related response criteria, and objective response rate of cohort 2 as measured by RECIST 1.1 will be reported as binomial proportions and corresponding 95% binomial confidence intervals separately for cohort 1 and cohort 2.<br><br>Analysis of toxicity as measured using CTCAE v4 will be described by body system including maximum grade and attribution as proportions of all (cohort 1 and cohort 2) treated patients. Dose reductions and holds will describe tolerability of therapy.
**TOTAL NUMBER OF SUBJECTS**	N = 58 patients in both phases<br>• 36 in cohort 1 and 16 in cohort 2<br>• 6 in Phase Ib; 52 in phase II (Any phase Ib patient treated at the eventual recommended phase II dose will be included in the phase II efficacy analysis.)
**ESTIMATED ENROLLMENT PERIOD**	12 months
**ESTIMATED STUDY DURATION**	36 months

TABLE OF CONTENTS

Schema 10
1. Background and Rationale 11
1.1 Disease Background 11
1.2 Current Standard of Care 11
1.3 Guadecitabine and Durvalumab 11
1.4 Rationale 14
2. Study Objectives and Endpoints 14
2.1 Objectives 14
2.2 Endpoints 15
3. Eligibility Criteria 16
3.1 Inclusion Criteria 16
3.2 Exclusion Criteria 17
4. Subject Registration 19
5. Treatment Plan 20
5.1 Study Design 20
5.2 Number of Subjects 20
5.3 Phase Ib Treatment Plan 20
5.4 Dose-Limiting Toxicity (DLT): 21
5.5 Treatment Plan for the Phase II Portion 22
5.6 Pre-medication and Hydration 23
5.7 Concomitant Medications 23
5.8 Contraception 25
5.9 Blood donation 26
6. Toxicities and Dose Delays/Dose Modifications 26
6.1 Dose Delays/Dose Modifications 26
6.2 Dose Levels/ Dose Reductions 27
6.3 Protocol Therapy Discontinuation 28
6.4 Criteria for Continuing Study Treatment Beyond 1st Progression: 28
6.5 Protocol Discontinuation 29
6.6 Duration of Protocol Therapy 29
7. Study Calendar & Evaluations 30
7.1 Screening Evaluations 32
7.2 On Treatment Evaluations 32
7.3 Safety Follow-up Evaluations 34
7.4 Long Term Follow-up Evaluations 34
8. Biospecimen Studies and Procedures 34
8.1 Source and Timing of Biospecimen Collections 35
8.2 Storage of Biospecimens 35
8.3 Banking of Leftover Biospecimens 36
8.4 Samples for future studies 36
8.5 Confidentiality of Biospecimens 36
9. Criteria for Disease Evaluation 36
9.1 Measurable Disease 36
9.2 Non-measurable Lesions 37

9.3 Target Lesions 37
9.4 Non-target Lesions 37
9.5 Evaluation of Target Lesions 37
9.6 Evaluation of Non-Target Lesions 38
9.7 Evaluation of Best Overall Response 38
9.8 Definitions for Response Evaluation – RECIST 1.1 39
10. Drug Information 43
10.1 Durvalumab 43
10.2 Guadecitabine 45
11. Adverse Events 46
11.1 Definitions 46
11.2 Reporting 51
12. Statistical Methods 56
12.1 Study Design 56
12.2 Endpoints 56
12.3 Sample Size and Accrual 57
12.4 Analysis Datasets 57
12.5 Evaluable for Safety 57
12.6 Evaluable for Efficacy 58
12.7 Data Analysis Plans 58
13. Trial Management 59
13.1 Data and Safety Monitoring Plan (DSMP) 59
13.2 University of Michigan's Data Safety Monitoring Committee 59
13.3 Data Quality Oversight Activities 60
13.4 Compliance with Trial Registration and Results Posting Requirements 60
14. Data Handling and Record Keeping 60
14.1 Data Management 60
14.2 Case Report Forms and Submission 61
14.3 Record Retention 61
14.4 Confidentiality 61
15. Ethics 61
15.1 Institutional Review Board (IRB) Approval 61
15.2 Ethical Conduct of the Study 62
15.3 Informed Consent Process 62
16. References 63
17. Appendix I: Durvaluamb Toxicity Management Guidelines 64

SCHEMA

**Cohorts** (total of 52 subjects)<br>Both cohorts will enroll simultaneously throughout Phase I and Phase II	
Cohort 1 (36 subjects)<br>• up to 1 prior therapy<br>• no prior anti-PD-1/ PD-L1/ CTLA4 allowed	Cohort 2 (16 subjects)<br>• up to 2 prior therapies for advanced kidney cancer.<br>• One of these therapies must be a prior anti-PD-1/PD-L1 drug to which they did not respond.

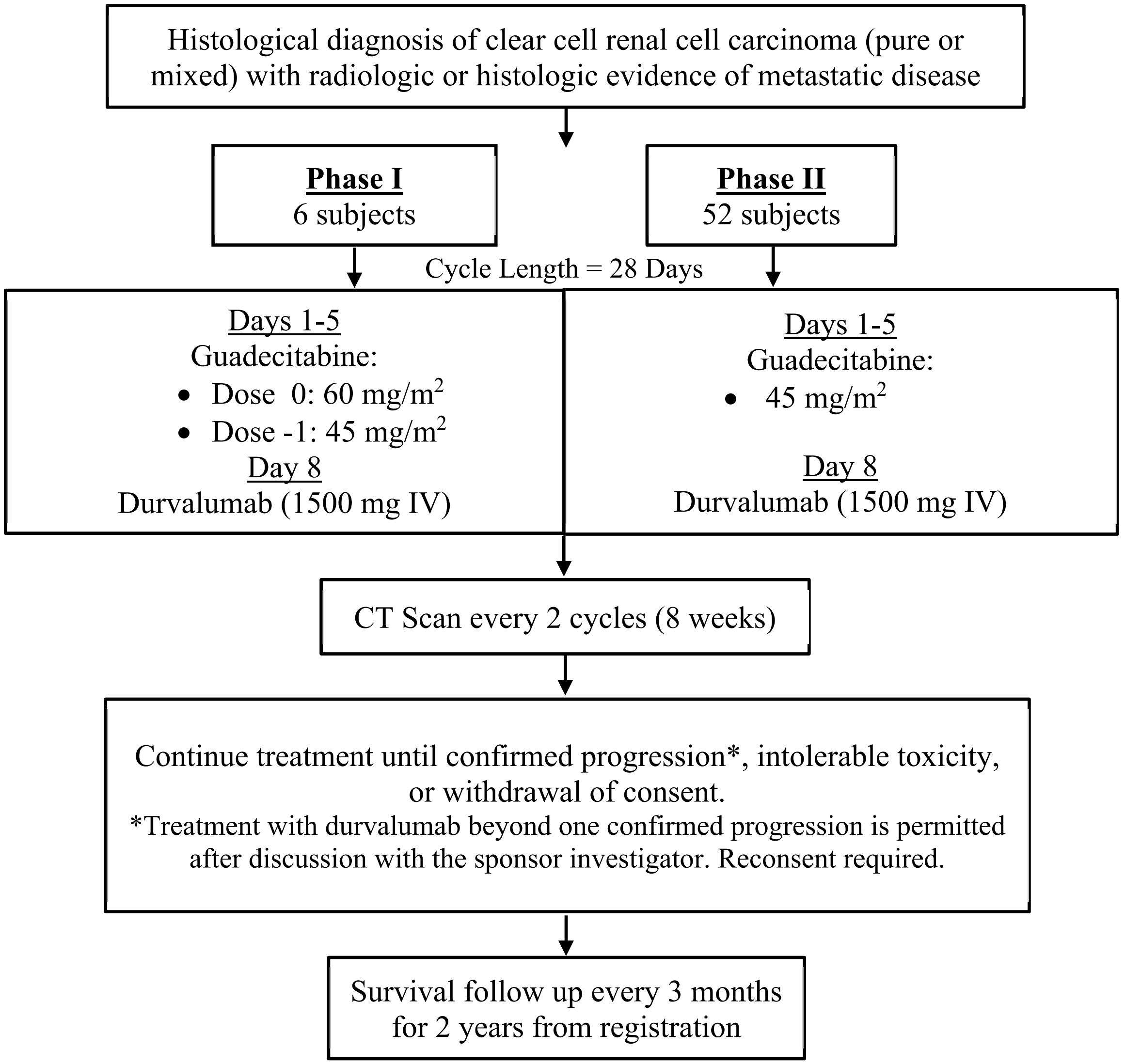

1. BACKGROUND AND RATIONALE

1.1 Disease Background

An estimated 62,700 cases of kidney cancer (RCC) were diagnosed in the U.S. in 2016 with an estimated 14,240 deaths (SEER). There has been a steady 2-4% per year increase in the incidence of RCC since 1975 that is not explained by increased and improved imaging studies. Clear cell cancers are the most common variant of kidney cancers comprising up to 80% of RCC. The five-year survival rate for patients with renal cell carcinoma is 70%, however, this includes the majority of subjects with localized disease whose five-year survival is 91%. At the time of diagnosis approximately 30% of RCC subjects have metastatic disease and another 30% of subjects recur. Unfortunately, the five-year survival for metastatic disease is less than 10%. Hence, there remains an urgent need for improvement in the therapeutic management of metastatic clear cell RCC (mRCC).

1.2 Current Standard of Care

The current front line standard of care treatment for metastatic kidney cancer is molecularly targeted therapy, typically with an inhibitor targeting the vascular endothelial growth factor (VEGF) or its receptor (VEGFR). Unfortunately, the median progression free survival on such first line therapy is only 7-9 months [1] and over the course of their treatment, most patients undergo multiple lines of therapy. There are now several drugs approved for the second line treatment of metastatic renal cell carcinoma, including nivolumab, an anti-PD-1 monoclonal antibody. In a phase 3 clinical trial, nivolumab was compared to everolimus for second line treatment of metastatic kidney cancer. Nivolumab led to a significantly higher response rate (25% vs 5%) as well as a significantly prolonged overall survival (25 months vs. 19.6 months) [2]. Response to immunotherapy is often durable unlike response to VEGFR inhibitors. Nivolumab has become standard second line therapy in patients with metastatic renal cell carcinoma. While an improvement over prior therapy, only a minority of patients respond to treatment with anti-PD-1 monotherapy.

1.3 Guadecitabine and Durvalumab

Given the potential for long-term responses with immunotherapy but the relatively low response rate, there is an urgent need to identify combination therapies that result in response in a greater number of patients. Immune potentiation strategies, including epigenetic modifications to render more renal cell carcinoma tumors responsive to anti-PD-1 therapy, could dramatically increase the clinical benefit from anti-PD-L1 drugs [3].

DNA hypermethylation has been associated with tumor progression in renal cell carcinoma, and renal cell carcinomas with higher average methylation rates exhibit significantly higher stage and grade, and carry a worse prognosis [4]. Azacitidine is a nucleotide analog DNA demethylating agent that blocks the activity of DNA methyltransferases. Guadecitabine is a newer demethylating agent that has been shown to be clinically and biologically active [5].

Preclinical work at the University of Michigan in ovarian cancer cells and three renal cell carcinoma cell lines demonstrated that azacitidine and/or decitabine remove the methylation induced repression on the Th1-type chemokines CXCL9 and CXCL10 ([6] and Fig. 1, unpublished data). Treatment with azacitidine and Dznep, a histone methyltransferase inhibitor, led to higher levels of CXCL9 and

CXCL10 and enhanced cytotoxic CD8+T lymphocyte infiltration, resulting in tumor shrinkage in mice [6]. When an anti-PD-1 agent was combined with azacitidine and Dznep, the immune response was dramatically enhanced.

Figure 1. Treatment of 3 RCC cell lines with demethylating agent decitabine increases CXCL9 and CXCL10 levels

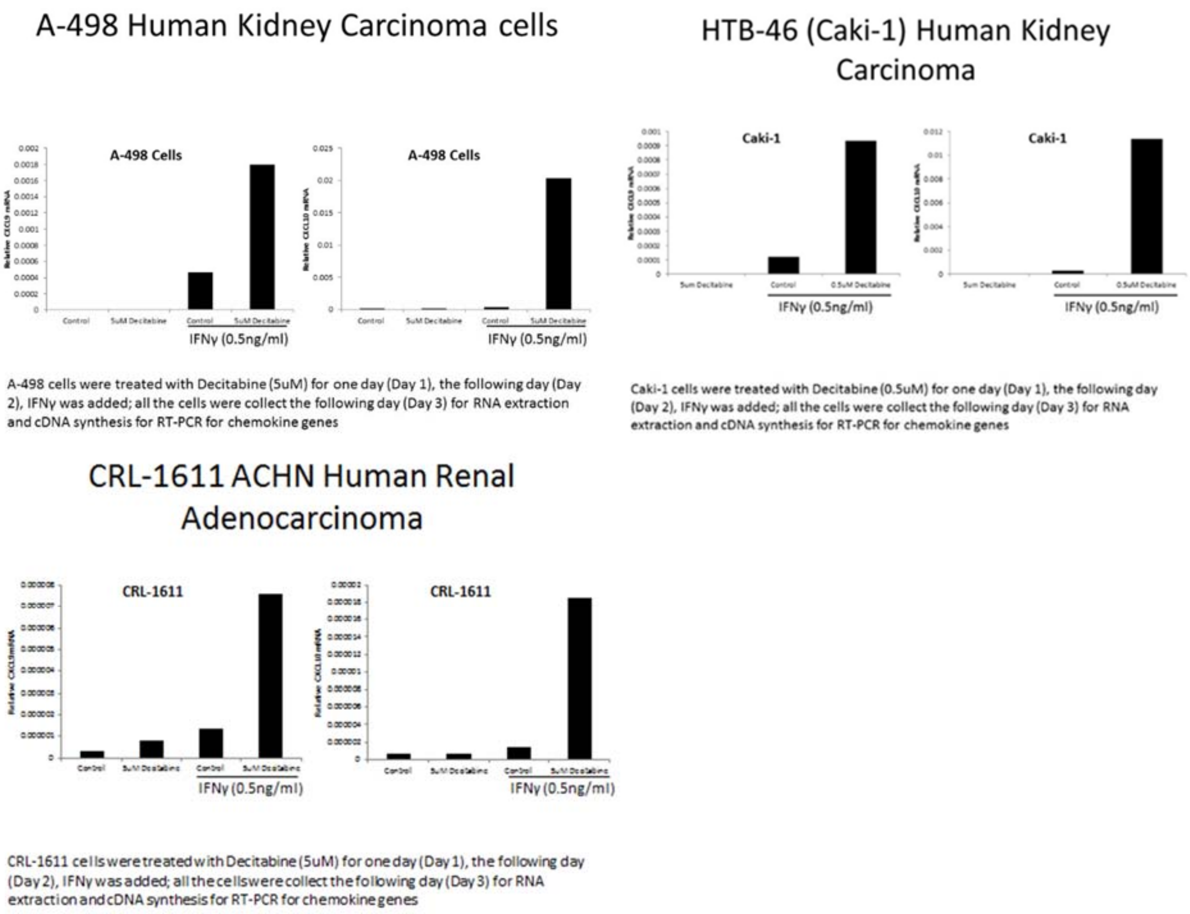

Guadecitabine Mechanism of Action, Efficacy and Safety
Guadecitabine is a potent inhibitor of DNA methylation that has been shown to induce a dose-dependent decrease of global DNA and gene-specific methylation in many different human cancer cell lines. Guadecitabine has been evaluated in AML, MDS, hepatocellular carcinoma and ovarian carcinoma. As monotherapy in the second line treatment of hepatocellular carcinoma (HCC), guadecitabine showed a >30% disease control rate. In a phase 1 trial of patients with platinum resistant ovarian cancer, the combination of guadecitabine and carboplatin led to a 15% objective response rate and a 50% clinical benefit rate. The most common adverse events seen in both solid tumor trials were: neutropenia (70-86%), fatigue (42-61%), anemia (38-41%), constipation (40-44%) and injection site events (46-50%). In the HCC trial, the initial dose tested was 60mg/m^2, however this was dose reduced to 45mg/m^2 due to grade ≥3 hematologic toxicity. At 45mg/m^2, 67% of patients had grade 3 neutropenia, 28% had grade 3 leukopenia, 10.9% had febrile neutropenia and 10.9% had lymphopenia.

Guadecitabine Pharmacokinetics, Metabolism, Excretion
Guadecitabine is given as a subcutaneous injection. After injection, the active metabolite decitabine is formed continuously, resulting in therapeutic exposures of decitabine (at guadecitabine doses ≥36 mg/m^2) throughout the mean extrapolated exposure window of 10.7 to 14.4 hours. In vitro studies in human hepatocytes suggest that guadecitabine is unlikely to inhibit or induce major human cytochrome P450 enzymes and is not a substrate for CYPs. Thus, P450-mediated drug-drug interactions are not anticipated for guadecitabine. Guadecitabine is predominantly excreted renally.

Durvalumab Mechanism of Action, Efficacy and Safety
The non-clinical and clinical experience is fully described in the most current version of the durvalumab Investigator's Brochure. Durvalumab is a human monoclonal antibody (mAb) of the immunoglobulin G (IgG) 1 kappa subclass that inhibits binding of PD-L1 and is being developed by AstraZeneca/MedImmune for use in the treatment of cancer. (MedImmune is a wholly owned subsidiary of AstraZeneca; AstraZeneca/MedImmune will be referred to as AstraZeneca throughout this document.) As durvalumab is an engineered mAb, it does not induce antibody-dependent cellular cytotoxicity or complement-dependent cytotoxicity. The proposed mechanism of action for durvalumab is interference of the interaction of PD-L1 with PD-1 and CD80. To date durvalumab has been given to more than 1800 subjects as part of ongoing studies either as monotherapy or in combination with other anti-cancer agents. Details on the safety profile of durvalumab monotherapy are summarized in Section 5.2.2.1 in the Investigator's brochure. Refer to the current durvalumab Investigator's Brochure for a complete summary of non-clinical and clinical information including safety, efficacy and pharmacokinetics.

The efficacy of durvalumab monotherapy has not yet been evaluated in metastatic renal cell carcinoma, although trials are ongoing in combination with the anti-CTLA4 drug, tremelimumab. Durvalumab monotherapy has been found to be efficacious in multiple solid tumors including urothelial carcinoma where it was found to have a 31% objective response rate [7].

Durvalumab Pharmacokinetics
A population PK model was developed for durvalumab using monotherapy data from a Phase I study (study 1108; N=292; doses= 0.1 to 10 mg/kg Q2W or 15 mg/kg Q3W; solid tumors). Population PK analysis indicated only minor impact of body weight (WT) on the PK of durvalumab (coefficient of ≤ 0.5). The impact of body WT-based (10 mg/kg Q2W) and fixed dosing (750 mg Q2W) of durvalumab was evaluated by comparing predicted steady state PK concentrations (5th, median and 95th percentiles) using the population PK model. A fixed dose of 750 mg was selected to approximate 10 mg/kg (based on median body WT of ~75 kg). A total of 1000 subjects were simulated using body WT distribution of 40–120 kg. Simulation results demonstrate that body WT-based and fixed dosing regimens yield similar median steady state PK concentrations with slightly less overall between-subject variability with fixed dosing regimen.

Similar findings have been reported by others ([8],[9], [10], [11]). Wang and colleagues investigated 12 monoclonal antibodies and found that fixed and body size-based dosing perform similarly, with fixed dosing being better for 7 of 12 antibodies [9] . In addition, they investigated 18 therapeutic proteins and peptides and showed that fixed dosing performed better for 12 of 18 in terms of reducing the between-subject variability in pharmacokinetic/pharmacodynamics parameters [10]

A fixed dosing approach is preferred by the prescribing community due to ease of use and reduced dosing errors. Given expectation of similar pharmacokinetic exposure and variability, we considered it feasible to switch to fixed dosing regimens. Based on average body WT of 75 kg, a fixed dose of 1500 mg Q4W durvalumab (equivalent to 20 mg/kg Q4W) is included in the current study.

1.4 Rationale

Anti-PD-1 monotherapy has been shown to be efficacious in the treatment of metastatic renal cell carcinoma. Hypomethylating agents have been shown to increase levels of CXCL9 and 10 in the tumor microenvironment, leading to recruitment of more T cells and tumor shrinkage in animal models. If more T cells were recruited to the tumor microenvironment, anti-PD-L1 therapy could effect greater tumor cell death.

We hypothesize that concurrent treatment with the anti-PD-1 drug, durvalumab and the hypomethylating agent, guadecitabine will synergistically enhance the anti-tumor immune response in patients with metastatic kidney cancer. We postulate that the combination of the two therapies would result in an increase in the objective response rate, our primary endpoint, as well as an improvement in the 2-year overall survival proportion, progression free survival, clinical benefit rate and complete response rate of patients with metastatic kidney cancer.

We propose a multi-site phase Ib/II trial of durvalumab in combination with guadecitabine in patients with metastatic kidney cancer through the Big Ten Cancer Research Consortium. Our trial will have two cohorts of patients. In Cohort 1, patients could have received up to 1 prior therapy but no prior anti-PD-1/PD-L1/CTLA4 drug. A smaller Cohort 2 will comprise patients who have received up to 2 prior therapies for advanced kidney cancer. One of these therapies must be a prior anti-PD-1/PD-L1 drug to which they did not respond.

2. STUDY OBJECTIVES AND ENDPOINTS

2.1 Objectives

2.1.1 Primary Objective

Phase Ib: To estimate the safety and toxicities of durvalumab in combination with guadecitabine in patients with metastatic clear cell renal cell carcinoma

Phase II: To assess the efficacy of durvalumab plus guadecitabine in first-or second-line therapy of patients with advanced RCC patients with no prior anti-PD-1/PD-L1/CTLA4 therapy (Cohort 1).

2.1.2 Secondary Objectives

1. To determine the clinical efficacy of patients treated with durvalumab plus guadecitabine in cohort 1 and separately in cohort 2.
2. To evaluate the safety of guadecitabine in combination with durvalumab in advanced RCC (Cohorts 1 and 2).

2.1.3 Correlative/Exploratory Objectives

1. To correlate changes in CXCL9, CXCL10 in tissue and serum as well as and LINE-1 methylation in blood with clinical response to the combination therapy
2. To correlate CD3+/CD8+ tumor infiltrating lymphocytes (TILs) and PD-L1 expression in baseline tumor tissue/TILs as well as immune cell subsets in the serum and tissue with clinical response to the combination therapy
3. To correlate tumor mutational burden and epigenetic changes with response to the combination therapy.
4. Analyze and correlate changes in methylation status and correlate tumor mutation profile in fresh tissue with response to the combination therapy

2.2 Endpoints

2.2.1 Primary Endpoint

Phase Ib Primary Endpoint: Number of patients with a dose-limiting toxicity (DLT).

Phase II Primary Endpoint: Objective response rate (CR + PR) as measured by RECIST 1.1 in Cohort 1 (patients with no prior anti-PD-1/PD-L1/CTLA4 therapy).

2.2.2 Secondary Endpoints

The Phase II secondary endpoints will be evaluated separately for cohort 1 and 2 and will be based on RECIST 1.1 unless otherwise specified. Secondary endpoints include:

1. 2-year overall survival proportion
2. Duration of Response (DoR)
3. Progression-free survival (PFS)
4. Clinical benefit rate (ORR+ stable disease for at least 6 months rate)
5. Complete response (CR) proportion
6. Objective response rate (CR + PR) as measured by the immune related response criteria (irRC)
7. Objective response rate (CR + PR) as measured by RECIST 1.1 in Cohort 2 (patients with prior anti-PD-1/ PD-L1 therapy)
8. Toxicity by CTCAE ver 4 including events of special interest such as immune mediated toxicities

2.2.3 Exploratory Endpoints

1. Correlation between baseline CXCL9 and CXCL10 tissue and serum levels and clinical response
2. Correlation between change in CXCL9 and CXCL10 serum levels and clinical response
3. Correlation between change in LINE-1 methylation in blood and clinical response
4. Correlation between degree of CD3+/CD8+ tumor infiltrating lymphocytes in tissue and clinical response
5. Correlation between degree of PD-L1 expression in tumor tissue and TILs and clinical response
6. Correlation between immune cell subsets in the serum and tissue and clinical response
7. Correlation between tumor mutational burden and epigenetic changes with clinical response

3. ELIGIBILITY CRITERIA

3.1 Inclusion Criteria

Subject must meet all of the following applicable inclusion criteria to participate in this study:

1. Written informed consent and HIPAA authorization for release of personal health information. **NOTE:** HIPAA authorization may be included in the informed consent or obtained separately.
2. Age ≥ 18 years at the time of informed consent.
3. ECOG Performance Status 0-1 within 28 days prior to registration.
4. Histological diagnosis of clear cell renal cell carcinoma (pure or mixed) with radiologic or histologic evidence of metastatic disease
5. At least 1 lesion, not previously irradiated that can be accurately measured at baseline as ≥ 10 mm in the longest diameter (except lymph nodes which must have a short axis ≥ 15mm) with a computed tomography (CT) or magnetic resonance imaging (MRI) and that is suitable for accurate repeated measurements as per Response Evaluation Criteria in Solid Tumors, version 1.1 (RECIST 1.1) guidelines
6. For **cohort 1**, subjects may have received up to 1 and no more than 1 prior line of systemic therapy (not counting any neoadjuvant/adjuvant therapy) including anti-VEGF, VEGFR inhibitor, MET inhibitor or mTOR inhibitor for metastatic disease. They cannot have received any prior anti-PD-1/PD-L1/CTLA4 therapy including durvalumab.
7. For **cohort 2**, subjects may have received up to 2 prior systemic lines of therapies (which should include 1 (and only 1) prior anti-PD-1/PD-L1 therapy) but did not have an objective response to the prior anti-PD-1/PD-L1 therapy. The treating investigator must document that the patient did not have an objective response to prior anti-PD-1/PD-L1 therapy. They may have received prior anti-CTLA4 therapy. Combination CTLA4 and anti-PD-1/PD-L1 therapy counts as one systemic line of therapy.
8. Subjects may not have had radiotherapy treatment to more than 30% of the bone marrow or with a wide field of radiation within 4 weeks of the first dose of study drug.
9. Prior cancer treatment must be completed at least 14 days prior to study registration and the subject must have recovered from all reversible acute toxic effects of the regimen (other than alopecia) to ≤Grade 1 or baseline.
10. Demonstrate adequate organ function as defined in the table below; all screening labs to be obtained within 28 days prior to registration.

System	Laboratory Value
**Hematological**	
White blood cell (WBC)	≥ 3 K/mm^3
Absolute Neutrophil Count (ANC)	≥ 1.5 K/mm^3
Hemoglobin (Hgb)	≥ 9 g/dL
Platelets (Plt)	≥ 100,000/mm^3
**Renal**	
Calculated creatinine clearance	≥ 40 cc/min using the Cockcroft-Gault formula
**Hepatic**	
Total Bilirubin	≤ 1.5 × upper limit of normal (ULN) except in cases of Gilbert's syndrome where the criteria will be ≤ 5 x ULN
Aspartate aminotransferase (AST)	≤ 2.5 × ULN
Alanine aminotransferase (ALT)	≤ 2.5 × ULN

11. Females of childbearing potential must have a negative serum pregnancy test within 28 days prior to registration. **NOTE:** Females are considered of childbearing potential unless they are surgically sterile (have undergone a hysterectomy, bilateral tubal ligation, or bilateral oophorectomy) or they are naturally postmenopausal for at least 12 consecutive months
    a. Women will be considered post-menopausal if they have been amenorrheic for 12 months without an alternative medical cause. The following age-specific requirements apply:
        i. Women <50 years of age would be considered post-menopausal if they have been amenorrheic for 12 months or more following cessation of exogenous hormonal treatments and if they have luteinizing hormone and follicle-stimulating hormone levels in the post-menopausal range for the institution or underwent surgical sterilization (bilateral oophorectomy or hysterectomy).
        ii. Women ≥50 years of age would be considered post-menopausal if they have been amenorrheic for 12 months or more following cessation of all exogenous hormonal treatments, had radiation-induced menopause with last menses >1 year ago, had chemotherapy-induced menopause with last menses >1 year ago, or underwent surgical sterilization (bilateral oophorectomy, bilateral salpingectomy or hysterectomy).
12. Females of childbearing potential and males must be willing to abstain from heterosexual activity or to use at least 1 highly effective methods of contraception from the time of informed consent until 180 days after treatment discontinuation. See section 5.8.
13. Life expectancy ≥ 12 weeks (in the opinion of the Investigator)
14. Body weight >30kg
15. Subject is willing and able to comply with the protocol for the duration of the study including undergoing treatment and scheduled visits and examinations including follow up.

3.2 Exclusion Criteria

Subjects meeting any of the criteria below may not participate in the study:

1. Active infection requiring systemic therapy
2. Brain metastases or spinal cord compression. Patients with suspected brain metastases at screening should have an MRI (preferred) or CT each preferably with IV contrast of the brain prior to study entry. Patients whose brain metastases have been treated may be considered if they have completed their treatment for brain metastasis at least 4 weeks prior to study registration provided they show radiographic stability (defined as 1 brain image, obtained after treatment to the brain metastases). In addition, any neurologic symptoms that developed either as a result of the brain metastases or their treatment must have resolved or be stable without the use of steroids for at least 14 days prior to the start of treatment.
3. Pregnant or breastfeeding (**NOTE:** breast milk cannot be stored for future use while the mother is being treated on study).
4. History of another primary malignancy except for:
    a. Malignancy treated with curative intent and with no known active disease ≥5 years before the first dose of IP and of low potential risk for recurrence
    b. Adequately treated non-melanoma skin cancer or lentigo maligna without evidence of disease
    c. Adequately treated carcinoma in situ without evidence of disease

5. Treatment with any investigational drug within 14 days prior to study registration
6. Active or prior documented autoimmune or inflammatory disorders (including inflammatory bowel disease [e.g., colitis or Crohn's disease], diverticulitis [with the exception of diverticulosis], celiac disease, irritable bowel disease, or other serious gastrointestinal chronic conditions associated with diarrhea, systemic lupus erythematosus, sarcoidosis syndrome, or Wegener syndrome [granulomatosis with polyangiitis, Graves' disease, rheumatoid arthritis, hypophysitis, uveitis, etc]) within the last 3 years prior to study registration. The following are exceptions to this criterion: The following are exceptions to this criterion:
   a. Subjects with vitiligo or alopecia
   b. Subjects with hypothyroidism (e.g., following Hashimoto syndrome) stable on hormone replacement
   c. Any chronic skin condition that does not require systemic therapy
   d. Subjects without active disease in the last 5 years may be included but only after consultation with the study physician
   e. Subjects with celiac disease controlled by diet alone
7. Current or prior use of immunosuppressive medication within 28 days before the first dose of durvalumab, with the exceptions of intranasal and inhaled corticosteroids or systemic corticosteroids at physiological doses, which are not to exceed 10 mg/day of prednisone, or an equivalent corticosteroid. The following are exceptions to this criterion:
   - Intranasal, inhaled, topical steroids, or local steroid injections (eg, intra articular injection)
   - Systemic corticosteroids at physiologic doses not to exceed 10 mg/day of prednisone or its equivalent
   - Steroids as premedication for hypersensitivity reactions (eg, CT scan premedication)
8. Involvement in the planning and/or conduct of the study (applies to both AstraZeneca/ MedImmune staff and/or staff at the study site).
9. Concurrent enrollment in another clinical study, unless it is an observational study or intervention with non-drug/non-therapeutic agent(s) or during the follow-up period of an interventional study.
10. Any concurrent chemotherapy, biologic or hormonal therapy for cancer treatment. Concurrent use of hormonal therapy for non-cancer-related conditions (e.g. hormone replacement therapy) is acceptable. Note: Local treatment of isolated lesions, excluding target lesions, for palliative intent is acceptable (e.g., local surgery or radiotherapy)
11. Major surgical procedure (as defined by the Investigator) within 28 days prior to study registration. Note: local surgery of isolated lesions for palliative intent is acceptable
12. History of allogenic organ transplantation that requires use of immunosuppressive agents
13. Uncontrolled intercurrent illness including but not limited to, ongoing or active infection, symptomatic congestive heart failure, uncontrolled hypertension, unstable angina pectoris, cardiac arrhythmia, interstitial lung disease, or psychiatric illness/social situations that would limit compliance with study requirement, substantially increase risk of incurring AEs or compromise the ability of the patient to give written informed consent
14. History of leptomeningeal carcinomatosis.

15. Active infection including **tuberculosis** (clinical evaluation that includes clinical history, physical examination and radiographic findings), **hepatitis B** (known positive HBV surface antigen (HBsAg) result), **hepatitis C**, or **human immunodeficiency virus** (positive HIV 1/2 antibodies). Subjects with a past or resolved HBV infection (defined as the presence of hepatitis B core antibody [anti-HBc] and absence of HBsAg) are eligible. Subjects positive for hepatitis C (HCV) antibody are eligible only if polymerase chain reaction is negative for HCV RNA.
16. Receipt of live attenuated vaccine within 30 days prior to the first dose of study drug. Note: patients, if enrolled, should not receive live vaccine during the study and up to 30 days after the last dose of study drug.
17. Known allergy or hypersensitivity to study drugs or other humanized monoclonal antibodies.
18. Patient ≤30kg in weight.
19. Mean QT interval corrected for heart rate (QTc) ≥470 ms calculated from 3 electrocardiograms (ECGs) using Fridericia's Correction
20. Any unresolved toxicity NCI CTCAE Grade ≥2 from previous anticancer therapy with the exception of alopecia, vitiligo, and the laboratory values defined in the inclusion criteria
    a. Subjects with Grade ≥2 neuropathy will be evaluated on a case-by-case basis after consultation with the Study Physician.
    b. Subjects with irreversible toxicity not reasonably expected to be exacerbated by treatment with durvalumab may be included only after consultation with the Study Physician.
21. Past medical history of ILD, drug-induced ILD, radiation pneumonitis which required steroid treatment, or any evidence of clinically active interstitial lung disease

4. SUBJECT REGISTRATION

All subjects must be registered through Big Ten CRC Administrative Headquarters' electronic data capture (EDC) system. A subject is considered registered when an On Study date is entered into the EDC.

Protocol treatment must start within 14 business days of enrollment to the study.

5. TREATMENT PLAN

5.1 Study Design

This is a non-randomized, single arm, open label study of durvalumab in combination with guadecitabine in subjects with metastatic clear cell renal cell carcinoma.

5.2 Number of Subjects

A total of up to 58 subjects will be enrolled on both phases.

Phase Ib: 6 subjects; enrolled into either Cohort 1 or 2.
Phase II: 52 subjects; enrolled into either Cohort 1 or 2.

Cohort 1 (36 subjects): received 0-1 prior therapy and no prior anti-PD-1/PD-L1/CTLA4.
Cohort 2 (16 subjects): received up to 2 prior therapies, one of which must include an anti-PD-1/PD-L1 therapy to which they did not respond. Only one prior anti-PD-1/PD-L1 therapy is allowed.

Patients from Phase Ib treated at the eventual recommended phase II dose will be combined with patients in Phase II in the efficacy analysis.

- Therapy will start with guadecitabine on days 1-5 of a 28-day cycle. Guadecitabine will be dosed subcutaneously on days 1-5 at the recommended phase II dose.
- Durvalumab will be started on day 8 of the 28-day cycle. Durvalumab will be administered intravenously at a flat dose of 1500mg every 28 days.
- There must be a gap of at least 24 hours (48 hours preferred) between guadecitabine and durvalumab administration.
- Study treatment may continue until confirmed progression, intolerable toxicity, or withdrawal of consent.

5.3 Phase Ib Treatment Plan

- Dose limiting toxicities (DLTs) will be evaluated within the first cycle (i.e., within the first 28 days).
- Six patients will be enrolled at dose level 0. If 2 or fewer patients experience a dose limiting toxicity, the study will continue to the phase II portion.
- Alternately, if 3 or more patients have a dose limiting toxicity at dose level 0, 6 patients will be accrued at the lower dose (dose -1). If 2 or fewer patients experience a dose limiting toxicity, the study will continue to phase II at dose level -1.
- If 3 or more subjects experience a dose limiting toxicity at dose level -1, the treatment will be considered unsafe and the trial will be stopped. In this case, durvalumab and guadecitabine will be permanently discontinued and the subjects followed per protocol.

<table>
<tr><th colspan="5">Treatment Plan for Phase Ib portion</th></tr>
<tr><th>Dose Level</th><th>Guadecitabine dose[1, 2]</th><th>Durvalumab dose[2]</th><th>Number of Patients</th><th>Cycle Length</th></tr>
<tr><td>Level 0 (start)</td><td>60 mg/m^2 subcutaneously (Days 1-5)</td><td rowspan="2">1500mg IV (Day 8)</td><td>6</td><td rowspan="2">4 weeks (28 days)</td></tr>
<tr><td>Level -1 (if needed)</td><td>45 mg/m^2 subcutaneously (Day 1-5)</td><td>6</td></tr>
<tr><td colspan="5">[1] Body surface area (BSA) should be recalculated when weight changes by ≥ 10% according to the Mosteller formula.
[2] A window of ± 3 days may be applied to all study visits to accommodate observed holidays, inclement weather, scheduling conflicts etc. However, there must be a gap of at least 24 hours (48 hours preferred) between guadecitabine and durvalumab administration. Date and time of each drug administration should be clearly documented in subject’s chart and electronic case report forms (eCRFs).</td></tr>
</table>

5.4 Dose-Limiting Toxicity (DLT):

A DLT will be defined as any Grade 3 or higher toxicity that occurs during the DLT evaluation period. Toxicity that is clearly and directly related to the primary disease or to another etiology is excluded from this definition. The following will be DLTs:

- Any Grade 4 imAE (immune mediated AE)
- Any ≥ Grade 3 colitis
- Any Grade 3 or 4 noninfectious pneumonitis irrespective of duration
- Any Grade 2 pneumonitis that does not resolve to ≤ Grade 1 within 3 days of the initiation of maximal supportive care
- Any Grade 3 imAE, excluding colitis or pneumonitis, that does not downgrade to Grade 2 within 3 days after onset of the event despite optimal medical management including systemic corticosteroids or does not downgrade to ≤ Grade 1 or baseline within 14 days
- Liver transaminase elevation > 8 × ULN or total bilirubin > 5 × ULN
- Grade 4 neutropenia lasting >7 days
- Grade 4 thrombocytopenia
- Any grade febrile neutropenia
- ALT or AST >3x ULN AND a serum total bilirubin >2 × ULN AND alkaline phosphatase <2 × ULN without any other clear etiology aside from being related to the trial drug(s) (Hy’s Law)
- Any grade 3 or higher injection site reaction
- Any ≥ Grade 3 non-imAE, except for the exclusions listed below

The DLT definition excludes the following conditions:

- Grade 3 fatigue lasting ≤ 7 days
- Grade 3 endocrine disorder (thyroid, pituitary, and/or adrenal insufficiency) that is managed with or without systemic corticosteroid therapy and/or hormone replacement therapy and the subject is asymptomatic
- Concurrent vitiligo or alopecia of any AE grade

- Grade 3 infusion-related reaction (first occurrence and in the absence of steroid prophylaxis) that resolves within 6 hours with appropriate clinical management
- Grade 3 or 4 neutropenia that is not associated with fever or systemic infection that improves by within 7 calendar days. Any grade febrile neutropenia will be a DLT regardless of duration or reversibility
- Grade 3 or 4 lymphopenia
- Grade 3 thrombocytopenia that is not associated with clinically significant bleeding that requires medical intervention, and improves by at least 1 grade within 3 calendar days
- Isolated Grade 3 electrolyte abnormalities that are not associated with clinical signs or symptoms and are reversed with appropriate maximal medical intervention within 3 calendar days
- Grade 3 laboratory abnormalities that are responsive to oral supplementation or deemed by the investigator to be clinically non-significant
- Grade 3 nausea/vomiting or diarrhea < 72 hours with adequate antiemetic and other supportive care

A withdrawal or death within the first cycle (28 days) *not* related to treatment will not be considered a DLT.

DLTs will be counted based on the number of subjects with DLT at a given dose level, not the absolute number of DLTs. No single subject can trigger more than one DLT event.

NCI CTCAE version 4 will be used for all grading.

5.5 Treatment Plan for the Phase II Portion

Based on the toxicities observed in the Phase Ib subjects and new data from other ongoing clinical trials with guadecitabine, the recommended phase II dose of guadecitabine will be 45 mg/m^2. Although formal DLT definitions were not met in the phase Ib portion, three of five Phase Ib subjects who received dose of 60 mg/m^2/d guadecitabine were found to have significant neutropenia (grade 3 or 4) on Cycle 2 Day 1 requiring delay/skipping of Cycle 2 guadecitabine. Data from the other guadecitabine studies in combination with durvalumab (e.g. Guadecitabine and Durvalumab in Treating Patients With Advanced Liver, Pancreatic, Bile Duct, or Gallbladder Cancer [NCT03257761]; SGI-110 Plus Durvalumab/ Tremelimumab in SCLC [NCT03085849]) support a 45 mg/m^2 dose. Therefore, the Phase II starting dose of guadecitabine will be 45 mg/m^2 subcutaneous daily on days 1-5 with a fixed dose of durvalumab at 1500 mg IV on day 8.

Treatment Plan for Phase II portion		
**Guadecitabine dose[1,2]**	**Durvalumab dose[2]**	**Cycle Length**
45 mg/m^2 subcutaneously (Day 1-5)	1500mg IV (Day 8)	4 weeks (28 days)
[1] Body surface area (BSA) should be recalculated when weight changes by ≥ 10% according to the Mosteller formula.<br>[2] A window of ± 3 days may be applied to all study visits to accommodate observed holidays, inclement weather, scheduling conflicts etc. However, there must be a gap of at least 24 hours (48 hours preferred) between guadecitabine and durvalumab administration. Date and time of each drug administration should be clearly documented in subject's chart and electronic case report forms (eCRFs).		

5.6 Pre-medication and Hydration

Pre-medication is not required for guadecitabine or durvalumab but may be administered per physician discretion. Emla (Lidocaine / Prilocaine) cream topically and/or ice packs before or after guadecitabine injection are suggested to alleviate local injection site discomfort.

5.6.1 Monitoring during durvalumab infusion

Subjects will be monitored before, during and after the infusion with assessment of vital signs at the times described below.

In the event of a ≤Grade 2 infusion-related reaction, the infusion rate of study drug may be decreased by 50% or interrupted until resolution of the event (up to 4 hours) and re-initiated at 50% of the initial rate until completion of the infusion. For subjects with a ≤Grade 2 infusion-related reaction, subsequent infusions may be administered at 50% of the initial rate. Acetaminophen and/or an antihistamine (e.g., diphenhydramine) or equivalent medications per institutional standard may be administered at the discretion of the investigator. If the infusion-related reaction is Grade 3 or higher in severity, study drug will be discontinued.

The standard infusion time is one hour, however if there are interruptions during infusion, the total allowed time from infusion start to completion of infusion should not exceed 8 hours at room temperature, with maximum total time at room temperature prior to start of infusion not exceeding 4 hours (otherwise requires new infusion preparation). For management of subjects who experience an infusion reaction, please refer to the toxicity and management guidelines.

First infusion

On the first infusion day, patient will be monitored and vital signs collected/recorded in eCRF before, during and after infusion as presented in the bulleted list below.

- Before the start of the infusion (between -30 minutes to 0 minutes [i.e., start of the infusion])
- During the infusion (30 minutes ±5 minutes [i.e., halfway through infusion])
- After the end of the infusion (60 minutes ±5 minutes)
- If the infusion takes longer than 60 minutes, BP and pulse measurements should follow the principles as described above or be taken more frequently if clinically indicated.

Subsequent infusions

BP, pulse and other vital signs should be measured, collected/recorded in eCRF prior to the start of the infusion. Patients should be carefully monitored and BP and other vital signs should be measured during and post infusion as per institution standard and as clinically indicated. Any clinically significant changes in vital signs should be entered onto an unscheduled vital signs CRF page.

5.7 Concomitant Medications

5.7.1 Allowed Concomitant Medications

All treatments the investigator considers necessary for a subject's welfare may be administered at the discretion of the investigator in keeping with the community standards of medical care.

Table 1. Supportive Medications

Supportive medication/class of drug:	Usage:
Concomitant medications or treatments (e.g., acetaminophen or diphenhydramine) deemed necessary to provide adequate prophylactic or supportive care, except for those medications identified as "prohibited," as listed below	To be administered as prescribed by the Investigator
Best supportive care (including antibiotics, nutritional support, correction of metabolic disorders, optimal symptom control, and pain management [including palliative radiotherapy to non-target lesions, etc])	Should be used, when necessary, for all subjects
Inactivated viruses, such as those in the influenza vaccine	Permitted

5.7.2 Prohibited Concomitant Medications

Prohibited medication/class of drug:	Usage:
Any investigational anticancer therapy other than those under investigation in this study	Should not be given concomitantly whilst the patient is on study treatment
mAbs against CTLA-4, PD-1, or PD-L1 other than those under investigation in this study	Should not be given concomitantly whilst the patient is on study treatment
Any concurrent chemotherapy, radiotherapy, immunotherapy, or biologic or hormonal therapy for cancer treatment other than those under investigation in this study	Should not be given concomitantly whilst the patient is on study treatment. (Concurrent use of hormones for non-cancer-related conditions [e.g., insulin for diabetes and hormone replacement therapy] is acceptable. Local treatment of isolated lesions, excluding target lesions, for palliative intent is acceptable [e.g., by local surgery or radiotherapy])
Immunosuppressive medications including, but not limited to, systemic corticosteroids at doses exceeding10 mg/day of prednisone or equivalent, methotrexate, azathioprine, and tumor necrosis factor-α blockers	Should not be given concomitantly. (Use of immunosuppressive medications for the management of IP-related AEs, or in subjects with contrast allergies is acceptable). In addition, use of inhaled, topical, and intranasal corticosteroids is permitted.
EGFR TKIs	Should not be given concomitantly.<br>Should be used with caution in the 90 days post last dose of durvalumab.<br>Increased incidences of pneumonitis (with third generation EGFR TKIs) and increased incidence of transaminase increases (with 1st generation EGFR TKIs) has been reported when durvalumab has been given concomitantly.
Live attenuated vaccines	Should not be given through 30 days after the last dose of IP (including SoC)

There are no prohibited therapies during the Long-Term Follow-up Phase.

5.8 Contraception

The following restrictions apply while the patient is receiving study treatment and for the specified times before and after:

<u>Female patient of child-bearing potential</u>

- Females of childbearing potential who are sexually active with a non-sterilized male partner must use at least 1 **<u>highly</u>** effective method of contraception from the time of screening and must agree to continue using such precautions for 180 days after the last dose of durvalumab + guadecitabine. Non-sterilized male partners of a female patient must use male condom plus spermicide throughout this period. Cessation of birth control after this point should be discussed with a responsible physician. Not engaging in sexual activity for the total duration of the drug treatment and the drug washout period is an acceptable practice; however, periodic abstinence, the rhythm method, and the withdrawal method are not acceptable methods of birth control. Female subjects should also refrain from breastfeeding throughout this period.

Male subjects with a female partner of childbearing potential

- Non-sterilized males who are sexually active with a female partner of childbearing potential must use a male condom plus spermicide from screening through 180 days after receipt of the final dose of durvalumab + guadecitabine or. Not engaging in sexual activity is an acceptable practice; however, occasional abstinence, the rhythm method, and the withdrawal method are not acceptable methods of contraception. Male subjects should refrain from sperm donation throughout this period.
- Female partners (of childbearing potential) of male subjects must also use a highly effective method of contraception throughout this period.

Females of childbearing potential are defined as those who are not surgically sterile (i.e., bilateral tubal ligation, bilateral oophorectomy, or complete hysterectomy) or post-menopausal.

Women will be considered post-menopausal if they have been amenorrheic for 12 months without an alternative medical cause. The following age-specific requirements apply:

- Women <50 years of age would be considered post-menopausal if they have been amenorrheic for 12 months or more following cessation of exogenous hormonal treatments and if they have luteinizing hormone and follicle-stimulating hormone levels in the post-menopausal range for the institution or underwent surgical sterilization (bilateral oophorectomy or hysterectomy).
- Women ≥50 years of age would be considered post-menopausal if they have been amenorrheic for 12 months or more following cessation of all exogenous hormonal treatments, had radiation-induced menopause with last menses >1 year ago, had chemotherapy-induced menopause with last menses >1 year ago, or underwent surgical sterilization (bilateral oophorectomy, bilateral salpingectomy or hysterectomy).

Highly effective methods of contraception, defined as one that results in a low failure rate (i.e., less than 1% per year) when used consistently and correctly are described in the table below. Note that some contraception methods are not considered highly effective (e.g. male or female condom with or without spermicide; female cap, diaphragm, or sponge with or without spermicide; non-copper containing intrauterine device; progestogen-only oral hormonal contraceptive pills where inhibition of ovulation is not the primary mode of action; and triphasic combined oral contraceptive pills).

Highly Effective Methods of Contraception (<1% Failure Rate)

• Barrier/Intrauterine methods	• Hormonal Methods
• Copper T intrauterine device<br>• Levonorgestrel-releasing intrauterine system (e.g., Mirena®)[a]	• Etonogestrel implants: e.g. Implanon<br>• Intravaginal device: e.g. ethinylestradiol and etonogestrel<br>• Medroxyprogesterone injection: e.g. Depo-Provera<br>• Normal and low dose combined oral contraceptive pill<br>• Norelgestromin/ethinylestradiol transdermal system

a This is also considered a hormonal method

5.9 Blood donation

Subjects should not donate blood while participating in this study, or for at least 90 days following the last infusion of durvalumab.

6. TOXICITIES AND DOSE DELAYS/DOSE MODIFICATIONS

The NCI Common Terminology Criteria for Adverse Events (CTCAE) v4 will be used to grade adverse events.

Subjects enrolled in this study will be evaluated clinically and with standard laboratory tests before and at regular intervals during their participation in this study as specified in Study Calendar & Evaluations.

Subjects will be evaluated for adverse events (all grades), serious adverse events, and adverse events requiring study drug interruption or discontinuation as specified in Study Calendar & Evaluations.

6.1 Dose Delays/Dose Modifications

A new guadecitabine cycle will only be initiated when ALL of the following conditions are met:

1. ANC $\geq$ 1,000/mm^3
2. Platelets $\geq$ 75,000/mm^3
3. Hemoglobin $\geq$ 9 g/dL
   a. Transfusions are permitted
4. Non-hematologic treatment related toxicities have improved to $\leq$ Grade 1 or to the subject's baseline values (the following toxicities are excluded from this requirement: alopecia, hypothyroidism and vitiligo)

- If blood counts are below the above threshold on day 1 of the planned cycle, the start of that cycle will be delayed until blood counts are acceptable. Up to 7 calendar days delay (±3 calendar days) is allowed.
- If guadecitabine is held on a scheduled cycle start date, blood work (CBC with platelets and diff, COMP) is to be repeated weekly until counts recover to satisfy above parameters in 6.1. Additional testing may be performed per standard of care or as clinically indicated.

- If guadecitabine is unable to be resumed within 7 calendar days (±3 days) of the original planned treatment date, guadecitabine therapy will be dose reduced per the table in 6.2.
- If guadecitabine is delayed for more than 7 calendar days for hematologic reasons, durvalumab can resume every 4 weeks as previously scheduled as long as durvalumab related toxicities (except hypothyroidism and vitiligo) have recovered to grade 1 or better.

6.2 Dose Levels/ Dose Reductions

6.2.1 Dose Reductions for Guadecitabine

No dose reductions are required for non-hematologic toxicity. Hematologic toxicities should be managed as per the below table:

**Dose Reductions for Guadecitabine**<br>Counts based on CBC on the day the cycle was skipped		
**ANC**	**Platelets**	**Action**
≥ 1,000/mm^3	>75,000/ mm^3	None
<999/mm^3 or	≤75,000/ mm^3	Restart next cycle at 1 dose level below current dose to a minimum dose of 30 mg/m^2
Note:<br>**G-CSF (Filgrastim) or Neulasta® are not to be given empirically but can be added at the discretion of the investigator in the setting of neutropenic fever with prior cycles or neutropenia requiring a guadecitabine dose reduction.<br>***If a patient's Day1 counts are below the threshold to be treated with guadecitabine 30mg/m^2, they must discontinue treatment due to grade 4 toxicity		

Phase II Dose Levels for Guadecitabine	Dose
0	45 mg/m^2
-1	30 mg/m^2

Drug administration modifications of durvalumab will be made to manage potential immune-mediated AEs based on severity of treatment-emergent toxicities graded per NCI CTCAE v4.

6.2.2 Dose Reductions for Durvalumab

It is recommended that management of immune mediated adverse events (imAEs) follow the guidelines in Appendix I and presented below:

- Patients should be thoroughly evaluated to rule out any alternative etiology (e.g., disease progression, concomitant medications, and infections).
- More potent immunosuppressives such as TNF inhibitors (e.g., infliximab) should be considered for events not responding to systemic steroids.

6.3 Protocol Therapy Discontinuation

In addition to discontinuation from therapy related to toxicities as outlined in section 6.1, a subject will also be discontinued from protocol therapy and followed up per protocol under the circumstances outlined below. The reason for discontinuation of protocol therapy will be documented on the electronic case report form (eCRF).

- Confirmed disease progression (or recurrence for previous CR patients) and investigator determination that the patient is no longer benefitting from protocol therapy
  - Treatment with guadecitabine and durvalumab beyond one confirmed progression is permitted after discussion with the sponsor investigator. See 6.4 below.
  -
  - To treat a patient beyond progression please contact the Big Ten CRC Project Manager. Patients will need to be re-consented to receive treatment with guadecitabine and durvalumab beyond progression.
  - To treat beyond progression, criteria must be met to ensure that patients are not exposed to unreasonable risks by continued use of guadecitabine and durvalumab in spite of progression of disease. These criteria include the following:
    - Absence of symptoms or signs indicating clinically significant progression of disease
    - No decline in ECOG status or Karnofsky Performance Status
    - Absence of symptomatic rapid disease progression requiring urgent medical intervention (e.g., symptomatic pleural effusion, spinal cord compression)
- The treating physician thinks a change of therapy would be in the best interest of the subject
- The subject requests to discontinue protocol therapy, whether due to unacceptable toxicity or for other reasons
  - If a subject decides to prematurely discontinue protocol therapy (“refuses treatment”), the subject should be asked if he or she may still be contacted for further scheduled study assessments. The outcome of that discussion should be documented in both the medical records and in the eCRF.
- A female subject becomes pregnant
- If protocol therapy is interrupted for ≥ consecutive 84 calendar days.
- An individual patient will not receive any further durvalumab if their weight falls to 30kg or less
- Withdrawal of consent of lost to follow-up
- Adverse event that, in the opinion of the investigator or the sponsor, contraindicates further dosing
- Subject is determined to have met one or more of the exclusion criteria for study participation at study entry and continuing investigational therapy might constitute a safety risk
- Grade ≥ 3 infusion reaction

6.4 Criteria for Continuing Study Treatment Beyond 1st Progression:

All treatment will be administered beginning on Day 1 until clinical progression or RECIST 1.1-defined radiological progression (See Section 9) unless there is unacceptable toxicity, withdrawal of consent, or another discontinuation criterion is met.

Subjects who are clinically stable at the first RECIST 1.1 -defined PD may opt to continue to receive study treatment beyond the first progression at the discretion of the Investigator and after providing signed informed consent if they fulfill all the criteria below.

Study treatment beyond 1st progression criteria-all must be fulfilled:

- The subject does not have any significant, unacceptable, or irreversible toxicities that indicate continuing treatment will not further benefit the subject. The subject must not have experienced a toxicity that required permanent discontinuation of study treatment. Moreover, subject should have no ongoing or residual toxicity at the time of continuing treatment beyond 1st progression attributed as possible, probable or definite to study therapy greater than Grade 1 (except hypothyroidism, vitiligo, fatigue).
- There is absence of clinical symptoms or signs indicating clinically significant disease progression or accompanied by a decline in ECOG performance status to >1. Patients with rapid tumor progression or with symptomatic progression that requires urgent medical intervention (e.g., central nervous system metastasis, respiratory failure due to tumor compression, or spinal cord compression) will not be eligible for continuing durvalumab and guadecitabine.
- There is absence of rapid disease progression or threat to vital organs or critical anatomical sites (e.g., central nervous system metastasis, respiratory failure due to tumor compression, or spinal cord compression) requiring urgent alternative medical intervention
- The patient still fulfills the eligibility criteria for this study (see Section 5.1 and 5.2).
- Subjects must provide informed consent to receive the treatment beyond 1st progression.

Patients on study meeting the above criteria for continuing beyond 1st progression will follow the same treatment guidelines followed during the original treatment period, including the same dose and frequency of treatments and the same schedule of assessments.

Patients who meet the criteria for continuing beyond 1st progression may only receive treatment beyond 1st progression until the second progression, if applicable, and cannot continue beyond a second progression under any circumstances.

For all patients who are treated through progression, the Investigator should ensure patients do not have any significant, unacceptable, or irreversible toxicities that indicate continuing therapy would not further benefit the patient.

6.5 Protocol Discontinuation

If a subject decides to withdraw from the study (and not just from protocol therapy) all efforts should be made to complete the final study assessments. The site study team should contact the subject by telephone or through a clinic visit to determine the reason for the study withdrawal. If the reason for withdrawal is an adverse event, it will be recorded on the eCRF.

6.6 Duration of Protocol Therapy

A subject may continue on protocol therapy until progression or any of the criteria listed in 6.3.

7. STUDY CALENDAR & EVALUATIONS

	Screen	Cycle 1 [2]				Cycle 2 [2]				Cycle 3+[2]		Safety follow up	Long-term Follow up[5]
Cycle = 28 days	-28 days[1]	D1[1]	D8	D15	D21	D1	D8	D15	D21	D1	D8	+30 days[4]	Q 3 mo. (±14 days)
**REQUIRED ASSESSMENTS**													
Consent; Med Hx; Demographics; Dx; Staging	X												
Review Subject Eligibility Criteria	X												
Physical exam	X	X	X			X				X		X	
Vital signs, ECOG Performance status[6]	X	X	x3			X				X		X	
ECG[13]	X		x2										
AEs, AESIs & concomitant medications	X	X	X			X				X		X	
**LABORATORY ASSESSMENTS**													
Complete Blood Cell Count with diff (CBC)	X	X	X	X	X	X	X	X	X	X	X	X	
Comprehensive Metabolic Profile (CMP)[7]	X	X	X	X		X	X	X		X	X	X	
LDH, Thyroid Function[8]	X					X				X		X	
Amylase & Lipase	X					X				X			
Hepatitis B, Hepatitis C, HIV	X												
Pregnancy test (serum or urine) WOCBP	-7 d[9]												
**DISEASE ASSESSMENT[3,5]**													
CT of chest	X[3]									Q 2 cycles[3]			
CT or MRI of abdomen and pelvis	X[3]									Q 2 cycles[3]			
Bone scan	X[3]									Q 2 cycles[3]			
CT Head or brain MRI	X[3]												
**TREATMENT EXPOSURE**													
Guadecitabine (Days 1-5)		X				X				X			
Durvalumab			X				X				X[5]		
**CORRELATIVE STUDIES (SPECIMEN COLLECTION)**													
Archival tumor tissue[10]	X												
Fresh biopsy- optional												@PD	
**U. Michigan only**--Fresh biopsy- optional[14]	X									Pre-C3			
**U. Michigan only**--whole blood- optional[14]	X												
Blood for CXCL9/CXCL10		X	X				X						
Blood for methylation status		X	X				X						
**BANKING SAMPLES (SPECIMEN COLLECTION)**													
Whole Blood[11], Serum and Plasma[12]		X											
**FOLLOW-UP**													
Survival status													X

Key to Footnotes

[1]If screening (baseline) labs were performed within 7 days of D1 of treatment, these do not need to be repeated.

[2]A window of 3 business days will be applied to all treatment study visits; for safety follow-up visit and tumor imaging, a 7-day window will apply.

[3]Tumor response assessment will be performed every 2 cycles (8 weeks) (pre-treatment C3, 5, 7, etc.). Baseline bone scan will be obtained if there is a suspicion of metastatic bone involvement. If bone scan is positive at baseline, it will be included with subsequent tumor response assessments as noted above. MRI of brain is required only at screening to evaluate for the presence of brain metastases. After the screening assessment, brain imaging is optional and will be done if clinically indicated at the discretion of the investigator.

[4]A safety follow-up visit will occur 30 days (±7 days) after the last dose of treatment. [AESIs and SAEs will be collected for 90 days after the end of treatment. See Sections 7.3 and 11.2.]

[5]Once disease progression is documented or have stopped protocol specified therapy due to intolerable toxicities (whichever occurs first), subjects will enter a survival follow up period every 3 months for 2 years from the time of registration. For patients who have stopped all protocol specified therapy, scans and/or labs will be per the treating investigator's discretion/standard of care and will not be dictated by protocol. At a minimum, survival follow up will be done every 3 months via phone or other form of communication.

[6]Vital signs to include blood pressure, weight, and height (screening only) and ECOG performance status. See 7.2.2 for VS during Cycle 1 Day 8.

[7]CMP to include sodium, potassium, chloride, creatinine, blood urea nitrogen; liver function tests (LFTs) to include AST, ALT, total bilirubin, alkaline phosphatase

[8]Thyroid function to include TSH, T4, free T3 and if clinically indicated FSH, LH, ACTH

[9]For women of childbearing potential (WOCBP): urine or serum β HCG, within 7 days prior to study registration. If a urine test is positive or cannot be confirmed as negative, a serum pregnancy test will be required.

[10]An archived tissue block or a minimum of 15 unstained slides of 4 micron thickness each must be identified to submit (but not needed to be submitted or received) prior to registration. See CLM for collection, processing, labeling and shipping instructions.

[11]Whole blood for banking is to be collected at Pre-Treatment Cycle 1 Day 1. See CLM for collection, processing, labeling and shipping instructions.

[12]Serum and plasma for banking are to be collected at Pre-Treatment Cycle 1 Day 1. See CLM for collection, labeling, processing, and shipping instructions.

[13] 12-lead ECG. Resting 12-lead ECGs will be recorded at screening, pre and post C1D8, and as clinically indicated throughout the study. ECGs should be obtained after the patient has been in a supine position for 5 minutes and recorded while the patient remains in that position. ECGs at pre- Cycle 1 Day 8 to be performed within 1 hr prior to the start of the first study treatment. **ECG at post- Cycle 1 Day 8 should be taken 0-3hrs after administration of durvalumab is completed for the first cycle only.** Thereafter as clinically indicated. Baseline and abnormal ECG at any time to be performed in triplicate in triplicate 2-5 minutes apart, others single.

[14] **University of Michigan only**: optional fresh tissue biopsy for correlative studies will be performed at baseline and pre-treatment Cycle 3 Day 1. Whole blood for somatic baseline will be collected anytime prior to treatment on Cycle 1 Day 1 in subjects who have consented to fresh tissue biopsies.

7.1 Screening Evaluations

7.1.1 Within 28 days prior to registration for protocol therapy

All screening procedures must be performed within 28 days prior to registration unless otherwise stated. The screening procedures include:

- Informed Consent
- Medical history: Complete medical and surgical history, history of infections
- Demographics: Age, gender, race, ethnicity, trial awareness question
- Diagnosis and staging
- Review subject eligibility criteria
- Physical exam
- Vital signs (temperature, pulse, respirations, blood pressure), height (screening only) and weight
- ECOG Performance status
- ECG in triplicate 2-5 minutes apart
- Adverse event assessment
- Review concomitant medications
- CBC with diff to include: total WBC, hemoglobin, hematocrit, platelet count and differential of the WBC including absolute counts
- Comprehensive metabolic panel (CMP) to include: albumin, alkaline phosphatase, ALT/SGPT, AST/SGOT, BUN, creatinine, electrolytes (sodium, potassium, calcium, chloride, bicarbonate), glucose, and total bilirubin.
- Lactate dehydrogenase (LDH)
- Thyroid function: thyroid-stimulating hormone (TSH), free triiodothyronine (free T3), free triiodothyronine (free T4). If clinically indicated: follicle-stimulating hormone (FSH), luteinizing hormone (LH), adrenocorticotropic hormone (ACTH).
- Amylase, Lipase
- Hepatitis B, Hepatitis C, HIV
- Within 7 days of registration: Women of child bearing potential must have a negative serum or urine pregnancy test. See inclusion criteria 3.1.11 for WOCBP definition.
- Disease assessment: Computed tomography (CT) of the chest, CT or magnetic resonance imaging (MRI) of the abdomen and pelvis, CT or MRI of the brain. Baseline bone scan will be obtained if there is a suspicion of metastatic bone involvement.
- Tissue for correlative studies:
    - An archived tissue block or *AT LEAST* 15 unstained slides of 4 micron thickness each must be identified to submit (but not needed to be submitted) prior to registration. See Correlative Laboratory Manual (CLM) for details.
- University of Michigan only—Optional fresh tissue biopsy and whole blood sample (anytime prior to treatment on C1D1) for correlative studies

7.2 On Treatment Evaluations

7.2.1 Cycle 1 Day 1

Note: Cycle 1 Day 1 lab testing need not be repeated if completed within 7 days of starting protocol therapy.

- Physical exam
- Vital signs including weight
- ECOG performance status
- Adverse event assessment
- CBC with diff
- CMP
- Days 1-5: Guadecitabine administration
- Blood for correlative and banking studies
  - See Section 10.0 and Correlative Laboratory Manual (CMP) for details.

7.2.2 Cycle 1 and 2 only: Day 8

- Cycle 1 Day 8 only: Physical exam
- Cycle 1 Day 8 only: Vital signs including weight:
  - Before the start of infusion (between -30 minutes to 0 minutes [i.e., start of infusion])
  - During (30 minutes ±5 minutes after the start of the infusion [i.e., halfway through])
  - After the end of the infusion (60 minutes ±5 minutes)
  - If the infusion takes longer than 60 minutes, BP and pulse should follow the principles as described above or be taken more frequently, if clinically indicated.
- Cycle 1 Day 8 only: ECOG performance status
- Cycle 1 Day 8 only: ECG (single tracing) at 1 hr pre- and 0-3 hrs post dose
- Cycle 1 Day 8 only: Adverse event and concomitant medications assessment
- CBC with diff
- CMP
- Durvalumab administration
- Blood for correlative studies

7.2.3 Cycle 1 and 2 only: Day 15

- CBC with diff
- CMP

7.2.4 Cycle 1 and 2 only: Day 21

- CBC with diff

7.2.5 Cycle 2 Day 1 and subsequent cycles

- Physical exam including vital signs and weight
- ECOG Performance status
- Adverse event and concomitant medications assessment
- CBC with diff
- Comprehensive metabolic panel (CMP)
- Thyroid function: TSH, free T3, free T4 (Only If Clinically Indicated: FSH, LH, ACTH).
- Amylase and Lipase
- Tumor assessment will be done every 8 weeks starting just prior to Cycle 3.
  - Computed tomography of the chest
  - Computed tomography or magnetic resonance imaging of the abdomen and pelvis

    - Computed tomography or magnetic resonance imaging of the brain can be done at the discretion of the investigator
  - University of Michigan only—Optional fresh tissue biopsy for correlative studies, prior to Cycle 3 (after 8 weeks of study drugs)
- Days 1-5: Guadecitabine administration

7.2.6 Cycle 3 Day 8 and subsequent cycles

- CBC with diff
- CMP
- Durvalumab administration

7.3 Safety Follow-up Evaluations

A safety follow-up visit should occur when subjects permanently stop study treatment for whatever reason (toxicity, progression, or at discretion of site investigator) and should be performed 30 days (±7 days) after the last dose of treatment.

- Upon progression, patients may undergo an optional biopsy for additional correlative studies.
- Subjects who have an ongoing ≥ grade 2 or serious AE (SAE) at this visit will continue to be followed until the AE resolves to ≤ Grade 1 or baseline, is deemed clinically insignificant, and/or until a new anti-cancer treatment starts, whichever is earlier. If the patient is no longer under the clinical care of the treating investigator, follow-ups on AEs/SAEs will be done via a phone call to the patient at 60 and 90 days (± 15 business days). If the patient remains under the clinical care of the treating investigator, AE/SAE follow-up at 60 and 90 days (± 15 business days) will occur as part of clinical care.

7.4 Long Term Follow-up Evaluations

- Once disease progression is documented or have stopped protocol specified therapy due to intolerable toxicities (whichever occurs first), subjects will enter a survival follow up period every 3 months for 2 years from the time of registration.
- For patients who have stopped all protocol specified therapy, follow up will be done every 3 months via phone or other form of communication. In those patients, scans and/or labs will be per the treating investigator's discretion/standard of care and will not be dictated by protocol.
- Upon progression, patients may undergo an optional biopsy for additional correlative studies.

8. BIOSPECIMEN STUDIES AND PROCEDURES

Biospecimen-based studies will be performed at the University of Michigan.

1. **Correlate changes in CXCL9 and CXCL10 in tissue and serum as well as LINE-1 methylation in blood with clinical response to the combination therapy.**
   CXCL9 and CXCL10 will be measured in archival tissue samples by quantitative real-time PCR and immunohistochemistry, and in the serum via an ELISA assay at pre-treatment cycle 1 day 1 and on days 8 of cycles 1 and 2. Whole blood samples for DNA methylation will be collected at pre-treatment cycle 1 day 1 and on days 8 of cycles 1 and 2. Global DNA methylation will be measured by the LINE-1 methylation assay. Refer to the Correlative Laboratory Manual (CLM) for collection, labeling and shipping instructions.

2. **Correlate CD3+/CD8+ tumor infiltrating lymphocytes (TILs) and PD-L1 expression in baseline tumor tissue/TILs as well as immune cell subsets in the serum with clinical response to the combination therapy.**
   Archival tumor tissue will be analyzed by immunohistochemistry for expression of CD3+ and CD8+ immune cells as well as PD-L1 expression of the tumor and tumor infiltrating lymphocytes. We will evaluate a correlation between the degree of CD3+/CD8+ TIL/PD-L1 expression with clinical response. Immune cell subsets including antigen presenting cells (APCs), myeloid derived suppressor cells (MDSCs), and T cell subsets (Treg, Th17, Th22 and memory T cells) will be phenotypically and functionally examined in tumor tissues and blood. The immune cells will further be isolated for RNA-seq and whole genome seq analyses to identify therapy-associated immune and oncogenic signatures. Refer to the CLM for collection, labeling and shipping instructions
3. **Correlate tumor mutational burden in archived tissue with response to the combination therapy.**
   Archival tumors will undergo genetic sequencing such as but not limited to whole exome and RNA sequencing. We will correlate tumor mutational burden with clinical response. Refer to the CLM for collection, labeling and shipping instructions.
4. **Analyze and correlate changes in methylation status and correlate tumor mutation profile in fresh tissue with response to the combination therapy**
   University of Michigan subjects only, up to 8 subjects from cohort 1: Optional fresh tumor tissue biopsy will be performed at baseline and before Cycle 3 Day 1 (after approximately 8 weeks of study therapy). Up to 6 cores of accessible metastatic tumor tissue will be obtained. Site of biopsy will be determined by interventional radiologist or treating physician per standard clinical guidelines. The biopsied tissue will be submitted to the University of Michigan DNA Sequencing Core (https://brcf.medicine.umich.edu/cores/dna-sequencing/services/epigenetics/). Methylation patterns and tumor mutational burden analysis will be performed with assays including but not limited to Illumina whole exome DNA Seq, RNASeq, ATAC-Seq (Chromatin Accessibility) and Illumina Infinium Methylation EPIC BeadChip. Patients will also have whole blood drawn at baseline to provide somatic baseline DNA. We will correlate tumor mutational burden and acquired somatic mutations with clinical response. Refer to the CLM for collection and labeling instructions.

8.1 Source and Timing of Biospecimen Collections

Tissue: An archived tissue block must be identified to submit unstained slides from prior to registration. Unstained slides from an archived formalin-fixed paraffin embedded tissue block are to be submitted for each subject from prior nephrectomy or biopsy of metastatic lesion (estimated tumor content >30% of nucleated cells in specimen). Upon progression, patients may undergo an optional biopsy for additional correlative studies.

Blood: Whole blood will be collected at C1D1, C1D8, C2D8.

8.2 Storage of Biospecimens

Patient samples (tissue, blood, serum, plasma) collected for this study will be retained at the Big Ten CRC Biorepository. De-identified specimens will be stored indefinitely or until they are used up. If consent for future use of specimens is withdrawn by the subject, best efforts will be made to stop any additional studies and to destroy the specimens.

Specimens being stored long-term for potential use not outlined in the protocol are stored under Big Ten CRC guidelines

8.3 Banking of Leftover Biospecimens

Subject consent will be obtained to bank any leftover samples that were collected for study-specific correlative research. Hoosier Cancer Research Network (HCRN), as Administrative Headquarters for the Big Ten CRC, will manage the banked samples. Samples will be banked indefinitely in the Hoosier Cancer Research Network Biorepository and used for future unspecified cancer-related research.

8.4 Samples for future studies

Subject consent will be obtained for additional samples collected for future Big Ten Cancer Research Consortium studies. Hoosier Cancer Research Network, as Administrative Headquarters for the Big Ten CRC, will manage the banked samples. Samples will be banked indefinitely in the Hoosier Cancer Research Network Biorepository.

This includes:

- Whole blood: Whole blood will be collected prior to treatment on Cycle 1 Day 1.
- Pre-treatment plasma: Whole blood for plasma will be collected prior to treatment on Cycle 1 Day 1.
- Pre-treatment serum: Whole blood for serum will be collected prior to treatment on Cycle 1 Day 1.

Please refer to the Correlative Laboratory Manual (CLM) for all sample collection, processing, labeling, and shipping instructions.

8.5 Confidentiality of Biospecimens

Samples will be identified by a subject's study number assigned at the time of registration to the trial. Any material issued to collaborating researchers will be anonymized and only identified by the subject's study number.

9. CRITERIA FOR DISEASE EVALUATION

Response assessments will be made both using the Immune Related Response Criteria (irRC), and using RECIST v1.1, allowing additional comparisons among these criteria for disease response assessment. The same measurable and non-measurable lesions will be followed by both RECIST v1.1 & irRC. Disease progression will be determined using RECIST 1.1 criteria.

Definitions Associated with Response Evaluation Criteria in Solid Tumors (RECIST) version 1.1

9.1 Measurable Disease

Measurable disease is defined as the presence of at least one measurable lesion. Measurable lesions are defined as those that can be accurately measured in at least one dimension (longest diameter to be recorded) as ≥20 mm by chest x-ray, as ≥10 mm with CT scan, or ≥10 mm with calipers by clinical exam. All tumor measurements must be recorded in millimeters (or decimal fractions of centimeters).

9.1.1 Malignant Lymph Nodes

To be considered pathologically enlarged and measurable, a lymph node must be ≥15 mm in short axis when assessed by CT scan (CT scan slice thickness recommended to be no greater than 5 mm). At baseline and in follow-up, only the short axis will be measured and followed.

9.2 Non-measurable Lesions

All other lesions (or sites of disease), including small lesions (longest diameter <10 mm or pathological lymph nodes with ≥ 10 to <15 mm short axis), are considered non-measurable disease. Bone lesions, leptomeningeal disease, ascites, pleural/pericardial effusions, lymphangitis cutis/pulmonitis, inflammatory breast disease, and abdominal masses (not followed by CT or MRI), are considered as non-measurable.

NOTE: Cystic lesions that meet the criteria for radiographically defined simple cysts should not be considered as malignant lesions (neither measurable nor non-measurable) since they are, by definition, simple cysts. 'Cystic lesions' thought to represent cystic metastases can be considered as measurable lesions, if they meet the definition of measurability described above. However, if non-cystic lesions are present in the same subject, these are preferred for selection as target lesions.

9.3 Target Lesions

All measurable lesions up to a maximum of 2 lesions per organ and 5 lesions in total, representative of all involved organs, should be identified as target lesions and recorded and measured at baseline. Target lesions should be selected on the basis of their size (lesions with the longest diameter), be representative of all involved organs, but in addition should be those that lend themselves to reproducible repeated measurements. It may be the case that, on occasion, the largest lesion does not lend itself to reproducible measurement in which circumstance the next largest lesion which can be measured reproducibly should be selected. A sum of the diameters (longest for non-nodal lesions, short axis for nodal lesions) for all target lesions will be calculated and reported as the baseline sum diameters. If lymph nodes are to be included in the sum, then only the short axis is added into the sum. The baseline sum diameters will be used as reference to further characterize any objective tumor regression in the measurable dimension of the disease.

9.4 Non-target Lesions

All other lesions (or sites of disease) including any measurable lesions over and above the 5 target lesions should be identified as non-target lesions and should also be recorded at baseline. Measurements of these lesions are not required, but the presence, absence, or in rare cases unequivocal progression of each should be noted throughout follow-up.

9.5 Evaluation of Target Lesions

NOTE: In addition to the information below, also see section 4.3.2 in the international criteria proposed by the Response Evaluation Criteria in Solid Tumors (RECIST) Committee, version 1.1 (Eur J Cancer 45;2009:228-247) for special notes on the assessment of target lesions.

Complete Response (CR)	Disappearance of all target lesions. Any pathological lymph nodes (whether target or non-target) must have reduction in short axis to <10 mm.
Partial Response (PR)	At least a 30% decrease in the sum of the diameters of target lesions, taking as reference the baseline sum diameters
Progressive Disease (PD)	At least a 20% increase in the sum of the diameters of target lesions, taking as reference the smallest sum on study (this includes the baseline sum if that is the smallest on study). In addition to the relative increase of 20%, the sum must also

	demonstrate an absolute increase of at least 5 mm. (Note: the appearance of one or more new lesions is also considered progressions).
Stable Disease (SD)	Neither sufficient shrinkage to qualify for PR nor sufficient increase to qualify for PD, taking as reference the smallest sum diameters while on study

9.6 Evaluation of Non-Target Lesions

Complete Response (CR)	Disappearance of all non-target lesions and normalization of tumor marker level. All lymph nodes must be non-pathological in size (<10 mm short axis)<br><br>Note: If tumor markers are initially above the upper normal limit, they must normalize for a subject to be considered in complete clinical response.
Non-CR/ Non-PD	Persistence of one or more non-target lesion(s) and/or maintenance of tumor marker level above the normal limits
Progressive Disease (PD)	Appearance of one or more new lesions and/or unequivocal progression of existing non-target lesions. Unequivocal progression should not normally trump target lesion status. It must be representative of overall disease status change, not a single lesion increase.

Although a clear progression of "non-target" lesions only is exceptional, the opinion of the site investigator should prevail in such circumstances, and the progression status should be confirmed at a later time by the sponsor investigator.

9.7 Evaluation of Best Overall Response

The best overall response is the best response recorded from the start of the treatment until disease progression/recurrence (taking as reference for PD the smallest measurements recorded since the treatment started). In general, the subject's best response assignment will depend on the achievement of both measurement and confirmation criteria.

Target Lesions	Non-Target Lesions	New Lesions	Overall Response**
CR	CR	No	CR
CR	Non-CR/ Non-PD	No	PR
CR	Not evaluated	No	PR
PR	Non-PD/ or not all evaluated	No	PR

SD	Non-PD or not all evaluated	No	SD
Not all evaluated	Non-PD	No	Non-evaluable
PD	Any	Yes or No	PD
Any	PD*	Yes or No	PD
Any	Any	Yes	PD
*In exceptional circumstances, unequivocal progression in non-target lesions may be accepted as disease progression.<br>**Progression or response must be confirmed with a follow-up scan performed at a minimum of 4 weeks later			

Subjects with a global deterioration of health status requiring discontinuation of treatment without objective evidence of disease progression at that time should be classified as having "symptomatic deterioration". Every effort should be made to document the objective progression even after discontinuation of treatment.

In some circumstances it may be difficult to distinguish residual disease from normal tissue. When the evaluation of complete response depends on this determination, it is recommended that the residual lesion be investigated (fine needle aspirate/biopsy) to confirm the complete response status.

9.8 Definitions for Response Evaluation – RECIST 1.1

9.8.1 First Documentation of Response

The time between initiation of therapy and first documentation of PR or CR.

9.8.2 Confirmation of Response

To be assigned a status of complete or partial response, changes in tumor measurements must be confirmed by repeat assessments performed no less than four weeks after the criteria for response are first met.

9.8.3 Duration of Response

Duration of overall response—the period measured from the time that measurement criteria are met for complete or partial response (whichever status is recorded first) until the date that recurrent or progressive disease is objectively documented (taking as reference for progressive disease the smallest measurements recorded since treatment started).

9.8.4 Duration of Overall Complete Response

The period measured from the time that measurement criteria are met for complete response until the first date that recurrent disease is objectively documented.

9.8.5 Objective Response Rate

The objective response rate is the proportion of all subjects with confirmed PR or CR according to RECIST 1.1, from the start of treatment until disease progression/recurrence (taking as reference for progressive disease the smallest measurements recorded since the start of treatment).

9.8.6 Disease Control Rate:

The disease control rate is the proportion of all subjects with stable disease (SD) for 8 weeks, or partial response (PR), or complete response (CR) according to RECIST 1.1, from the start of treatment until disease progression/recurrence (taking as reference for progressive disease the smallest measurements recorded since the start of treatment).

9.8.7 Time to Progression:

A measurement from the start of treatment until the criteria for disease progression is met as defined by RECIST 1.1. Subjects who have not progressed or have died due to any cause will be right-censored at the date of the last disease evaluation or date of death.

9.8.8 Progression Free Survival

A measurement from the start of treatment until the criteria for disease progression is met as defined by RECIST 1.1 or death occurs. Subjects who have not progressed will be right-censored at the date of the last disease evaluation.

9.8.9 Overall Survival

Overall survival is defined by the start of treatment to date of death from any cause. Patients alive at last time of contact will be right-censored.

9.9 Immune Related Response Criteria:

This study will evaluate concordance of the Immune Related Response Criteria (irRC) with RECIST 1.1. These response criteria were developed to overcome the variable and unusual patterns of response to immunotherapeutic agents, in particular, ipilimumab. The development of the guidelines was prompted by observations, mostly in subjects with metastatic melanoma, of initial disease progression followed by later response, late responses, and mixed responses with an overall decrease in tumor burden.

9.9.1 Antitumor response based on total measurable tumor burden

For the irRC, only index and measurable new lesions are taken into account (in contrast to conventional WHO criteria, which do not require the measurement of new lesions, nor do they include new lesion measurements in the characterization of evolving tumor burden). At the baseline tumor assessment, the sum of the products of the two largest perpendicular diameters (SPD) of all index lesions (5 lesions per organ, up to 10 visceral lesions) is calculated. At each subsequent tumor assessment, the SPD of the index lesions and of new, measurable lesions (up to 5 new lesions per organ; 10 visceral lesions) are added together to provide the total tumor burden:

$$\text{Tumor Burden} = SPD_{\text{index lesions}} + SPD_{\text{new, measurable lesions}}$$

Table: Comparison of WHO and irRC criteria

	WHO	irRC
New, measurable lesions	Always represent PD	Incorporated into tumor burden
New, non measurable lesions	Always represent PD	Do not define progression (but preclude irCR)

Non-index lesions	Changes contribute to defining BOR of CR, PR, SD, and PD	Contribute to defining irCR (complete disappearance required)
CR	Disappearance of all lesions in two consecutive observations not less than 4 wk apart	Disappearance of all lesions in two consecutive observations not less than 4 wk apart
PR	≥50% decrease in SPD of all index lesions compared with baseline in two observations at least 4 wk apart, in absence of new lesions or unequivocal progression of non-index lesions	≥50% decrease in tumor burden compared with baseline in two observations at least 4 wk apart
SD	50% decrease in SPD compared with baseline cannot be established nor 25% increase compared with nadir, in absence of new lesions or unequivocal progression of non-index lesions	50% decrease in tumor burden compared with baseline cannot be established nor 25% increase compared with nadir
PD	At least 25% increase in SPD compared with nadir and/or unequivocal progression of non-index lesions and/or appearance of new lesions (at any single time point)	At least 25% increase in tumor burden compared with nadir (at any single time point) in two consecutive observations at least 4 wk apart

9.9.2 Time-point response assessment using irRC

Percentage changes in tumor burden per assessment time point describe the size and growth kinetics of both conventional and new, measurable lesions as they appear. At each tumor assessment, the response in index and new, measurable lesions is defined based on the change in tumor burden (after ruling out irPD). Decreases in tumor burden must be assessed relative to baseline measurements (i.e., the SPD of all index lesions at screening). The irRC were derived from WHO criteria and, therefore, the thresholds of response remain the same. However, the irRC response categories have been modified from those of WHO criteria as detailed in the above table.

9.9.3 Overall response using the irRC

The sum of the products of diameters at tumor assessment using the immune-related response criteria (irRC) for progressive disease incorporates the contribution of new measurable lesions. Each net Percentage Change in Tumor Burden per assessment using irRC criteria accounts for the size and growth kinetics of both old and new lesions as they appear.

Definition of Index Lesions Response Using irRC

- **irComplete Response (irCR)**: Complete disappearance of all *index* lesions. This category encompasses exactly the same subjects as “CR” by the mWHO criteria.
- **irPartial Response (irPR)**: Decrease, relative to baseline, of 50% or greater in the sum of the products of the 2 largest perpendicular diameters of all *index* and all new measurable lesions (i.e., Percentage Change in Tumor Burden). Note: the appearance of new measurable
- **Disease (irSD)**: Does not meet criteria for irCR or irPR, in the absence of progressive disease.
- **irProgressive Disease (irPD)**: At least 25% increase Percentage Change in Tumor Burden (i.e., taking sum of the products of all *index* lesions and any new lesions) when compared to SPD at nadir.

Definition of Non-Index Lesions Response Using irRC

- irComplete Response (irCR): Complete disappearance of all *non-index* lesions. This category encompasses exactly the same subjects as "CR" by the mWHO criteria.
- irPartial Response (irPR) or irStable Disease (irSD): *non-index* lesion(s) are not considered in the definition of PR, these terms do not apply.
- irProgressive Disease (irPD): Increases in number or size of *non-index* lesion(s) does not constitute progressive disease unless/until the Percentage Change in Tumor Burden increases by 25% (i.e., the SPD at nadir of the index lesions increases by the required amount).

Impact of New Lesions on irRC New lesions in and by themselves do not qualify as progressive disease. However, their contribution to total tumor burden is included in the SPD, which in turn feeds into the irRC criteria for tumor response. Therefore, new non-measurable lesions will not discontinue any subject from the study.

9.9.4 Definition of Overall Response Using irRC

Overall response using irRC will be based on these criteria (see Table below):

- Immune-Related Complete Response (irCR): Complete disappearance of *all* tumor lesions (index and non-index together with no new measurable/unmeasurable lesions) for at least 4 weeks from the date of documentation of complete response.
- Immune-Related Partial Response (irPR): The sum of the products of the two largest perpendicular diameters of all index lesions is measured and captured as the SPD baseline. At each subsequent tumor assessment, the sum of the products of the two largest perpendicular diameters of all index lesions and of new measurable lesions are added together to provide the Immune Response Sum of Product Diameters (irSPD). A decrease, relative to baseline of the irSPD compared to the previous SPD baseline, of 50% or greater is considered an immune Partial Response (irPR).
- Immune-Related Stable Disease (irSD): irSD is defined as the failure to meet criteria for immune complete response or immune partial response, in the absence of progressive disease.
- Immune-Related Progressive Disease (irPD): It is recommended in difficult cases to confirm PD by serial imaging. Any of the following will constitute progressive disease:
    - o At least 25% increase in the sum of the products of all index lesions over nadir SPD calculated for the index lesions.
    - o At least a 25% increase in the sum of the products of all index lesions and new measurable lesions (irSPD) over the baseline SPD calculated for the index lesions.
- Immune-Related Best Overall Response Using irRC (irBOR) irBOR is the best confirmed irRC overall response over the study as a whole, recorded between the date of first dose until the last tumor assessment before subsequent therapy (except for local palliative radiotherapy for painful bone lesions) for the individual subject in the study. For the assessment of irBOR, all available assessments per subject are considered. irCR or irPR determinations included in the irBOR assessment must be confirmed by a second (confirmatory) evaluation meeting the criteria for response and performed no less than 4 weeks after the criteria for response are first met.

Derivation of irRC overall responses

Measurable Response	Nonmeasurable Response	Overall Response

Index and new, measurable lesions (tumor burden),*%	Non-index lesions	New, Nonmeasurable lesions	Using irRC
↓100	Absent	Absent	irCR†
↓100	Stable	Any	irPR†
↓100	Unequivocal Progression	Any	irPR†
↓≥50	Any/Stable	Any	irPR†
↓≥50	Unequivocal Progression	Any	irPR†
↓<50 to <25↑	Any/Stable	Any	irSD
↓<50 to <25↑	Unequivocal Progression	Any	irSD
≥25	Any	Any	irPD†
*Decreases assessed relative to baseline (scan prior to start of any protocol therapy), including measurable lesions only.<br>†Assuming response (irCR) and progression (irPD) are confirmed by a second, consecutive assessment at least 4 wk apart.			

10. DRUG INFORMATION

10.1 Durvalumab

Durvalumab is a human monoclonal antibody of the immunoglobulin G1 kappa (IgG1κ) subclass that inhibits binding of programmed cell death ligand 1 (PD-L1) to programmed cell death 1 (PD-1) and CD80 (B7-1).

10.1.1 Supplier/How Supplied

The Investigational Products Supply section of AstraZeneca/MedImmune will supply durvalumab to the site investigator as a 500-mg vial solution for infusion after dilution.

AstraZeneca/MedImmune will supply durvalumab at no charge to subjects participating in this clinical trial.

The investigator shall take responsibility for and shall take all steps to maintain appropriate records and ensure appropriate supply, storage, handling, distribution, and usage of investigational product in accordance with the protocol and any applicable laws and regulations.

10.1.2 Preparation

Durvalumab solution contains 50mg/mL durvalumab (MEDI4736), 26 mM histidine/histidine hydrochloride, 275 mM trehalose dihydrate, and 0.02% weight/volume (w/v) polysorbate 80; it has a pH of 6.0. The nominal fill volume is 10.0 mL. Investigational product vials are stored at 2°C to 8°C (36°F to 46°F) and must not be frozen.

The dose of durvalumab (MEDI4736) for administration must be prepared by the Investigator’s or site’s designated IP manager using aseptic technique. Total time from needle puncture of the durvalumab (MEDI4736) vial to the start of administration should not exceed:

- 24 hours at 2°C to 8°C (36°F to 46°F)
- 4 hours at room temperature

Infusion solution must be allowed to equilibrate to room temperature prior to commencement of administration.

A dose of 1500mg will be administered using an IV bag containing 0.9% (w/v) saline or 5% (w/v) dextrose, with a final durvalumab (MEDI4736) concentration ranging from 1 to 20 mg/mL, and delivered through an IV administration set with a 0.2- or 0.22-µm in-line filter. Add 30.0 mL of durvalumab (MEDI4736) (i.e., 1500mg of durvalumab [MEDI4736]) to the IV bag. The IV bag size should be selected such that the final concentration is within 1 to 20 mg/mL. Mix the bag by gently inverting to ensure homogeneity of the dose in the bag.

Standard infusion time is 60 minutes (± 5 minutes). Less than 55 minutes is considered a deviation. In the event that there are interruptions during infusion, the total allowed time should not exceed 8 hours at room temperature.

The IV line will be flushed with a volume of IV diluent equal to the priming volume of the infusion set used after the contents of the IV bag are fully administered, or complete the infusion according to institutional policy to ensure the full dose is administered and document if the line was not flushed. Durvalumab solution should not be infused with other solutions or medications.

In the event that there are interruptions during infusion, the total allowed time should not exceed 8 hours at room temperature. If either preparation time or infusion time exceeds the time limits a new dose must be prepared from new vials. Durvalumab (MEDI4736) does not contain preservatives, and any unused portion must be discarded.

No incompatibilities between durvalumab and polyvinylchloride or polyolefin IV bags have been observed.

10.1.3 Storage and Stability

Unopened vials of Durvalumab Drug Product must be stored at 2ºC to 8 ºC (36ºF to 46 ºF). Drug product should be kept in secondary packaging until use to prevent excessive light exposure.

10.1.4 Handling and Disposal

Please refer to the current version of the Investigator's Brochure (IB) for additional information regarding this drug

10.1.5 Dispensing

Durvalumab must be dispensed only from official study sites and to eligible subjects under the supervision of the site investigator. Durvalumab be stored in a secure area according to local regulations. It is the responsibility of the site investigator to ensure that study drug is only dispensed to subjects.

10.1.6 Adverse Events

The identified risks with durvalumab monotherapy include the following: cough/productive cough, pneumonitis, ILD, dysphonia, ALT/AST increased, hepatitis, diarrhea, abdominal pain, colitis, hypothyroidism, hyperthyroidism, blood TSH increased, blood TSH decreased, adrenal insufficiency, hypophysitis/hypopituitarism, type 1 diabetes mellitus, diabetes insipidus, blood creatinine increased,

dysuria, nephritis, rash, pruritus, night sweats, dermatitis myocarditis, pyrexia, peripheral edema, upper respiratory tract infections, pneumonia, oral candidiasis, dental and oral soft tissue infections, influenza, myalgia, myositis, polymyositis and infusion related reaction.

The following events have been seen with other checkpoint inhibitors (Naidoo et al 2015, Champiat et al 2016) and/or may possibly occur due to the mechanism of action of the PD-1/PD-L1 class or mAb therapeutics in general.

- Potential imAEs including:
  - Pancreatitis
  - Other rare or less frequent events with a potential immune-mediated aetiology, eg, pericarditis, sarcoidosis, uveitis, and other events involving the eye (eg, keratitis and optic neuritis), skin (eg, scleroderma and vitiligo), and haematological (eg, haemolytic anaemia and immune thrombocytopenic purpura), rheumatological events (polymyalgia rheumatic and autoimmune arthritis), neuropathy/neuromuscular toxicities (eg, myasthenia gravis, Guillain Barre syndrome), vasculitis, non-infectious meningitis and non-infectious encephalitis.
- Hypersensitivity reactions including:
  - Anaphylaxis and allergic reaction
  - Cytokine release syndrome
  - Immune complex disease
- Other infections

Please refer to the current version of the Investigator's Brochure (IB) for additional information regarding this drug.

10.2 Guadecitabine

Guadecitabine is a potent inhibitor of DNA methylation that has been shown to induce a dose-dependent decrease of global DNA and gene-specific methylation in many different human cancer cell lines.

10.2.2 Supplier/How Supplied

Guadecitabine will be supplied by the manufacturer, Astex, at no charge to subjects participating in this clinical trial.

10.2.3 Preparation

Guadecitabine is available as a two-vial system, referred to as (1) SGI-110 for Injection, 100mg and (2) SGI-110 Diluent for Reconstitution, available in two configurations of the same formulation but different fill volumes (1.2mL and 3mL):

- SGI-110 for Injection, 100mg contains guadecitabine, 100mg of free acid equivalent as a dry lyophilized solid
- SGI-110 Diluent for Reconstitution, 1.2mL or 3mL of a non-aqueous diluent for reconstitution

The diluent is comprised of 3 commonly used excipients, propylene glycol, glycerin and ethanol. Guadecitabine solution is reconstituted at a maximum concentration of 100mg/mL for SC administration.

Both product and diluent are contained in clear glass vials with a latex-free rubber stopper and capped with an aluminum flip-off seal.

10.2.4 Storage and Stability

SGI-110 for Injection, 100mg vial is stored at 2ºC to 8 ºC in the original packaging in an upright position until use. SGI-110 Diluent for Reconstitution, 1.2mL is between 2ºC and 8 ºC and 3 mL is between 2ºC to 30 ºC in an upright position until use. Both vials are preservative free and for single use only.

10.2.5 Handling and Disposal

Occupational Safety and Health Administration (OSHA) Guidelines for handling cytotoxic drugs outlined in the American Journal of Hospital Pharmacy should be followed. As with other potentially toxic anti-cancer agents, care should be exercised in the handling and preparation of guadecitabine. The use of gloves and protective garments is recommended. Preparation should occur in a vertical laminar flow biological hood using proper aseptic technique.

10.2.6 Dispensing

Guadecitabine must be dispensed only from official study sites and to eligible subjects under the supervision of the site investigator. Guadecitabine be stored in a secure area according to local regulations. It is the responsibility of the site investigator to ensure that study drug is only dispensed to subjects.

10.2.7 Adverse Events

Please refer to the current version of the Investigator's Brochure (IB) for additional information regarding this drug

11. ADVERSE EVENTS

11.1 Definitions

11.1.1 Adverse Event (AE)

The International Conference on Harmonization (ICH) Guideline for Good Clinical Practice (GCP) E6(R1) defines an AE as:

Any untoward medical occurrence in a patient or clinical investigation subject administered a pharmaceutical product and which does not necessarily have a causal relationship with this treatment. An AE can therefore be any unfavorable and unintended sign (including an abnormal laboratory finding), symptom, or disease temporally associated with the use of a medicinal product, whether or not considered related to the medicinal product.

An AE includes but is not limited to any clinically significant worsening of a subject's pre-existing condition. An abnormal laboratory finding (including ECG finding) that requires an action or intervention by the investigator, or a finding judged by the investigator to represent a change beyond the range of normal physiologic fluctuation, should be reported as an AE.

Adverse events may be treatment emergent (i.e., occurring after initial receipt of investigational product) or nontreatment emergent. A nontreatment-emergent AE is any new sign or symptom, disease,

or other untoward medical event that begins after written informed consent has been obtained but before the subject has received investigational product.

Elective treatment or surgery or preplanned treatment or surgery (that was scheduled prior to the subject being enrolled into the study) for a documented pre-existing condition, that did not worsen from baseline, is not considered an AE (serious or non-serious). An untoward medical event occurring during the prescheduled elective procedure or routinely scheduled treatment should be recorded as an AE or SAE.

The term AE is used to include both serious and non-serious AEs.

Disease progression should not be recorded as an AE, unless it is attributable to the study regimen by the site investigator.

11.1.2 Serious Adverse Event (SAE)

An SAE is an adverse event that:

- Results in death. **NOTE**: Death due to disease progression should not be reported as a SAE, unless it is attributable by the site investigator to the study drug(s)
- Is life-threatening (defined as an event in which the subject was at risk of death at the time of the event; it does not refer to an event which hypothetically might have caused death if it were more severe)
- Requires inpatient hospitalization for >24 hours or prolongation of existing hospitalization. **NOTE:** Hospitalization for anticipated or protocol specified procedures such as administration of chemotherapy, central line insertion, metastasis interventional therapy, resection of primary tumor, or elective surgery, will not be considered serious adverse events.
- Results in persistent or significant disability/incapacity
- Is a congenital anomaly or birth defect
- Is an important medical event (defined as a medical event(s) that may not be immediately life-threatening or result in death or hospitalization but, based upon appropriate medical and scientific judgment, may jeopardize the subject or may require intervention (e.g., medical, surgical) to prevent one of the other serious outcomes listed in the definition above). Examples of such events include, but are not limited to, intensive treatment in an emergency room or at home for allergic bronchospasm; blood dyscrasias or convulsions not resulting in hospitalization; or the development of drug dependency or drug abuse.

11.1.3 Definition of Immune-Mediated Adverse Events (imAEs)

Immune-mediated AEs are adverse events associated with the treatment of patients with immunotherapy treatments that appear to be associated with the immune therapy's mechanism of action. Based on these potential imAEs, AstraZeneca/Medimmune has defined a list of specific adverse event terms (see AESIs below) that are selected adverse events that **must be reported to Big Ten Cancer Research Consortium Administrative Headquarters (Big Ten CRC AHQ) within 24 hours** from the time the site investigator is aware of such an occurrence, regardless of whether the site investigator considers the event to be related to study drug(s). See Section 11.2 for reporting criteria.

11.1.4 Definition of Adverse Events of Special Interest (AESI)

An adverse event of special interest (AESI) is one of scientific and medical interest specific to understanding of the Investigational Product and may require close monitoring and rapid communication by the local investigator to sponsor-investigator via the Big Ten CRC project manager. An AESI may be serious or non-serious. The rapid reporting of AESIs allows ongoing surveillance of these events to characterize and understand them in association with the use of this investigational product.

AESIs for durvalumab include but are not limited to events with a potential inflammatory or immune-mediated mechanism and which may require more frequent monitoring and/or interventions such as steroids, immunosuppressants and/or hormone replacement therapy. These AESIs are being closely monitored in clinical studies with durvalumab monotherapy and combination therapy. An immune-mediated adverse event (imAE) is defined as an adverse event that is associated with drug exposure and is consistent with an immune-mediated mechanism of action and where there is no clear alternate etiology. Serologic, immunologic, and histologic (biopsy) data, as appropriate, should be used to support an imAE diagnosis. Appropriate efforts should be made to rule out neoplastic, infectious, metabolic, toxin, or other etiologic causes of the imAE.

If the Investigator has any questions in regards to an adverse event (AE) being an imAE, the Investigator should promptly contact the sponsor-investigator via the Big Ten CRC project manager.

AESIs observed with durvalumab include:

- Diarrhea / Colitis and intestinal perforation
- Pneumonitis / ILD
- hepatitis / transaminase increases
- Endocrinopathies (i.e. events of hypophysitis/hypopituitarism, adrenal insufficiency, hyper- and hypothyroidism and type I diabetes mellitus)
- Rash / Dermatitis
- Nephritis / Blood creatinine increases
- Pancreatitis / serum lipase and amylase increases
- Myocarditis
- Myositis / Polymyositis
- Neuropathy / neuromuscular toxicity (e.g. Guillain-Barré, and myasthenia gravis)
- Other inflammatory responses that are rare / less frequent with a potential immune-mediated aetiology include, but are not limited to, pericarditis, sarcoidosis, uveitis and other events involving the eyeskin, haematological and rheumatological events, vasculitis, non-infectious meningitis and non-infectious encephalitis.

In addition, infusion-related reactions and hypersensitivity/anaphylactic reactions with a different underlying pharmacological aetiology are also considered AESIs.

Further information on these risks (e.g. presenting symptoms) can be found in the current version of the durvalumab Investigator's Brochure. More specific guidelines for their evaluation and treatment are described in detail in the Dosing Modification and Toxicity Management Guidelines (please see Section 6.2). These guidelines have been prepared to assist the site Investigator in the exercise of his/her clinical

judgment in treating these types of toxicities. These guidelines apply to AEs considered causally related to the study drug/study regimen by the reporting investigator.

11.1.4.1 Pneumonitis

Adverse events of pneumonitis are of interest for AstraZeneca/Medimmune, as pneumonitis has been reported with anti-PD-1 MAs [13] [14]Initial work-up should include high-resolution CT scan, ruling out infection, and pulse oximetry. Pulmonary consultation is highly recommended.

Guidelines for the management of subjects with immune-mediated adverse events including pneumonitis are outlined in Appendix I.

11.1.4.2 Hypersensitivity Reactions

Hypersensitivity reactions as well as infusion-related reactions have been reported with anti-PD-L1 and anti-PD-1 therapy [14]. As with the administration of any foreign protein and/or other biologic agents, reactions following the infusion of MAs can be caused by various mechanisms, including acute anaphylactic (immunoglobulin E-mediated) and anaphylactoid reactions against the MAb, and serum sickness. Acute allergic reactions may occur, may be severe, and may result in death. Acute allergic reactions may include hypotension, dyspnea, cyanosis, respiratory failure, urticaria, pruritus, angioedema, hypotonia, arthralgia, bronchospasm, wheeze, cough, dizziness, fatigue, headache, hypertension, myalgia, vomiting, and unresponsiveness.

Guidelines for management of subjects with hypersensitivity (including anaphylactic reaction) and infusion-related reactions are outlined in Appendix I.

11.1.4.3 Hepatic function abnormalities (hepatotoxicity)

Increased transaminases have been reported during treatment with anti-PD-L1/anti-PD-1 antibodies [14]. Inflammatory hepatitis has been reported in 3% to 9% of subjects treated with anti-CTLA-4 monoclonal antibodies (e.g., ipilimumab). The clinical manifestations of ipilimumab-treated subjects included general weakness, fatigue, nausea, and/or mild fever and increased liver function tests such as AST, ALT, alkaline phosphatase, and/or total bilirubin.

Hepatic function abnormality is defined as any increase in ALT or AST to greater than 3 × ULN and concurrent increase in total bilirubin to be greater than 2 × ULN. Concurrent findings are those that derive from a single blood draw or from separate blood draws taken within 8 days of each other. Follow-up investigations and inquiries will be initiated promptly by the investigational site to determine whether the findings are reproducible and/or whether there is objective evidence that clearly supports causation by a disease (e.g., cholelithiasis and bile duct obstruction with distended gallbladder) or an agent other than the investigational product. Guidelines for management of subjects with hepatic function abnormality are outlined in Appendix I.

Cases where a subject shows an AST **or** ALT ≥3xULN **or** total bilirubin ≥ 2xULN may need to be reported as SAEs, these cases should be reported as SAEs if, after evaluation, they meet the criteria for a Hy’s Law case or if any of the individual liver test parameters fulfill any of the SAE criteria.

<u>Criteria for Hy’s Law (FDA Guidance 2009)</u>

- The drug causes hepatocellular injury, generally shown by a higher incidence of 3-fold or greater elevations above the ULN of ALT or AST than the (non-hepatotoxic) control drug or placebo

- Among trial subjects showing such aminotransferase elevations, often with aminotransferases much greater than 3 x ULN, one or more also show elevation of serum total bilirubin to >2 x ULN, without initial findings of cholestasis (elevated serum alkaline phosphatase)
- No other reason can be found to explain the combination of increased aminotransferases and total bilirubin, such as viral hepatitis A, B, or C; pre-existing or acute liver disease; or another drug capable of causing the observed injury.

11.1.4.4 Gastrointestinal disorders

Guidelines on management of diarrhea and colitis in patients receiving durvalumab are provided in Appendix I.

11.1.4.5 Endocrine disorders

Immune-mediated endocrinopathies include hypophysitis, adrenal insufficiency, and hyper- and hypothyroidism. Guidelines for the management of patients with immune-mediated endocrine events are provided in Appendix I.

11.1.4.6 Pancreatic disorders

Immune-mediated pancreatitis includes autoimmune pancreatitis, and lipase and amylase elevation. Guidelines for the management of patients with immune-mediated pancreatic disorders are provided in Appendix I.

11.1.4.7 Neurotoxicity

Immune-mediated nervous system events include encephalitis, peripheral motor and sensory neuropathies, Guillain-Barré, and myasthenia gravis. Guidelines for the management of patients with immune-mediated neurotoxic events are provided in Appendix I.

11.1.4.8 Nephritis

Consult with Nephrologist. Monitor for signs and symptoms that may be related to changes in renal function (e.g. routine urinalysis, elevated serum BUN and creatinine, decreased creatinine clearance, electrolyte imbalance, decrease in urine output, proteinuria, etc.).

Patients should be thoroughly evaluated to rule out any alternative etiology (e.g., disease progression, infections etc.).

Steroids should be considered in the absence of clear alternative etiology even for low grade events (Grade 2), to prevent potential progression to higher grade event. Guidelines for the management of patients with immune-mediated neurotoxic events are provided in Appendix I.

11.1.5 Unexpected Adverse Event

For this study, an AE is considered unexpected when it varies in nature, intensity or frequency from information provided in the current IB, package insert, or when it is not included in the informed consent document as a potential risk. Unexpected also refers to AEs that are mentioned in the IB as occurring with a class of drugs or are anticipated from the pharmacological properties of the drug but are not specifically mentioned as occurring with the particular drug under investigation.

11.1.6 Relatedness

AEs will be categorized according to the likelihood that they are related to the study drug(s). Specifically, they will be categorized using the following terms:

**Unrelated**	The Adverse Event is ***not related*** to the drug(s)
**Unlikely**	The Adverse Event is ***doubtfully related*** to the drug(s)
**Possible**	The Adverse Event ***may be related*** to the drug(s)
**Probable**	The Adverse Event is ***likely related*** to the drug(s)
**Definite**	The Adverse Event is ***clearly related*** to the drug(s)

11.2 Reporting

11.2.1 Adverse Events

- AEs will be recorded from time of signed informed consent until 90 days after discontinuation of study drug(s) .
- AEs will be recorded regardless of whether or not they are considered related to the study drug(s).
- All AEs will be recorded in the subject's medical record and on the appropriate study specific eCRF form within the EDC.
- All AEs considered related to study drug(s) will be followed until resolution to ≤ Grade 1 or baseline, deemed clinically insignificant, and/or until a new anti-cancer treatment starts, whichever occurs first.
- Adverse events will be recorded in the EDC using a recognized medical term or diagnosis that accurately reflects the event. Adverse events will be assessed by the investigator for severity, relationship to the investigational product, possible etiologies, and whether the event meets criteria of an SAE and therefore requires immediate notification to AstraZeneca/MedImmune Patient Safety.
- The following variables will be collected for each AE: In addition, the following variables will be collected for SAEs as applicable:
    - AE (verbatim)
    - The date when the AE started and stopped
    - The maximum CTCAE grade reported
    - Changes in CTCAE grade
    - Whether the AE is serious or not
    - Investigator causality rating against the IPs (yes or no)
    - Action taken with regard to IPs
    - Administration of treatment for the AE
    - Outcome

      In addition, the following variables will be collected for SAEs:
        - Date the AE met criteria for SAE
        - Date the Investigator became aware of the SAE
        - Seriousness criteria fulfilled
        - Date of hospitalization

- Date of discharge
- Probable cause of death
- Date of death
- Whether an autopsy was performed
- Causality assessment in relation to study procedure(s)
- Causality assessment in relation to other medication
- Description of the SAE

The grading scales found in the revised NCI CTCAE version 4 will be utilized for all events with an assigned CTCAE grading. For those events without assigned CTCAE grades, the recommendation in the CTCAE criteria that converts mild, moderate, and severe events into CTCAE grades should be used. A copy of the CTCAE version 4 can be downloaded from the Cancer Therapy Evaluation Program website (http://ctep.cancer.gov).

Adverse events and serious adverse events will be recorded from time of signature of informed consent, throughout the treatment period and including the follow-up period (90 days after the last dose of durvalumab.

During the course of the study all AEs and SAEs should be proactively followed up for each subject. Every effort should be made to obtain a resolution for all events, even if the events continue after discontinuation/study completion.

If a subject discontinues from treatment for reasons other than disease progression, and therefore continues to have tumor assessments, drug or procedure-related SAEs must be captured until the patient is considered to have confirmed PD and will have no further tumor assessments.

The investigator is responsible for following all SAEs until resolution, until the subject returns to baseline status, or until the condition has stabilized with the expectation that it will remain chronic, even if this extends beyond study participation.

11.2.2 Serious Adverse Events (SAEs)

11.2.2.1 Site Requirements for Reporting SAEs to Big Ten CRC Administrative Headquarters

- SAEs will be reported from time of signed informed consent until 90 days after discontinuation of study drug(s) .
- SAEs will be reported on the SAE Submission Form and entered in the SAE tab in the EDC **within 1 business day** of discovery of the event.
- SAEs include events related and unrelated to the study drug(s).
- All SAEs will be recorded in the subject’s medical record and on the appropriate study specific eCRF form within the EDC.
- All SAEs regardless of relation to study drug will be followed until resolution to ≤ Grade 1 or baseline and/or deemed clinically insignificant and/or until a new anti-cancer treatment starts, whichever occurs first.

The site will submit the completed SAE Submission Form (see Documents/Info tab of the EDC) to Big Ten CRC AHQ within **1 business day** of discovery of the event. The form may be sent electronically to safety@hoosiercancer.org.

The site investigator is responsible for informing the IRB and/or other local regulatory bodies of the SAE as per local requirements.

The original copy of the SAE Submission Form and the email correspondence must be kept within the study file at the study site.

During the study, all SAEs should be proactively followed up for each subject. Every effort should be made to obtain a resolution for all events. Once the SAE has resolved, sites must electronically submit a follow up SAE Submission Form within a reasonable timeframe to Big Ten CRC AHQ at safety@hoosiercancer.org.

11.2.2.2 Other events requiring immediate reporting

Requirements for Reporting Overdose
An overdose is defined as a subject receiving a dose of durvalumab in excess of that specified in the Investigator's Brochure, unless otherwise specified in this protocol.

Any overdose of a study subject with durvalumab, with or without associated AEs/SAEs, is required to be reported to Big Ten CRC AHQ **within 1 business day** of knowledge of the event. Big Ten CRC AHQ will report the event to AstraZeneca/MedImmune Patient Safety or designee within 24 hours using the designated Safety e-mailbox (AEMailboxClinicalTrialTCS@astrazeneca.com). If the overdose results in an AE, the AE must also be recorded as an AE. Overdose does not automatically make an AE serious, but if the consequences of the overdose are serious, for example death or hospitalization, the event is serious and must be recorded and reported as an SAE. There is currently no specific treatment in the event of an overdose of durvalumab.

The investigator will use clinical judgment to treat any overdose.

Requirements for Reporting Hepatic function abnormality
Hepatic function abnormality (as defined in Section 11.1.4.3) in a study subject, with or without associated clinical manifestations, is required to be reported as "hepatic function abnormal" ***within 24 hours of knowledge of the event*** to Big Ten CRC AHQ, unless a definitive underlying diagnosis for the abnormality (e.g., cholelithiasis or bile duct obstruction) that is unrelated to investigational product has been confirmed. Big Ten CRC AHQ will report the event to AstraZeneca/MedImmune Patient Safety or designee within 24 hours using the designated Safety e-mailbox.

- If the definitive underlying diagnosis for the abnormality has been established and is unrelated to investigational product, the decision to continue dosing of the study subject will be based on the clinical judgment of the site investigator.
- If no definitive underlying diagnosis for the abnormality is established, dosing of the study subject must be interrupted immediately. Follow-up investigations and inquiries must be initiated by the investigational site without delay.

Each reported event of hepatic function abnormality will be followed by the site investigator and evaluated by the sponsor-investigator and AstraZeneca/MedImmune.

Requirements for Reporting Pregnancy

If a subject becomes pregnant during the study, durvalumab should be discontinued immediately. Pregnancy itself, or pregnancy of a subject's partner, is not regarded as an adverse event unless there is a suspicion that the investigational product under study may have interfered with the effectiveness of a contraceptive medication. Congenital abnormalities/birth defects and spontaneous miscarriages should be reported and handled as SAEs. Elective abortions without complications should not be handled as AEs. The outcome of any conception occurring from the date of the first dose until 90 days after the last dose (spontaneous miscarriage, elective termination, ectopic pregnancy, normal birth, or congenital abnormality) should be followed up and documented even if the subject was withdrawn from the study drug.

Pregnancy in a female subject who has received investigational product is required to be reported ***within 1 business day of knowledge of the event*** to Big Ten CRC AHQ on the Pregnancy Report form (See Documents/Info tab of the EDC). Big Ten CRC AHQ will report the event to AstraZeneca/MedImmune Patient Safety or designee within 1 business day using the designated Safety e-mailbox (AEMailboxClinicalTrialTCS@astrazeneca.com).

Subjects who become pregnant during the study period must not receive additional doses of investigational product but will not be withdrawn from the study. The pregnancy will be followed for outcome of the mother and child (including any premature terminations) and should be reported to Big Ten CRC AHQ after outcome is known.

Big Ten CRC AHQ will report the event to AstraZeneca/MedImmune Patient Safety or designee within 24 hours using the designated Safety e-mailbox. The designated AstraZeneca representative will work with Big Ten CRC AHQ and the Sponsor Investigator to ensure that all relevant information is provided to the AstraZeneca Patient Safety data entry site within 1 to 5 calendar days for SAEs and within 30 days for all other pregnancies.

Male subjects should refrain from fathering a child or donating sperm during the study and for 3 months following the last dose.

Any pregnancy in the partner of a male study subject from the date of the first dose until 90 days after the last dose should be reported ***within 1 business day of knowledge of the event*** to Big Ten CRC AHQ on the Pregnancy Report form (See Documents/Info tab of the EDC). The site investigator will endeavor to collect follow-up information on such pregnancies, provided the partner of the study subject provides consent.

11.2.2.3 Big Ten CRC AHQ Requirements for Reporting SAEs to AstraZeneca

Big Ten CRC AHQ will report all serious, related, and unexpected SAEs, AESIs, and other reportable events to AstraZeneca within **1 business day** of knowledge of the event. Follow-up information will be provided to AstraZeneca as reasonably requested.

Serious adverse events that do not require expedited reporting to the FDA still need to be reported to AstraZeneca, preferably using the MedDRA coding language for serious adverse events. This information should be reported monthly, and under no circumstance less frequently than quarterly.

Send SAE report and accompanying cover page by way of email to AstraZeneca's designated mailbox: AEMailboxClinicalTrialTCS@astrazeneca.com.

11.2.2.4 Big Ten CRC AHQ Requirements for Reporting SAEs to Astex

Big Ten CRC AHQ will report all SAEs to Astex within **24 hours** of receipt of the SAE Reporting Form. Follow-up information will be provided to Astex as reasonably requested.

Attention: Astex Drug Safety

North America Local Fax: 925-551 -3226

North America Toll-Free Fax: 800-576-6568

Questions regarding SAEs should be addressed to: Drugsafety@astx.com

11.2.2.5 Sponsor-Investigator Responsibilities

Big Ten CRC AHQ will send a SAE summary to the sponsor-investigator and the Michigan Institute for Clinical and Health Research (MICHR) IND/IDE Investigator Assistance Program (MIAP) **within 1 business day** of receipt of SAE Submission Form from a site. The sponsor-investigator will promptly review the SAE summary and assess for expectedness and relatedness.

11.2.2.6 Michigan Institute for Clinical and Health Research (MICHR) IND/IDE Investigator Assistance Program (MIAP) Responsibilities for Reporting SAEs to FDA

Big Ten CRC AHQ was initially designated to manage the Investigational New Drug Application (IND) associated with this protocol on behalf of the sponsor-investigator. Big Ten CRC AHQ will cross-reference this submission to AstraZeneca and Astex's parent INDs at the time of submission. Additionally, Big Ten CRC AHQ will submit a copy of these documents to AstraZeneca and Astex at the time of submission to FDA.

As of the 10APR2018 version of the protocol, MICHR-MIAP is responsible for managing the IND associated with this protocol on behalf of the sponsor-investigator. MICHR-MIAP will be responsible for all communication with the FDA in accordance with 21CFR312 which includes but is not limited to the 7 and 15 Day Reports, as well as an Annual Progress Report. MICHR-MIAP will provide Big Ten CRC AHQ with copies of any FDA communication. Big Ten CRC AHQ will provide a copy of these reports to AstraZeneca and Astex as required per contract.

11.2.2.7 IND Safety Reports Unrelated to this Trial

AstraZeneca and Astex will provide Big Ten CRC AHQ with IND safety reports from external studies that involve the study drug(s) per their guidelines. Big Ten CRC AHQ will forward the safety reports to the sponsor-investigator who will review these reports and determine if revisions are needed to the protocol or consent. Big Ten CRC AHQ will forward these reports to participating sites **within 1 business day** of receiving the sponsor-investigator's review. Based on the sponsor-investigator's review, applicable changes will be made to the protocol and informed consent document (if required). Any changes made to the protocol and/or informed consent document will be submitted to the FDA by MICHR-MIAP. All IND safety reports will also be made available to sites via the EDC.

Upon receipt from Big Ten CRC AHQ, site investigators (or designees) are responsible for submitting these safety reports to their respective IRBs, as per their IRB policies.

12. STATISTICAL METHODS

12.1 Study Design

This is a single arm, multi-center (via Big Ten Cancer Research Consortium) phase Ib/II study of patients treated with durvalumab 1500 mg IV q 4 weeks in combination with guadecitabine at the recommended phase II dose subcutaneously for 5 days in patients with metastatic RCC with a clear cell component, ECOG performance status of 0-1, who have received 0 - 1 prior therapy but no prior anti-PD-1/PD-L1/CTLA4 (Cohort 1). A smaller Cohort 2 will comprise patients who have received up to 2 prior therapies including one of them necessarily an anti-PD-1/PD-L1 therapy but did not respond to the anti-PD-1/PD-L1 therapy.

We will start with a phase 1b study to estimate the safety and toxicity of the combination of durvalumab and guadecitabine.

<u>Phase 1b Portion</u>
Six patients will be treated at dose level 0 and observed for toxicity. If 2 or fewer patients experience a dose limiting toxicity (DLT) as defined in section 5.4, then the study will continue to the phase II portion using dose level 0 as the treatment dose. If 3 or more patients have a dose limiting toxicity then another 6 patients will be accrued at the lower dose (dose -1). If 2 or fewer patients have a dose limiting toxicity then we continue to phase II at this dose, otherwise the trial stops.

Dose Level 0: Patients will receive guadecitabine 60 mg/m^2 subcutaneously for 5 days starting day 1 of a 28 day cycle and durvalumab 1500mg IV on day 8.

Dose Level -1: Patients will receive guadecitabine 45 mg/m^2 subcutaneously for 5 days starting day 1 of a 28 day cycle and durvalumab 1500mg IV on day 8.

Patients from either cohort are eligible for enrollment in the phase 1b portion of the study and will be counted towards the efficacy analysis (in addition to the safety/toxicity analysis) of their respective phase II cohorts if treated at the eventual recommended phase II dose.

12.2 Endpoints

12.2.1 Definition of Primary Endpoint

Primary Endpoint

<u>Phase 1b Primary Endpoint</u>: DLT count by dose cohort. See section 5.4 for DLT definition.

<u>Phase II Primary Endpoint</u>: Objective response rate (CR + PR) by RECIST 1.1 in Cohort 1

12.2.2 Definition of Secondary Endpoints

All Secondary endpoints will be assessed in Cohort 1 (patients with no prior anti-PD-1/PD-L1/CTLA4 therapy) and separately in Cohort 2 (patients with prior anti-PD-1/ PD-L1 therapy).

1) 2 year overall survival proportion
2) Duration of Response (DoR)
3) Progression-free survival (PFS)
4) Clinical benefit rate (ORR+ stable disease for at least 6 months rate)
5) Complete response (CR) proportion
6) Objective response rate (CR + PR) as measured by the immune related response criteria (irRC)
7) Objective response rate (CR + PR) in Cohort 2 (patients with prior anti-PD-1/ PD-L1 therapy)
8) Toxicity by CTCAE ver 4 including events of special interest such as immune mediated toxicities

12.3 Sample Size and Accrual

Primary endpoint is Objective Response Rate (CR+PR = ORR) in advanced kidney cancer patients treated with durvalumab and guadecitabine in cohort 1. Assuming an ORR of 25% in patients treated with durvalumab alone, a sample size of **36 patients in cohort 1** can detect an improvement to 45% ORR in patients treated with the combination of durvalumab + guadecitabine with 80% power assuming a one-sided 4.6% type I error with an exact binomial test. Additionally, **16 patients will be enrolled on to cohort 2** (patients who did not respond to prior anti-PD-1/PD-L1 therapy) with 80% power to detect an ORR of 25% compared to an expected ORR of 5% or less with a type I error of 4.3% with an exact binomial test. **Total of 52 patients evaluable for efficacy.**

Subjects who are inevaluable for efficacy will be replaced (section 12.6). We anticipate 3 subjects inevaluable for efficacy.

Phase 1b will include 6-12 patients with 6 patients treated at the recommended phase II dose to be also included in the phase II efficacy population.

12.4 Analysis Datasets

Phase Ib Analysis Dataset: The phase Ib dataset will include all subjects who receive at least 1 dose of durvalumab and 1 dose of guadecitabine.

Phase II Primary Efficacy Analysis Dataset: All patients treated at the recommended Phase II dose and are evaluable for efficacy, patients must have received 2 cycles of protocol therapy and have their disease re-evaluated with imaging at 8 weeks.

Phase II Safety Analysis Dataset: All patients who receive at least 1 dose of durvalumab and 1 dose of guadecitabine will be included in the safety analysis.

Minimal Protocol Treatment Population: All patients treated with at least 1 dose of durvalumab and 1 dose of guadecitabine will be included in the sensitivity analysis.

12.5 Evaluable for Safety

All subjects who receive at least 1 dose of durvalumab and 1 dose of guadecitabine will be evaluable for toxicity. Toxicity will be described using the Common Terminology Criteria for Adverse Events (CTCAE) v 4. Subjects will be assessed for toxicity with the frequency delineated in the study calendar.

12.6 Evaluable for Efficacy

To be evaluable for efficacy, patients must have received 2 cycles of protocol therapy and have their disease re-evaluated with imaging at 8 weeks. Patients who do not meet these criteria will be replaced (anticipate 3 subjects inevaluable for efficacy).

Any patient in the phase Ib portion treated at the eventual phase II dose of both drugs and evaluable for efficacy will be included in the phase II efficacy analysis.

12.7 Data Analysis Plans

12.7.1 Analysis Plans for Primary Objective

Phase Ib Analysis of Primary Endpoint.
The Phase Ib primary endpoint is toxicity by CTCAE version 4 including events of special interest such as immune mediated toxicities. DLT count and proportion will be reported. Type, grade and attribution will be described by body system in the first 6 DLT evaluable patients.

Phase II Primary Endpoint Analysis:
The Phase II primary endpoint is Objective response rate (CR + PR) by RECIST 1.1 in Cohort 1. Analysis in the evaluable population will use a binomial exact test to compare the ORR proportion in patients treated with durvalumab + guadecitabine compared to an expected proportion of 25% with durvalumab alone. The proportion of patients who have disease response of CR or PR will be reported with the 90% exact binomial confidence interval. A sensitivity analysis will be done in the intent-to-treat population where patients who do not have a disease assessment after starting treatment will be considered as PD at time of removal from therapy. A similar analysis will be completed in cohort 2 comparing cohort 2 to an expected ORR proportion of 5%.

There are no planned interim analyses or stopping rules.

12.7.2 Analysis Plans for Secondary Objectives

The Phase II secondary endpoints will be evaluated separately for cohort 1 and 2 and will be based on RECIST 1.1 unless otherwise specified. Secondary endpoints include: 2-year overall survival proportion, duration of response, progression-free survival, clinical benefit rate (ORR + stable disease rate), complete response rate, objective response rate (CR + PR) by immune related response the objective response rate (CR + PR) in cohort 2, and toxicity by CTCAE v 4. Overall survival, progression-free survival and duration of response will be assessed using Kaplan-Meier estimates including the 95% confidence band separately for cohort 1 and for cohort 2. Overall survival proportions at 2 years will be reported with 95% confidence intervals from the Kaplan-Meier estimates. Complete response rate, and clinical benefit rate (CR+PR+SD), objective response rate (CR + PR) by immune related response criteria and cohort 2 objective response rate will be reported as binomial proportions and corresponding 95% binomial confidence intervals separately for cohort 1 and cohort 2.

Analysis of toxicity as measured using CTCAE v4 will be described by body system including maximum grade and attribution as proportions of all (cohort 1 and cohort 2) treated patients. Dose reductions and holds will describe tolerability of therapy.

12.7.3 Analysis Plans for Exploratory Objectives

Levels of CXCL9 and CXCL10 in tissue samples and serum, tumor infiltrating lymphocytes of archival tumor tissue, PD-L1 expression in tumor cells and tumor infiltrating lymphocytes in archival tumor tissue, change in LINE-1 demethylation with therapy, immune cells subsets in the serum and tissue, tumor mutational burden, and epigenetic changes will be described overall and correlated with the best clinical response (RECIST 1.1). Exploratory endpoints will be described overall and by best clinical response group using plots and means or medians with the corresponding variability measure.

13. TRIAL MANAGEMENT

13.1 Data and Safety Monitoring Plan (DSMP)

The Data and Safety Monitoring Committee (DSMC) of The University of Michigan Comprehensive Cancer Center (UMCCC) is responsible for monitoring the safety and data integrity of the trial.

Big Ten CRC AHQ oversight activities include:

- Review and process all adverse events requiring expedited reporting as defined in the protocol
- Provide timely reports to MICHR-MIAP (MICHRMIAP@med.umich.edu) that require expedited reporting
- Notify participating sites of adverse events requiring expedited reporting
- Provide trial accrual progress, safety information, and data summary reports to the sponsor-investigator and MICHR-MIAP
- Coordinate weekly study team meetings for the phase I portion of the trial and then monthly meetings during the phase II portion. These meetings will include each accruing site's principal investigator, clinical research specialist and/or research nurse (other members per principal investigator's discretion).
- Monthly during the phase I portion and quarterly during the phase II portion, the study team meetings will also discuss matter related to:
    - Enrollment rate relative to expectations, characteristics of participants
    - Safety of study participants (Serious Adverse Event reporting)
    - Adherence to protocol (protocol deviations)
    - Completeness, validity and integrity of study data
    - Retention of study participants
    - These meetings are to be documented by the site data manager or study coordinator using the Protocol Specific Data and Safety Monitoring Report (DSMR), signed by the site principal investigator or designated co-investigator. Big Ten CRC AHQ will assist in this process. The DSMR can be found in the Documents/ info tab of the EDC. Each site is required to submit the completed DSMR to Big Ten CRC AHQ on a monthly basis during the phase I portion of the trial and on a quarterly basis during the phase II portion of the trial together with other pertinent documents for submission to the DSMC.

13.2 University of Michigan's Data Safety Monitoring Committee

The DSMC will review the information included on the DSMRs from the first subject enrolled until the last subject has completed the study drug interventions. Documentation of DSMC reviews will be provided to sponsor-investigator and Big Ten CRC AHQ. Issues of immediate concern by the DSMC will be brought to the attention of the sponsor-investigator and other regulatory bodies as appropriate.

The sponsor-investigator will work with Big Ten CRC AHQ and MICHR-MIAP to address the DSMC's concerns.

The DSMC will provide the sponsor-investigator and Big Ten CRC AHQ evidence of its review. Big Ten CRC AHQ will distribute this information to the participating sites for submission to their respective IRB per the local IRB's policies and procedures.

13.3 Data Quality Oversight Activities

Remote validation of data will be completed on a continual basis throughout the life cycle of the study. A summary report (QC Report) of these checks together with any queries resulting from manual review of the eCRFs will be generated for each site and transmitted to the site and the site monitor. The study site personnel will make corrections.

Monitoring visits to the trial sites will be made periodically during the trial to ensure key aspects of the protocol are followed. Additional for-cause visits may occur as necessary. Source documents will be reviewed for verification of agreement with data entered into the EDC. It is important for the site investigator and their relevant personnel to be available for a sufficient amount of time during the monitoring visits or audit, if applicable. The site investigator and institution guarantee access to source documents by Big Ten CRC AHQ or its designee.

The trial site may also be subject to quality assurance audit by AstraZeneca or Astex or its designee as well as inspection by appropriate regulatory agencies.

13.4 Compliance with Trial Registration and Results Posting Requirements

Under the terms of the Food and Drug Administration Modernization Act (FDAMA) and the Food and Drug Administration Amendments Act (FDAAA), the sponsor-investigator of the trial is solely responsible for determining whether the trial and its results are subject to the requirements for submission to the Clinical Trials Data Bank, http://www.clinicaltrials.gov. All results of primary and secondary objectives must be posted to CT.gov within a year of completion. The sponsor-investigator has delegated responsibility to Big Ten CRC AHQ for registering the trial and posting the results on clinicaltrials.gov. Information posted will allow subjects to identify potentially appropriate trials for their disease conditions and pursue participation by calling a central contact number for further information on appropriate trial locations and study site contact information.

14. DATA HANDLING AND RECORD KEEPING

14.1 Data Management

Big Ten CRC AHQ will serve as the Clinical Research Organization for this trial. Data will be collected through the web-based clinical research platform compliant with Good Clinical Practices and Federal Rules and Regulations. Big Ten CRC AHQ personnel will coordinate and manage data for quality control assurance and integrity. All data will be collected and entered into the EDC by study site personnel from participating institutions.

14.2 Case Report Forms and Submission

Generally, clinical data will be electronically captured in the EDC and correlative results will be captured in the EDC or another secure database(s). If procedures on the study calendar are performed for standard of care, at minimum, that data will be captured in the source document. Select standard of care data will also be captured in the EDC, per study-specific objectives. Please see the Data and Safety Oversight Process (DSOP) guidelines for further details.

The completed dataset is housed at Big Ten CRC AHQ and is the sole property of the sponsor-investigator's institution. It should not be made available in any form to third parties, except for authorized representatives of appropriate Health/Regulatory Authorities, without written permission from the sponsor-investigator and Big Ten CRC AHQ. After the initial publication, the complete data set will be available to all Big Ten CRC institutions.

14.3 Record Retention

To enable evaluations and/or audits from Health Authorities/ Big Ten CRC AHQ, the site investigator agrees to keep records, including the identity of all subjects (sufficient information to link records; e.g., hospital records), all original signed informed consent forms, copies of all source documents, and detailed records of drug disposition. All source documents are to remain in the subject's file and retained by the site investigator in compliance with local and federal regulations. No records will be destroyed until Big Ten CRC AHQ confirms destruction is permitted.

14.4 Confidentiality

There is a slight risk of loss of confidentiality of subject information. All records identifying the subjects will be kept confidential and, to the extent permitted by the applicable laws and/or regulations, will not be made publicly available. Information collected will be maintained on secure, password protected electronic systems. Paper files that contain personal information will be kept in locked and secure locations only accessible to the study site personnel.

Subjects will be informed in writing that some organizations including the sponsor-investigator and his/her research associates, Big Ten CRC AHQ, AstraZeneca, Astex, IRB, or government agencies, like the FDA, may inspect their medical records to verify the information collected, and that all personal information made available for inspection will be handled in strictest confidence and in accordance with local data protection laws.

If the results of the study are published, the subjects' identity will remain confidential.

15. ETHICS

15.1 Institutional Review Board (IRB) Approval

The final study protocol and the final version of the informed consent form must be approved in writing by an IRB. The site investigator must submit written approval by the IRB to Big Ten CRC AHQ before he or she can enroll subjects into the study.

The site investigator is responsible for informing the IRB of any amendment to the protocol in accordance with local requirements. In addition, the IRB must approve all advertising used to recruit

subjects for the study. The protocol must be re-approved by the IRB as local regulations require. The site investigator must submit all IRB approvals Big Ten CRC AHQ.

Progress reports and notifications of adverse events will be provided to the IRB per local regulations and guidelines.

15.2 Ethical Conduct of the Study

The study will be performed in accordance with ethical principles originating from the Declaration of Helsinki. Conduct of the study will comply with ICH Good Clinical Practice, and with all applicable federal (including 21 CFR parts 56 & 50), state, or local laws.

15.3 Informed Consent Process

The site investigator will ensure the subject is given full and adequate oral and written information about the nature, purpose, possible risks and benefits of the study. Subjects must also be notified they are free to discontinue from the study at any time. The subject should be given the opportunity to ask questions and allowed time to consider the information provided.

The subject's signed and dated informed consent must be obtained before conducting any procedure specifically for the study. The site investigator must store the original, signed informed consent form. A copy of the signed informed consent form must be given to the subject.

16. REFERENCES

1. Motzer, R.J., et al., *Pazopanib versus sunitinib in metastatic renal-cell carcinoma.* N Engl J Med, 2013. **369**(8): p. 722-31.
2. Motzer, R.J., et al., *Nivolumab for Metastatic Renal Cell Carcinoma: Results of a Randomized Phase II Trial.* J Clin Oncol, 2015. **33**(13): p. 1430-7.
3. Zou, W., J.D. Wolchok, and L. Chen, *PD-L1 (B7-H1) and PD-1 pathway blockade for cancer therapy: Mechanisms, response biomarkers, and combinations.* Sci Transl Med, 2016. **8**(328): p. 328rv4.
4. Alva, A.S., et al., *Hypomethylating agents for urologic cancers.* Future Oncol, 2011. **7**(3): p. 447-63.
5. Issa, J.P., et al., *Safety and tolerability of guadecitabine (SGI-110) in patients with myelodysplastic syndrome and acute myeloid leukaemia: a multicentre, randomised, dose-escalation phase 1 study.* Lancet Oncol, 2015. **16**(9): p. 1099-110.
6. Peng, D., et al., *Epigenetic silencing of TH1-type chemokines shapes tumour immunity and immunotherapy.* Nature, 2015. **527**(7577): p. 249-53.
7. Massard, C., et al., *Safety and Efficacy of Durvalumab (MEDI4736), an Anti-Programmed Cell Death Ligand-1 Immune Checkpoint Inhibitor, in Patients With Advanced Urothelial Bladder Cancer.* J Clin Oncol, 2016. **34**(26): p. 3119-25.
8. Ng, C.M., et al., *Rationale for fixed dosing of pertuzumab in cancer patients based on population pharmacokinetic analysis.* Pharm Res, 2006. **23**(6): p. 1275-84.
9. Wang, E., et al., *Population pharmacokinetic and pharmacodynamic analysis of tremelimumab in patients with metastatic melanoma.* J Clin Pharmacol, 2014. **54**(10): p. 1108-16.
10. Zhang, S., et al., *Fixed dosing versus body size-based dosing of therapeutic peptides and proteins in adults.* J Clin Pharmacol, 2012. **52**(1): p. 18-28.
11. Narwal, R., L.K. Roskos, and G.J. Robbie, *Population pharmacokinetics of sifalimumab, an investigational anti-interferon-alpha monoclonal antibody, in systemic lupus erythematosus.* Clin Pharmacokinet, 2013. **52**(11): p. 1017-27.
12. Wang, Y., et al., *Feasibility of using limited-population-based arterial input function for pharmacokinetic modeling of osteosarcoma dynamic contrast-enhanced MRI data.* Magn Reson Med, 2008. **59**(5): p. 1183-9.
13. Topalian, S.L., et al., *Safety, activity, and immune correlates of anti-PD-1 antibody in cancer.* N Engl J Med, 2012. **366**(26): p. 2443-54.
14. Brahmer, J.R., et al., *Safety and activity of anti-PD-L1 antibody in patients with advanced cancer.* N Engl J Med, 2012. **366**(26): p. 2455-65.

17. APPENDIX I: DURVALUAMB TOXICITY MANAGEMENT GUIDELINES

The Toxicity Management Guidelines (TMGs) have been developed to assist investigators with the recognition and management of toxicities associated with use of the immune-checkpoint inhibitors durvalumab [MEDI4736] (PD-L1 inhibitor) and tremelimumab (CTLA-4 inhibitor). Given the similar underlying mechanism of toxicities observed with these two compounds, these TMGs are applicable to the management of patients receiving either drug as monotherapy or both drugs in combination. Additionally, these guidelines are applicable when either drug is used alone or both drugs are used in combination and, also, other anti-cancer drugs (i.e., antineoplastic chemotherapy, targeted agents) are administered concurrently or sequentially as part of a protocol-specific treatment regimen. The TMGs provide information for the management of immune-mediated reactions, infusion-related reactions, and non-immune-mediated reactions that may be observed with monotherapy or combination checkpoint inhibitor regimens, with specific instructions for checkpoint inhibitor-specific dose modifications (including discontinuation) and treatment interventions. Investigators are advised however to use local practice guidelines and consult local references for the management of toxicities observed with other anti-cancer treatment.

Dosing modification and toxicity management for immune-mediated, infusion-related, and non-immune–mediated reactions associated with the use of a checkpoint inhibitor or checkpoint inhibitors in this protocol – whether that is MEDI4736 alone, tremelimumab alone, or MEDI4736 + tremelimumab in combination, or MEDI4736 +/- tremelimumab in combination with other anti-cancer drugs (i.e., antineoplastic chemotherapy, targeted agents) administered concurrently or sequentially – should therefore be performed in accordance with this Annex to Protocol, which for the purposes of submission and approval of substantial updates is maintained as a standalone document. TMG updates are iterated by date and should be used in accordance with the CTCAE version specified in the clinical study protocol.

The TMG Annex to Protocol should be read in conjunction with the Clinical Study Protocol, where if applicable additional references for the management of toxicities observed with other anti-cancer treatment are included in the specific section of the Clinical Study Protocol.

**Dosing Modification and Toxicity Management Guidelines (TMGs) for Durvalumab Monotherapy, Durvalumab in Combination with other Products, or Tremelimumab Monotherapy – 17November2020**
**General Considerations Regarding Immune-Mediated Reactions**
**These guidelines are provided as a recommendation to support investigators in the management of potential immune-mediated adverse events (imAEs).**<br><br>Immune-mediated events can occur in nearly any organ or tissue, therefore, these guidelines may not include all the possible immune-mediated reactions. Investigators are advised to take into consideration the appropriate practice guidelines and other society guidelines (e.g., NCCN, ESMO) in the management of these events. Refer to the section of the table titled "Other - Immune-Mediated Reactions" for general guidance on imAEs not noted in the "Specific Immune-Mediated Reactions" section. Early identification and management of immune-mediated adverse events (imAEs) are essential to ensure safe use of the study drug. Monitor patients closely for symptoms and signs that may be clinical manifestations of underlying immune-mediated adverse events. Patients with suspected imAEs should be thoroughly evaluated to rule out any alternative etiologies (e.g., disease progression, concomitant medications, infections). In the absence of a clear alternative etiology, all such events should be managed as if they were immune-mediated. Institute medical management promptly, including specialty consultation as appropriate. In general, withhold study drug/study regimen for severe (Grade 3) imAEs. Permanently discontinue study drug/study regimen for life-threatening (Grade 4) imAEs, recurrent severe (Grade 3) imAEs that require systemic immunosuppressive treatment, or an inability to reduce corticosteroid dose to 10 mg or less of prednisone or equivalent per day within 12 weeks of initiating corticosteroids.<br><br>Based on the severity of the imAE, durvalumab should be withheld and corticosteroids administered. Upon improvement to Grade ≤ 1, corticosteroid should be tapered over ≥ 28 days. More potent immunosuppressive agents such as TNF inhibitors (e.g., infliximab) should be considered for events not responding to systemic steroids. Alternative immunosuppressive agents not listed in this guideline may be considered at the discretion of the investigator based on clinical practice and relevant guidelines. With long-term steroid and other immunosuppressive use, consider need for *Pneumocystis jirovecii* pneumonia (PJP, formerly known as *Pneumocystis carinii* pneumonia) prophylaxis, gastrointestinal protection, and glucose monitoring.<br><br>Dose modifications of study drug/study regimen should be based on severity of treatment-emergent toxicities graded per NCI CTCAE version in the applicable study protocol.
AE Adverse event; CTC Common Toxicity Criteria; CTCAE Common Terminology Criteria for Adverse Events; imAE immune-mediated adverse event; NCI National Cancer Institute; NCCN National Comprehensive Cancer Network; ESMO European Society for Medical Oncology

Specific Immune-Mediated Reactions			
**Adverse Events**	**Severity Grade of the Event**	**Dose Modifications**	**Toxicity Management**
**Pneumonitis/Interstitial Lung Disease (ILD)**	**Any Grade**<br>(Refer to NCI CTCAE applicable version in study protocol for defining the CTC grade/severity)	**General Guidance**	**For Any Grade**<br>− Monitor patients for signs and symptoms of pneumonitis or ILD (new onset or worsening shortness of breath or cough). Evaluate patients with imaging and pulmonary function tests, including other diagnostic procedures as described below.<br>− Suspected pneumonitis should be confirmed with radiographic imaging and other infectious and disease-related aetiologies excluded, and managed as described below.<br>− Initial work-up may include clinical evaluation, monitoring of oxygenation via pulse oximetry (resting and exertion), laboratory work-up, and high- resolution CT scan.<br>− Consider Pulmonary and Infectious Diseases consults.
	**Grade 1**	• No dose modifications required. However, consider holding study drug/study regimen dose as clinically appropriate and during diagnostic work-up for other etiologies.	**For Grade 1**<br>− Monitor and closely follow up in 2 to 4 days for clinical symptoms, pulse oximetry (resting and exertion), and laboratory work-up, and then as clinically indicated.
	**Grade 2**	• Hold study drug/study regimen dose until Grade 2 resolution to Grade ≤1.<br>• If toxicity worsens, then treat as Grade 3 or Grade 4.<br>• If toxicity improves to Grade ≤1, then the decision to reinitiate study drug/study regimen will be based upon treating physician's clinical	**For Grade 2**<br>− Monitor symptoms daily and consider hospitalization.<br>− Promptly start systemic steroids (e.g., prednisone 1 to 2 mg/kg/day PO or IV equivalent).<br>− Reimage as clinically indicated, consider chest CT with contrast and repeat in 3-4 weeks.<br>− If no improvement within 2 to 3 days, additional workup should be considered and prompt treatment with IV methylprednisolone 2 to 4 mg/kg/day started.

		judgment and after completion of steroid taper.	− If no improvement within 2 to 3 days despite IV methylprednisolone at 2 to 4 mg/kg/day, promptly start immunosuppressive therapy such as TNF inhibitors (e.g., infliximab at 5 mg/kg IV once, may be repeated at 2 and 6 weeks after initial dose at the discretion of the treating provider). Caution: It is important to rule out sepsis and refer to infliximab label for general guidance before using infliximab. Consider, as necessary, discussing with study physician.
	**Grade 3 or 4**	• Permanently discontinue study drug/study regimen.	**For Grade 3 or 4**<br>− Promptly initiate empiric IV methylprednisolone 1 to 4 mg/kg/day or equivalent.<br>− Obtain Pulmonary and Infectious Diseases Consults; consider discussing with study physician, as needed.<br>− Hospitalize the patient.<br>− Supportive care (e.g., oxygen).<br>− If no improvement within 2 to 3 days, additional workup should be considered and prompt treatment with additional immunosuppressive therapy such as TNF inhibitors (e.g., infliximab at 5 mg/kg IV, may be repeated at 2 and 6 weeks after initial dose at the discretion of the treating provider). Caution: rule out sepsis and refer to infliximab label for general guidance before using infliximab.
**Diarrhea/Colitis**	**Any Grade**<br>(Refer to NCI CTCAE applicable version in study protocol for defining the CTC grade/severity)	**General Guidance**	**For Any Grade**<br>− Monitor for symptoms that may be related to diarrhea/enterocolitis (abdominal pain, cramping, or changes in bowel habits such as increased frequency over baseline or blood in stool) or related to bowel perforation (such as sepsis, peritoneal signs, and ileus).<br>− **WHEN SYMPTOMS OR EVALUATION INDICATE AN INTESTINAL PERFORATION IS SUSPECTED, CONSULT A SURGEON EXPERIENCED IN**

			**ABDOMINAL SURGERY IMMEDIATELY WITHOUT ANY DELAY.**<br>− **PERMANENTLY DISCONTINUE STUDY DRUG FOR ANY GRADE OF INTESTINAL PERFORATION.**<br>− Patients should be thoroughly evaluated to rule out any alternative etiology (e.g., disease progression, other medications, or infections), including testing for *Clostridium difficile* toxin, etc.<br>− Steroids should be considered in the absence of clear alternative etiology, even for low-grade events, in order to prevent potential progression to higher grade events, including intestinal perforation.<br>− Use analgesics carefully; they can mask symptoms of perforation and peritonitis.
	**Grade 1**	• No dose modifications.	**For Grade 1**<br>− Monitor closely for worsening symptoms.<br>− Consider symptomatic treatment, including hydration, electrolyte replacement, dietary changes (e.g., American Dietetic Association colitis diet), loperamide, and other supportive care measures.<br>− If symptoms persist,consider checking lactoferrin; if positive, treat as Grade 2 below. If negative and no infection, continue Grade 1 management.
	**Grade 2**	• Hold study drug/study regimen until resolution to Grade ≤1<br>• If toxicity worsens, then treat as Grade 3 or Grade 4.<br>• If toxicity improves to Grade ≤1, then study drug/study regimen can be resumed after completion of steroid taper.	**For Grade 2**<br>− Consider symptomatic treatment, including hydration, electrolyte replacement, dietary changes (e.g., American Dietetic Association colitis diet), and loperamide and/or budesonide.<br>− Promptly start prednisone 1 to 2 mg/kg/day PO or IV equivalent.<br>− If event is not responsive within 2 to 3 days or worsens despite prednisone at 1 to 2 mg/kg/day PO or IV equivalent, consult a GI specialist for

			consideration of further workup, such as imaging and/or colonoscopy, to confirm colitis and rule out perforation.<br>− If still no improvement within 2 to 3 days despite 1 to 2 mg/kg IV methylprednisolone, promptly start immunosuppressants such as infliximab at 5 mg/kg IV, may be repeated at 2 and 6 weeks after initial dose at the discretion of the treating provider. [a] **Caution:** it is important to rule out bowel perforation and refer to infliximab label for general guidance before using infliximab.<br>− Consider, as necessary, discussing with study physician if no resolution to Grade ≤1 in 3 to 4 days.
	**Grade 3 or 4**	**Grade 3**<br>• For patient treated with PDL-1 inhibitors, hold study drug/study regimen until resolution to Grade ≤1; study drug/study regimen can be resumed after completion of steroid taper. Permanently discontinue study drug/study regimen for Grade 3 if toxicity does not improve to Grade ≤1 within 14 days.<br>• Permanently discontinue study drug for 1) Grade 3 colitis in patients treated with CTLA-4 inhibitors or 2) Any grade of intestinal perforation in any patient treated with ICI.<br><br>**Grade 4**<br>• Permanently discontinue study drug/study regimen.	**For Grade 3 or 4**<br>− Promptly initiate empiric IV methylprednisolone 1 to 2 mg/kg/day or equivalent.<br>− Monitor stool frequency and volume and maintain hydration.<br>− Urgent GI consult and imaging and/or colonoscopy as appropriate.<br>− If still no improvement within 2 days, continue steroids and promptly add further immunosuppressants (e.g., infliximab at 5 mg/kg IV, may be repeated at 2 and 6 weeks after initial dose at the discretion of the treating provider). **Caution**: Ensure GI consult to rule out bowel perforation and refer to infliximab label for general guidance before using infliximab.<br>− **If perforation is suspected, consult a surgeon experienced in abdominal surgery immediately without any delay.**
	**Any Grade**	**General Guidance**	**For Any Grade**

**Hepatitis (elevated LFTs)**<br>Infliximab should not be used for management of immune-related hepatitis.<br><br>**PLEASE SEE shaded area immediately below this section to find guidance for management of "Hepatitis (elevated LFTS)" in HCC patients**	(Refer to NCI CTCAE applicable version in study protocol for defining the CTC grade/severity)		– Monitor and evaluate liver function test: AST, ALT, ALP, and TB.<br>– Evaluate for alternative etiologies (e.g., viral hepatitis, disease progression, concomitant medications).
	**Grade 1**	• No dose modifications.<br>• If it worsens, then treat as Grade 2.	**For Grade 1**<br>– Continue LFT monitoring per protocol.
	**Grade 2**	• Hold study drug/study regimen dose until Grade 2 resolution to Grade ≤1.<br>• If toxicity worsens, then treat as Grade 3 or Grade 4.<br>• If toxicity improves to Grade ≤1 or baseline, resume study drug/study regimen after completion of steroid taper.<br>• Permanently discontinue study drug/study regimen for any case meeting Hy's law criteria (AST and/or ALT >3 × ULN + bilirubin >2 × ULN without initial findings of cholestasis (i.e., elevated alkaline P04) and in the absence of any alternative cause.[b]	**For Grade 2**<br>– Regular and frequent checking of LFTs (e.g., every 1 to 2 days) until LFT elevations improve or resolve.<br>– If no resolution to Grade ≤1 in 1 to 2 days, consider discussing with study physician, as needed.<br>– If event is persistent (>2 to 3 days) or worsens, promptly start prednisone 1 to 2 mg/kg/day PO or IV equivalent.
	**Grade 3 or 4**	**For Grade 3**<br>For elevations in transaminases ≤8 × ULN, or elevations in bilirubin ≤5 × ULN:<br>• Hold study drug/study regimen dose until resolution to Grade ≤1 or baseline<br>• Resume study drug/study regimen if elevations downgrade to Grade ≤1 or baseline within 14 days and	**For Grade 3 or 4**<br>– Promptly initiate empiric IV methylprednisolone at 1 to 2 mg/kg/day or equivalent.<br>– If still no improvement within 2 to 3 days despite 1 to 2 mg/kg/day methylprednisolone IV or equivalent, promptly start treatment with an immunosuppressants (e.g., mycophenolate mofetil 0.5 – 1 g every 12 hours then taper in consultation with hepatology consult). Discuss with study physician if mycophenolate is not available. **Infliximab should NOT be used.**

		after completion of steroid taper.<br>• Permanently discontinue study drug/study regimen if the elevations do not downgrade to Grade ≤1 or baseline within 14 days<br>• For elevations in transaminases >8 × ULN or elevations in bilirubin >5 × ULN, discontinue study drug/study regimen.<br><br>**For Grade 4**<br>• Permanently discontinue study drug/study regimen.	– Perform Hepatology Consult, abdominal workup, and imaging as appropriate.
**Hepatitis (elevated LFTs)**<br>Infliximab should not be used for management of immune-related hepatitis.<br><br>**THIS shaded area is guidance *only* for management of "Hepatitis (elevated LFTs)" in HCC patients**<br><br>See instructions at bottom of shaded area if transaminase rise is not isolated but (at any time) occurs in setting of either **increasing bilirubin or signs of**	**Any Elevations of AST, ALT, or TB as Described Below**	**General Guidance**	**For Any Elevations Described**<br>– Monitor and evaluate liver function test: AST, ALT, ALP, and TB.<br>– Evaluate for alternative etiologies (e.g., viral hepatitis, disease progression, concomitant medications, worsening of liver cirrhosis [e.g., portal vein thrombosis]).<br>– For HBV+ patients: evaluate quantitative HBV viral load, quantitative HBsAg, or HBeAg.<br>– For HCV+ patients: evaluate quantitative HCV viral load.<br>– Consider consulting Hepatology or Infectious Diseases specialists regarding changing or starting antiviral HBV medications if HBV viral load is >2000 IU/ml.<br>– Consider consulting Hepatology or Infectious Diseases specialists regarding changing or starting antiviral HCV medications if HCV viral load has increased by ≥2-fold.<br>– For HCV+ with HBcAb+: Evaluate for both HBV and HCV as above.
		• No dose modifications.	

**DILI/liver decompensation**	Isolated AST or ALT >ULN and ≤5.0×ULN, whether normal or elevated at baseline	• If ALT/AST elevations represents significant worsening based on investigator assessment, then treat as described for elevations in the row below.<br>• For all transaminase elevations, see instructions at bottom of shaded area if transaminase rise is not isolated but (at any time) occurs in setting of either **increasing bilirubin or signs of DILI/liver decompensation**	
	Isolated AST or ALT >5.0×ULN and ≤8.0×ULN, if normal at baseline<br><br>Isolated AST or ALT >2.0×baseline and ≤12.5×ULN, if elevated >ULN at baseline	• Hold study drug/study regimen dose until resolution to AST or ALT ≤5.0×ULN.<br>• If toxicity worsens, then treat as described for elevations in the rows below. If toxicity improves to AST or ALT ≤5.0×ULN , resume study drug/study regimen after completion of steroid taper.<br>• Permanently discontinue study drug/study regimen for any case meeting Hy's law criteria, in the absence of any alternative cause. [b]	− Regular and frequent checking of LFTs (e.g., every 1 to 3 days) until elevations of these are improving or resolved.<br>Recommend consult hepatologist; consider abdominal ultrasound, including Doppler assessment of liver perfusion.<br>− Consider, as necessary, discussing with study physician.<br>− If event is persistent (>2 to 3 days) or worsens, and investigator suspects toxicity to be an imAE, start prednisone 1 to 2 mg/kg/day PO or IV equivalent.<br>− If still no improvement within 2 to 3 days despite 1 to 2 mg/kg/day of prednisone PO or IV equivalent, consider additional workup. If still no improvement within 2 to 3 days despite 2mg/kg/day of IV methylprednisolone, consider additional abdominal workup (including liver biopsy) and imaging (i.e., liver ultrasound), and consider starting immunosuppressants (e.g.,., mycophenolate mofetil 0.5 – 1 g every 12 hours then taper in consultation with hepatology consult).[a] Discuss with study physician if

			mycophenolate mofetil is not available. **Infliximab should NOT be used.**
	Isolated AST or ALT >8.0×ULN and ≤20.0×ULN, if normal at baseline<br><br>Isolated AST or ALT >12.5×ULN and ≤20.0×ULN, if elevated >ULN at baseline	• Hold study drug/study regimen dose until resolution to AST or ALT ≤5.0×ULN Resume study drug/study regimen if elevations downgrade to AST or ALT ≤5.0×ULN within 14 days and after completion of steroid taper.<br>• Permanently discontinue study drug/study regimen if the elevations do not downgrade to AST or ALT ≤5.0×ULN within 14 days	− Regular and frequent checking of LFTs (e.g., every 1-2 days) until elevations of these are improving or resolved.<br>− Consult hepatologist (unless investigator is hepatologist); obtain abdominal ultrasound, including Doppler assessment of liver perfusion; and consider liver biopsy.<br>− Consider discussing with study physician, as needed.<br>− If investigator suspects toxicity to be immune-mediated, promptly initiate empiric IV methylprednisolone at 1 to 2 mg/kg/day or equivalent.<br>− If no improvement within 2 to 3 days despite 1 to 2 mg/kg/day methylprednisolone IV or equivalent, obtain liver biopsy (if it has not been done already) and promptly start treatment with an immunosuppressant (e.g.,., mycophenolate mofetil 0.5 – 1 g every 12 hours then taper in consultation with a hepatologist). Discuss with study physician if mycophenolate is not available. **Infliximab should NOT be used.**
	Isolated AST or ALT >20×ULN, whether normal or elevated at baseline	• Permanently discontinue study drug/study regimen.	**Same as above (except would recommend obtaining liver biopsy early)**
**If transaminase rise is not isolated but (at any time) occurs in setting of either increasing total/direct bilirubin (≥1.5×ULN, if normal at baseline; or 2×baseline, if >ULN at baseline) or signs of DILI/liver decompensation (e.g., fever, elevated INR):**<br>- Manage dosing for each level of transaminase rise as instructed for the next highest level of transaminase rise<br>- For example, manage dosing for second level of transaminase rise (i.e., AST or ALT >5.0×ULN and ≤8.0×ULN, if normal at baseline, or AST or ALT >2.0×baseline and ≤12.5×ULN, if elevated >ULN at baseline) as instructed for the third level of transaminase rise (i.e., AST or ALT >8.0×ULN and ≤20.0×ULN, if normal at baseline, or AST or ALT >12.5×ULN and ≤20.0×ULN, if elevated >ULN at baseline)<br>- For the third and fourth levels of transaminase rises, permanently discontinue study drug/study regimen			
**Nephritis or renal dysfunction**	**Any Grade**<br>(Refer to NCI CTCAE applicable version in	**General Guidance**	**For Any Grade**<br>− Consult a nephrologist.

(elevated serum creatinine)	study protocol for defining the CTC grade/severity)		− Monitor for signs and symptoms that may be related to changes in renal function (e.g., routine urinalysis, elevated serum BUN and creatinine, decreased creatinine clearance, electrolyte imbalance, decreased urine output, or proteinuria).<br>− Patients should be thoroughly evaluated to rule out any alternative etiology (e.g., disease progression, infections, recent IV contrast, medications, fluid status).<br>− Consider using steroids in the absence of a clear alternative etiology even for low-grade events (Grade 2), in order to prevent potential progression to higher grade events.
	**Grade 1**	• No dose modifications.	**For Grade 1**<br>− Monitor serum creatinine weekly and any accompanying symptoms.<br>• If creatinine returns to baseline, resume its regular monitoring per study protocol.<br>• If creatinine worsens, depending on the severity, treat as Grade 2, 3, or 4.<br>− Consider symptomatic treatment, including hydration, electrolyte replacement, and diuretics.
	**Grade 2**	Hold study drug/study regimen until resolution to Grade ≤1 or baseline.<br>• If toxicity worsens, then treat as Grade 3 or 4.<br>• If toxicity improves to Grade ≤1 or baseline, then resume study drug/study regimen after completion of steroid taper.	**For Grade 2**<br>− Consider symptomatic treatment, including hydration, electrolyte replacement, and diuretics.<br>− Carefully monitor serum creatinine every 2 to 3 days and as clinically warranted.<br>− Consult nephrologist and consider renal biopsy if clinically indicated.<br>− If event is persistent beyond 3 to 5 days or worsens, promptly start prednisone 1 to 2 mg/kg/day PO or IV equivalent.<br>− If event is not responsive within 3 to 5 days or worsens despite prednisone at 1 to 2 mg/kg/day PO or IV equivalent, consider additional workup.

			When event returns to baseline, resume study drug/study regimen and routine serum creatinine monitoring per study protocol.
	**Grade 3 or 4**	• Permanently discontinue study drug/study regimen.	**For Grade 3 or 4**<br>– Carefully monitor serum creatinine daily.<br>– Consult nephrologist and consider renal biopsy if clinically indicated.<br>– Promptly start prednisone 1 to 2 mg/kg/day PO or IV equivalent.<br>– If event is not responsive within 3 to 5 days or worsens despite prednisone at 1 to 2 mg/kg/day PO or IV equivalent, consider additional workup and prompt treatment with an immunosuppressant in consultation with a nephrologist.
**Rash or Dermatitis**<br><br>**(Including Pemphigoid)**	**Any Grade**<br>(Refer to NCI CTCAE applicable version in study protocol for definition of severity/grade depending on type of skin rash)	**General Guidance**	**For Any Grade**<br>– Monitor for signs and symptoms of dermatitis (rash and pruritus).<br>– **HOLD STUDY DRUG IF STEVENS-JOHNSON SYNDROME (SJS), TOXIC EPIDERMAL NECROLYSIS (TEN), OR OTHER SEVERE CUTANEOUS ADVERSE REACTION (SCAR) IS SUSPECTED.**<br>– **PERMANENTLY DISCONTINUE STUDY DRUG IF SJS, TEN, OR SCAR IS CONFIRMED.**
	**Grade 1**	• No dose modifications.	**For Grade 1**<br>– Consider symptomatic treatment, including oral antipruritics (e.g., diphenhydramine or hydroxyzine) and topical therapy (e.g., emolient, lotion, or institutional standard).
	**Grade 2**	• For persistent (>1 week) Grade 2 events, hold scheduled study drug/study regimen until resolution to Grade ≤1 or baseline.<br>• If toxicity worsens, then treat as Grade 3.	**For Grade 2**<br>– Obtain dermatology consult.<br>– Consider symptomatic treatment, including oral antipruritics (e.g., diphenhydramine or hydroxyzine) and topical therapy<br>– Consider moderate-strength topical steroid.

		• If toxicity improves to Grade ≤1 or baseline, then resume drug/study regimen after completion of steroid taper.	− If no improvement of rash/skin lesions occurs within 3 days or is worsening despite symptomatic treatment and/or use of moderate strength topical steroid, consider discussing with study physician, as needed, and promptly start systemic steroids such as prednisone 1 to 2 mg/kg/day PO or IV equivalent.<br>− Consider skin biopsy if the event persists for >1 week or recurs.
	**Grade 3 or 4**	**For Grade 3**<br>• Hold study drug/study regimen until resolution to Grade ≤1 or baseline.<br>• If toxicity improves to Grade ≤1 or baseline, then resume drug/study regimen after completion of steroid taper.<br>• If toxicity worsens, then treat as Grade 4.<br>**For Grade 4**<br>• Permanently discontinue study drug/study regimen.	**For Grade 3 or 4**<br>− Consult dermatology.<br>− Promptly initiate empiric IV methylprednisolone 1 to 2 mg/kg/day or equivalent.<br>− Consider hospitalization.<br>− Monitor extent of rash [Rule of Nines].<br>− Consider skin biopsy (preferably more than 1) as clinically feasible. Consider, as necessary, discussing with study physician.
**Endocrinopathy**<br>(e.g., hyperthyroidism, thyroiditis, hypothyroidism, type 1 diabetes mellitus, hypophysitis, hypopituitarism, and adrenal insufficiency)	**Any Grade**<br>(Depending on the type of endocrinopathy, refer to NCI CTCAE applicable version in study protocol for defining the CTC grade/severity)	**General Guidance**	**For Any Grade**<br>− Consider consulting an endocrinologist for endocrine events.<br>− Consider discussing with study physician, as needed.<br>− Monitor patients for signs and symptoms of endocrinopathies. Non-specific symptoms include headache, fatigue, behaviour changes, mental status changes, photophobia, visual field cuts, vertigo, abdominal pain, unusual bowel habits, polydipsia, polyuria, hypotension, and weakness.<br>− Patients should be thoroughly evaluated to rule out any alternative etiology (e.g., disease progression including brain metastases, or infections).

			− Depending on the suspected endocrinopathy, monitor and evaluate thyroid function tests: TSH, free T3 and free T4 and other relevant endocrine and related labs (e.g., blood glucose and ketone levels, HgA1c).If a patient experiences an AE that is thought to be possibly of autoimmune nature (e.g., thyroiditis, pancreatitis, hypophysitis, or diabetes insipidus), the investigator should send a blood sample for appropriate autoimmune antibody testing.<br>− Investigators should ask subjects with endocrinopathies who may require prolonged or continued hormonal replacement, to consult their primary care physicians or endocrinologists about further monitoring and treatment after completion of the study.
	**Grade 1**	• No dose modifications.	**For Grade 1**<br>− Monitor patient with appropriate endocrine function tests.<br>− For suspected hypophysitis/hypopituitarism, consider consulting an endocrinologist to guide assessment of early-morning ACTH, cortisol, TSH and free T4; also consider gonadotropins, sex hormones, and prolactin levels, as well as cosyntropin stimulation test (though it may not be useful in diagnosing early secondary adrenal insufficiency).<br>− If TSH < 0.5 × LLN, or TSH >2 × ULN, or consistently out of range in 2 subsequent measurements, include free T4 at subsequent cycles as clinically indicated and consider consultation of an endocrinologist.
	**Grade 2, 3, or 4**	• For Grade 2-4 endocrinopathies other than hypothyroidism and type 1 diabetes mellitus, consider holding study drug/study	**For Grade 2, 3, or 4**<br>− Consult endocrinologist to guide evaluation of endocrine function and, as indicated by suspected endocrinopathy and as clinically indicated, consider pituitary scan.

		regimen dose until acute symptoms resolve.<br>• Study drug/study regimen can be resumed once patient stabilizes and after completion of steroid taper.<br>• Patients with endocrinopathies who may require prolonged or continued steroid replacement (e.g., adrenal insufficiency) can be retreated with study drug/study regimen if the patient is clinically stable as per investigator or treating physician's clinical judgement.<br>• If toxicity worsens, then treat based on severity.	– For all patients with abnormal endocrine work up, except those with isolated hypothyroidism or type 1 DM, and as guided by an endocrinologist, consider short-term corticosteroids (e.g., 1 to 2 mg/kg/day methylprednisolone or IV equivalent) and prompt initiation of treatment with relevant hormone replacement (e.g., hydrocortisone, sex hormones).<br>– Isolated hypothyroidism may be treated with replacement therapy, without study drug/study regimen interruption, and without corticosteroids.<br>– Isolated type 1 diabetes mellitus (DM) may be treated with appropriate diabetic therapy, and without corticosteroids. **Only hold study drug/study regimen in setting of hyperglycemia when diagnostic workup is positive for diabetic ketoacidosis**.<br>– For patients with normal endocrine workup (laboratory assessment or MRI scans), repeat laboratory assessments/MRI as clinically indicated.
**Amylase/Lipase increased**	**Any Grade**<br>(Refer to NCI CTCAE applicable version in study protocol for defining the CTC grade/severity)	**General Guidance**	**For Any Grade**<br>– For modest asymptomatic elevations in serum amylase and lipase, corticosteroid treatment is not indicated as long as there are no other signs or symptoms of pancreatic inflammation.<br>– Assess for signs/symptoms of pancreatitis<br>– Consider appropriate diagnostic testing (e.g., abdominal CT with contrast, MRCP if clinical suspicion of pancreatitis and no radiologic evidence on CT)<br>– If isolated elevation of enzymes without evidence of pancreatitis, continue immunotherapy. Consider other causes of elevated amylase/lipase<br>– If evidence of pancreatitis, manage according to pancreatitis recommendations
	**Grade 1**	• No dose modifications.	
	**Grade 2, 3, or 4**	**For Grade 2, 3, or 4**<br>• In consultation with relevant pancreatic specialist consider continuing study drug/study regimen if no clinical/radiologic evidence of pancreatitis ± improvement in amylase/lipase.	
**Acute Pancreatitis**	**Any Grade**	**General Guidance**	**For Any Grade**

	(Refer to NCI CTCAE applicable version in study protocol for defining the CTC grade/severity)		− Consider Gastroenterology referral
	**Grade 1**	• No dose modifications.	**For Grade 1**<br>− IV hydration<br>− Manage as per amylase/lipase increased (asymptomatic)
	**Grade 2, 3, or 4**	**For Grade 2**<br>• Hold study drug/study regimen dose until resolution to Grade ≤1.<br>**For Grade 3 or 4**<br>• Permanently discontinue study drug/study regimen.	**For Grade 2, 3, or 4**<br>− Promptly start systemic steroids prednisone 1 to 2 mg/kg/day PO or IV equivalent.<br>− IV hydration
**Neurotoxicity**<br>(to include but not limited to non-infectious meningitis, non-infectious encephalitis, and autonomic neuropathy, excluding Myasthenia Gravis and Guillain-Barre)	**Any Grade**<br>(Depending on the type of neurotoxicity, refer to NCI CTCAE applicable version in study protocol for defining the CTC grade/severity)	**General Guidance**	**For Any Grade**<br>− Patients should be evaluated to rule out any alternative etiology (e.g., disease progression, infections, metabolic syndromes, or medications).<br>− Monitor patient for general symptoms (headache, nausea, vertigo, behavior change, or weakness).<br>− Consider appropriate diagnostic testing (e.g., electromyogram and nerve conduction investigations).<br>− Perform symptomatic treatment with neurological consult as appropriate.<br>− **FOR TRANSVERSE MYELITIS, PERMANENTLY DISCONTINUE FOR ANY GRADE.**
	**Grade 1**	• No dose modifications.	**For Grade 1**<br>− See "Any Grade" recommendations above.
	**Grade 2**	• For acute motor neuropathies or neurotoxicity, hold study drug/study regimen dose until resolution to Grade ≤1.	**For Grade 2**<br>− Consider, as necessary, discussing with the study physician.<br>− Obtain neurology consult.

		• For sensory neuropathy/neuropathic pain, consider holding study drug/study regimen dose until resolution to Grade ≤1.<br>• Permanently discontinue study drug/study regimen if Grade 2 imAE does not resolve to Grade ≤1 within 30 days.<br>• If toxicity worsens, then treat as Grade 3 or 4.	− Sensory neuropathy/neuropathic pain may be managed by appropriate medications (e.g., gabapentin or duloxetine).<br>− Promptly start systemic steroids prednisone 1 to 2 mg/kg/day PO or IV equivalent.<br>− If no improvement within 2 to 3 days despite 1 to 2 mg/kg/day prednisone PO or IV equivalent, consider additional workup and promptly treat with an additional immunosuppressant (e.g., IV IG or other immunosuppressant depending on the specific imAE).
	**Grade 3 or 4**	**For Grade 3 or 4**<br>• Permanently discontinue study drug/study regimen.	**For Grade 3 or 4**<br>− Consider, as necessary, discussing with study physician.<br>− Obtain neurology consult.<br>− Consider hospitalization.<br>− Promptly initiate empiric IV methylprednisolone 1 to 2 mg/kg/day or equivalent.<br>− If no improvement within 2 to 3 days despite IV corticosteroids, consider additional workup and promptly treat with an additional immunosuppressant (e.g., IV IG or other immunosuppressant depending on the specific imAE).<br>− Once stable, gradually taper steroids over ≥28 days.
**Peripheral neuromotor syndromes** (such as Guillain-Barre and myasthenia gravis)	**Any Grade** (Refer to NCI CTCAE applicable version in study protocol for defining the CTC grade/severity)	**General Guidance**	**For Any Grade**<br>− The prompt diagnosis of immune-mediated peripheral neuromotor syndromes is important, since certain patients may unpredictably experience acute decompensations that can result in substantial morbidity or in the worst case, death. Special care should be taken for certain sentinel symptoms that may predict a more severe outcome, such as prominent dysphagia, rapidly progressive weakness, and signs of respiratory insufficiency or autonomic instability.

			– Patients should be evaluated to rule out any alternative etiology (e.g., disease progression, infections, metabolic syndromes or medications). It should be noted that the diagnosis of immune-mediated peripheral neuromotor syndromes can be particularly challenging in patients with underlying cancer, due to the multiple potential confounding effects of cancer (and its treatments) throughout the neuraxis. Given the importance of prompt and accurate diagnosis, it is essential to have a low threshold to obtain a neurological consult.<br>– Neurophysiologic diagnostic testing (e.g., electromyogram and nerve conduction investigations, and "repetitive stimulation" if myasthenia is suspected) are routinely indicated upon suspicion of such conditions and may be best facilitated by means of a neurology consultation.<br>– It is important to consider that the use of steroids as the primary treatment of Guillain-Barre is not typically considered effective. Patients requiring treatment should be started with IV IG and followed by plasmapheresis if not responsive to IV IG.
	**Grade 1**	• No dose modifications.	**For Grade 1**<br>– Consider discussing with the study physician, as needed.<br>– Care should be taken to monitor patients for sentinel symptoms of a potential decompensation as described above.<br>– Consult a neurologist.
	**Grade 2**	• Hold study drug/study regimen dose until resolution to Grade ≤1.<br>• Permanently discontinue study drug/study regimen if it does not resolve to Grade	**For Grade 2**<br>– Consider discussing with the study physician, as needed.<br>– Care should be taken to monitor patients for sentinel symptoms of a potential decompensation as described above.

		≤1 within 30 days or if there are signs of respiratory insufficiency or autonomic instability.	− Consult a neurologist.<br>− Sensory neuropathy/neuropathic pain may be managed by appropriate medications (e.g., gabapentin or duloxetine).<br>*MYASTHENIA GRAVIS:*<br>o Steroids may be successfully used to treat myasthenia gravis. It is important to consider that steroid therapy (especially with high doses) may result in transient worsening of myasthenia and should typically be administered in a monitored setting under supervision of a consulting neurologist.<br>o Patients unable to tolerate steroids may be candidates for treatment with plasmapheresis or IV IG. Such decisions are best made in consultation with a neurologist, taking into account the unique needs of each patient.<br>o If myasthenia gravis-like neurotoxicity is present, consider starting AChE inhibitor therapy in addition to steroids. Such therapy, if successful, can also serve to reinforce the diagnosis.<br>o Avoid medications that can worsen myasthenia gravis.<br>*GUILLAIN-BARRE:*<br>o It is important to consider here that the use of steroids as the primary treatment of Guillain-Barre is not typically considered effective.<br>o Patients requiring treatment should be started with IV IG and followed by plasmapheresis if not responsive to IV IG.
	**Grade 3 or 4**	**For Grade 3**<br>• Hold study drug/study regimen dose until resolution to Grade ≤1.<br>• Permanently discontinue study drug/study regimen if Grade 3 imAE does not	**For Grade 3 or 4**<br>− Consider discussing with study physician, as needed.<br>− Recommend hospitalization.<br>− Monitor symptoms and consult a neurologist.<br>*MYASTHENIA GRAVIS:*

		resolve to Grade ≤1 within 30 days or if there are signs of respiratory insufficiency or autonomic instability.<br><br>**For Grade 4**<br>• Permanently discontinue study drug/study regimen.	o Steroids may be successfully used to treat myasthenia gravis. They should typically be administered in a monitored setting under supervision of a consulting neurologist.<br>o Patients unable to tolerate steroids may be candidates for treatment with plasmapheresis or IV IG.<br>o If myasthenia gravis-like neurotoxicity present, consider starting AChE inhibitor therapy in addition to steroids. Such therapy, if successful, can also serve to reinforce the diagnosis.<br>o Avoid medications that can worsen myasthenia gravis.<br>*GUILLAIN-BARRE:*<br>o It is important to consider here that the use of steroids as the primary treatment of Guillain-Barre is not typically considered effective.<br>o Patients requiring treatment should be started with IV IG and followed by plasmapheresis if not responsive to IV IG.
**Myocarditis**	**Any Grade**<br>(Refer to NCI CTCAE applicable version in study protocol for defining the CTC grade/severity)	**General Guidance**<br>• Discontinue drug permanently if biopsy-proven immune-mediated myocarditis.	**For Any Grade**<br>− The prompt diagnosis of immune-mediated myocarditis is important, particularly in patients with baseline cardiopulmonary disease and reduced cardiac function.<br>− Consider discussing with the study physician, as needed.<br>− Monitor patients for signs and symptoms of myocarditis (new onset or worsening chest pain, arrhythmia, shortness of breath, peripheral edema). As some symptoms can overlap with lung toxicities, simultaneously evaluate for and rule out pulmonary toxicity as well as other causes (e.g., pulmonary embolism, congestive heart failure, malignant pericardial effusion). Consult a cardiologist early, to promptly assess

			whether and when to complete a cardiac biopsy, including any other diagnostic procedures.<br>− Initial work-up should include clinical evaluation, BNP, cardiac enzymes, ECG, echocardiogram (ECHO), monitoring of oxygenation via pulse oximetry (resting and exertion), and additional laboratory work-up as indicated. Spiral CT or cardiac MRI can complement ECHO to assess wall motion abnormalities when needed.<br>− Patients should be thoroughly evaluated to rule out any alternative etiology (e.g., disease progression, other medications, or infections)
	**Grade 1**	• No dose modifications required unless clinical suspicion is high, in which case hold study drug/study regimen dose during diagnostic work-up for other etiologies. If study drug/study regimen is held, resume after complete resolution to Grade 0.	**For Grade 1**<br>− Monitor and closely follow up in 2 to 4 days for clinical symptoms, BNP, cardiac enzymes, ECG, ECHO, pulse oximetry (resting and exertion), and laboratory work-up as clinically indicated.<br>− Consider using steroids if clinical suspicion is high.
	**Grade 2, 3 or 4**	• If Grade 2 -- Hold study drug/study regimen dose until resolution to Grade 0. If toxicity rapidly improves to Grade 0, then the decision to reinitiate study drug/study regimen will be based upon treating physician's clinical judgment and after completion of steroid taper. If toxicity does not rapidly improve, permanently. discontinue study drug/study regimen.	**For Grade 2-4**<br>− Monitor symptoms daily, hospitalize.<br>− Promptly start IV methylprednisolone 2 to 4 mg/kg/day or equivalent after Cardiology consultation has determined whether and when to complete diagnostic procedures including a cardiac biopsy.<br>− Supportive care (e.g., oxygen).<br>− If no improvement within 2 to 3 days despite IV methylprednisolone at 2 to 4 mg/kg/day, promptly start immunosuppressive therapy such as TNF inhibitors (e.g., infliximab at 5 mg/kg IV, may be repeated at 2 and 6 weeks after initial dose at the discretion of the treating provider).<br>**Caution: It is important to rule out sepsis and**

		• If Grade 3-4, permanently discontinue study drug/study regimen.	**refer to infliximab label for general guidance before using infliximab. Infliximab is contraindicated for patients who have heart failure.**
**Myositis/ Polymyositis**	**Any Grade**<br>(Refer to NCI CTCAE applicable version in study protocol for defining the CTC grade/severity)	**General Guidance**	**For Any Grade**<br>− Monitor patients for signs and symptoms of poly/myositis. Typically, muscle weakness/pain occurs in proximal muscles including upper arms, thighs, shoulders, hips, neck and back, but rarely affects the extremities including hands and fingers; also difficulty breathing and/or trouble swallowing can occur and progress rapidly. Increased general feelings of tiredness and fatigue may occur, and there can be new-onset falling, difficulty getting up from a fall, and trouble climbing stairs, standing up from a seated position, and/or reaching up.<br>− If poly/myositis is suspected, a Neurology consultation should be obtained early, with prompt guidance on diagnostic procedures. Myocarditis may co-occur with poly/myositis; refer to guidance under Myocarditis. Given breathing complications, refer to guidance under Pneumonitis/ILD.<br>Given possibility of an existent (but previously unknown) autoimmune disorder, consider Rheumatology consultation.<br>− Consider, as necessary, discussing with the study physician.<br>− Initial work-up should include clinical evaluation, creatine kinase, aldolase, LDH, BUN/creatinine, erythrocyte sedimentation rate or C-reactive protein level, urine myoglobin, and additional laboratory work-up as indicated, including a number of possible rheumatological/antibody tests (i.e., consider whether a rheumatologist consultation is indicated and could guide need for rheumatoid

			factor, antinuclear antibody, anti-smooth muscle, antisynthetase [such as anti-Jo-1], and/or signal-recognition particle antibodies). Confirmatory testing may include electromyography, nerve conduction studies, MRI of the muscles, and/or a muscle biopsy. Consider Barium swallow for evaluation of dysphagia or dysphonia.<br>− Patients should be thoroughly evaluated to rule out any alternative etiology (e.g., disease progression, other medications, or infections).
	**Grade 1**	• No dose modifications.	**For Grade 1**<br>− Monitor and closely follow up in 2 to 4 days for clinical symptoms and initiate evaluation as clinically indicated.<br>− Consider Neurology consult.<br>− Consider, as necessary, discussing with the study physician.
	**Grade 2**	• Hold study drug/study regimen dose until resolution to Grade ≤1.<br>• Permanently discontinue study drug/study regimen if it does not resolve to Grade ≤1 within 30 days or if there are signs of respiratory insufficiency.	**For Grade 2**<br>− Monitor symptoms daily and consider hospitalization.<br>− Obtain Neurology consult, and initiate evaluation.<br>− Consider, as necessary, discussing with the study physician.<br>− If clinical course is rapidly progressive (particularly if difficulty breathing and/or trouble swallowing), promptly start IV methylprednisolone 2 to 4 mg/kg/day systemic steroids <u>along with receiving input</u> from Neurology consultant<br>− If clinical course is *not* rapidly progressive, start systemic steroids (e.g., prednisone 1 to 2 mg/kg/day PO or IV equivalent); if no improvement within 2 to 3 days, continue additional work up and start treatment with IV methylprednisolone 2 to 4 mg/kg/day<br>− If after start of IV methylprednisolone at 2 to 4 mg/kg/day there is no improvement within 2 to

			3 days, consider starting another immunosuppressive therapy such as a TNF inhibitor (e.g., infliximab at 5 mg/kg IV, may be repeated at 2 and 6 weeks after initial dose at the discretion of the treating provider). **Caution: It is important to rule out sepsis and refer to infliximab label for general guidance before using infliximab.**
	**Grade 3 or 4**	**For Grade 3**<br>• Hold study drug/study regimen dose until resolution to Grade ≤1.<br>• Permanently discontinue study drug/study regimen if Grade 3 imAE does not resolve to Grade ≤1 within 30 days or if there are signs of respiratory insufficiency.<br><br>**For Grade 4**<br>• Permanently discontinue study drug/study regimen.	**For Grade 3 or 4**<br>− Monitor symptoms closely; recommend hospitalization.<br>− Obtain Neurology consult<br>− Consider discussing with the study physician, as needed.<br>− Promptly start IV methylprednisolone 2 to 4 mg/kg/day systemic steroids along with receiving input from Neurology consultant.<br>− If after start of IV methylprednisolone at 2 to 4 mg/kg/day there is no improvement within 2 to 3 days, consider starting another immunosuppressive therapy such as a TNF inhibitor (e.g., infliximab at 5 mg/kg IV, may be repeated at 2 and 6 weeks after initial dose at the discretion of the treating provider). **Caution: It is important to rule out sepsis and refer to infliximab label for general guidance before using infliximab.**<br>− Consider whether patient may require IV IG, plasmapheresis.

[a]ASCO Educational Book 2015 "Managing Immune Checkpoint Blocking Antibody Side Effects" by Michael Postow MD.
[b]FDA Liver Guidance Document 2009 Guidance for Industry: Drug Induced Liver Injury – Premarketing Clinical Evaluation.
[c]NCCN Clinical Practice Guidelines in Oncology "Management of Immunotherapy-Related Toxicities" Version 1.2020 – December 2019

AChE Acetylcholine esterase; ADL Activities of daily living; AE Adverse event; ALP Alkaline phosphatase test; ALT Alanine aminotransferase; AST Aspartate aminotransferase; BUN Blood urea nitrogen; CT Computed tomography; CTCAE Common Terminology Criteria for Adverse Events; ILD Interstitial lung disease; imAE immune-mediated adverse event; IG Immunoglobulin; IV Intravenous; GI Gastrointestinal; LFT Liver function tests; LLN Lower limit of normal; MRI Magnetic resonance imaging; NCI National Cancer Institute; NCCN National Comprehensive Cancer Network; PJP *Pneumocystis jirovecii* pneumonia (formerly known as *Pneumocystis carinii* pneumonia); PO By mouth; T3 Triiodothyronine; T4 Thyroxine; TB Total bilirubin; TNF Tumor necrosis factor; TSH Thyroid-stimulating hormone; ULN Upper limit of normal.

Other–Immune-Mediated Reactions		
**Severity Grade of the Event** (Refer to NCI CTCAE applicable version in study protocol for defining the CTC grade/severity)	**Dose Modifications**	**Toxicity Management**
**Any Grade**	Note: It is possible that events with an inflammatory or immune mediated mechanism could occur in nearly all organs, some of them are not noted specifically in these guidelines (e.g. immune thrombocytopenia, haemolytic anaemia, uveitis, vasculitis).	– The study physician may be contacted for immune-mediated reactions not listed in the "specific immune-mediated reactions" section<br>– Thorough evaluation to rule out any alternative etiology (e.g., disease progression, concomitant medications, and infections)<br>– Consultation with relevant specialist<br>– Treat accordingly, as per institutional standard.
**Grade 1**	• No dose modifications.	Monitor as clinically indicated
**Grade 2**	• Hold study drug/study regimen until resolution to ≤Grade 1 or baseline.<br>• If toxicity worsens, then treat as Grade 3 or Grade 4.<br>• Study drug/study regimen can be resumed once event stabilizes to Grade ≤1 after completion of steroid taper.<br>• Consider whether study drug/study regimen should be permanently discontinued in Grade 2 events with high likelihood for morbidity and/or mortality when they do not rapidly improve to Grade <1 upon treatment with systemic steroids and following full taper	**For Grade 2, 3, or 4**<br>Treat accordingly, as per institutional standard, appropriate clinical practice guidelines, and other society guidelines (e.g., NCCN, ESMO)
**Grade 3**	• Hold study drug/study regimen	
**Grade 4**	• Permanently discontinue study drug/study regimen	
AE Adverse event; CTCAE Common Terminology Criteria for Adverse Events; NCI National Cancer Institute.		

Infusion-Related Reactions		
**Severity Grade of the** Event (Refer to NCI CTCAE applicable version in study protocol for defining the CTC grade/severity)	**Dose Modifications**	**Toxicity Management**
**Any Grade**	**General Guidance**	**For Any Grade**<br>– Manage per institutional standard at the discretion of investigator.<br>– Monitor patients for signs and symptoms of infusion-related reactions (e.g., fever and/or shaking chills, flushing and/or itching, alterations in heart rate and blood pressure, dyspnea or chest discomfort, or skin rashes) and anaphylaxis (e.g., generalized urticaria, angioedema, wheezing, hypotension, or tachycardia).
**Grade 1 or 2**	**For Grade 1**<br>• The infusion rate of study drug/study regimen may be decreased by 50% or temporarily interrupted until resolution of the event.<br>**For Grade 2**<br>• The infusion rate of study drug/study regimen may be decreased 50% or temporarily interrupted until resolution of the event.<br>• Subsequent infusions may be given at 50% of the initial infusion rate.	**For Grade 1 or 2**<br>– Acetaminophen and/or antihistamines may be administered per institutional standard at the discretion of the investigator.<br>– Consider premedication per institutional standard prior to subsequent doses.<br>– Steroids should not be used for routine premedication of Grade ≤2 infusion reactions.
**Grade 3 or 4**	**For Grade 3 or 4**<br>• Permanently discontinue study drug/study regimen.	**For Grade 3 or 4**<br>– Manage severe infusion-related reactions per institutional standards (e.g., IM epinephrine, followed by IV diphenhydramine and famotidine, and IV glucocorticoid).
CTCAE Common Terminology Criteria for Adverse Events; IM intramuscular; IV intravenous; NCI National Cancer Institute.		

Non-Immune-Mediated Reactions		
**Severity Grade of the Event** (Refer to NCI CTCAE applicable version in study protocol for defining the CTC grade/severity)	**Dose Modifications**	**Toxicity Management**
**Any Grade**	Note: Dose modifications are not required for AEs not deemed to be related to study treatment (i.e., events due to underlying disease) or for laboratory abnormalities not deemed to be clinically significant.	Treat accordingly, as per institutional standard.
**Grade 1**	No dose modifications.	Treat accordingly, as per institutional standard.
**Grade 2**	Hold study drug/study regimen until resolution to ≤Grade 1 or baseline.	Treat accordingly, as per institutional standard.
**Grade 3**	Hold study drug/study regimen until resolution to ≤Grade 1 or baseline.<br>For AEs that downgrade to ≤Grade 2 within 7 days or resolve to ≤Grade 1 or baseline within 14 days, resume study drug/study regimen administration. Otherwise, discontinue study drug/study regimen.	Treat accordingly, as per institutional standard.
**Grade 4**	Discontinue study drug/study regimen (Note: For Grade 4 labs, decision to discontinue should be based on accompanying clinical signs/symptoms, the Investigator's clinical judgment, and consultation with the Sponsor.).	Treat accordingly, as per institutional standard.
AE Adverse event; CTCAE Common Terminology Criteria for Adverse Events; NCI National Cancer Institute.		
