# Supplementary material for: Durvalumab and guadecitabine in advanced clear cell renal cell carcinoma: results from the phase Ib/II study BTCRC-GU16-043
Source: Nat Commun. 2024 Feb 1;15:972. doi: 10.1038/s41467-024-45216-z (PMC10834488; doi:10.1038/s41467-024-45216-z)
Supplement: Supplementary file 3 — Reporting Summary [file 41467_2024_45216_MOESM3_ESM.pdf]

nature portfolio

Corresponding author(s): Yousef Zakharia MD

Last updated by author(s): Dec 4, 2023

Reporting Summary

Nature Portfolio wishes to improve the reproducibility of the work that we publish. This form provides structure for consistency and transparency in reporting. For further information on Nature Portfolio policies, see our Editorial Policies and the Editorial Policy Checklist.

Statistics

For all statistical analyses, confirm that the following items are present in the figure legend, table legend, main text, or Methods section.

n/a	Confirmed	
[ ]	[x]	The exact sample size (*n*) for each experimental group/condition, given as a discrete number and unit of measurement
[x]	[ ]	A statement on whether measurements were taken from distinct samples or whether the same sample was measured repeatedly
[ ]	[x]	The statistical test(s) used AND whether they are one- or two-sided<br>*Only common tests should be described solely by name; describe more complex techniques in the Methods section.*
[x]	[ ]	A description of all covariates tested
[x]	[ ]	A description of any assumptions or corrections, such as tests of normality and adjustment for multiple comparisons
[ ]	[x]	A full description of the statistical parameters including central tendency (e.g. means) or other basic estimates (e.g. regression coefficient) AND variation (e.g. standard deviation) or associated estimates of uncertainty (e.g. confidence intervals)
[ ]	[x]	For null hypothesis testing, the test statistic (e.g. *F*, *t*, *r*) with confidence intervals, effect sizes, degrees of freedom and *P* value noted<br>*Give P values as exact values whenever suitable.*
[x]	[ ]	For Bayesian analysis, information on the choice of priors and Markov chain Monte Carlo settings
[x]	[ ]	For hierarchical and complex designs, identification of the appropriate level for tests and full reporting of outcomes
[ ]	[x]	Estimates of effect sizes (e.g. Cohen's *d*, Pearson's *r*), indicating how they were calculated

Our web collection on statistics for biologists contains articles on many of the points above.

Software and code

Policy information about availability of computer code

Data collection	Clinical data required By the protocol where entered into the Electric Case Report Forms (eCRF) and used a fully validated secure web-enabled Electronic Data Capture (EDC) system- OnCore, a product licensed from Advarra, formerly Forte Research Systems.<br>Automatic validation edit checks in EDC and offline listings were programmed to capture data discrepancies in the eCRFs and allowed modification and validations of the entered data. The Investigator verified and signed off the eCRFs in EDC to confirm the clinical data captured were complete and accurate. The Sponsor can attest that all data and metadata will be archived in perpetuity. The data are in the EDC (Electronic Data Capture) and TMF (Trial Master File), which are retained in perpetuity.
Data analysis	All the data analyses were computed using R version 4.02, performed according to statistical analysis plan.

For manuscripts utilizing custom algorithms or software that are central to the research but not yet described in published literature, software must be made available to editors and reviewers. We strongly encourage code deposition in a community repository (e.g. GitHub). See the Nature Portfolio guidelines for submitting code & software for further information.

Data

Policy information about availability of data

All manuscripts must include a data availability statement. This statement should provide the following information, where applicable:

- Accession codes, unique identifiers, or web links for publicly available datasets
- A description of any restrictions on data availability
- For clinical datasets or third party data, please ensure that the statement adheres to our policy

The trial protocol has been provided in the Supplementary Information. The authors declare that all data supporting the findings of this trial are available within the article and Supplementary Information.

Research involving human participants, their data, or biological material

Policy information about studies with human participants or human data. See also policy information about sex, gender (identity/presentation), and sexual orientation and race, ethnicity and racism.

Reporting on sex and gender	Both males and females enrolled to the trial.
Reporting on race, ethnicity, or other socially relevant groupings	Participants from all races, ethnicities, and other socially relevant groupings were eligible to participate.
Population characteristics	Eligible subjects were ≥ 18 years old with histologically confirmed metastatic clear cell RCC (pure or mixed) with at least one RECIST 1.1 measurable lesion. Other inclusion criteria were ECOG performance status of 0 or 1, life expectancy of ≥ 12 weeks, adequate bone marrow function including white blood cells (WBC) ≥ 3000/mm3, absolute neutrophil count (ANC) ≥ 1.5 K/ mm3, hemoglobin ≥ 9 g/dL and platelet count ≥ 100,000/ mm3), adequate renal function (calculated creatinine clearance ≥ 40 cc/min using the Cockcroft-Gault formula) and adequate hepatic function (total bilirubin ≤ 1.5 × upper limit of normal (ULN), alanine aminotransferase (ALT) and aspartate aminotransferase (AST) ≤ 2.5× ULN).
Recruitment	Eligible patients were approached during routine clinic visits and screened for eligibility, consented if inclusion criteria were met. We do not anticipate bias in recruitment of participants in the study.
Ethics oversight	The study was approved by the US Food and Drug Administration and by the local Institutional Review Boards (IRB) at the participating institutions including the University of Michigan, the University of Iowa, the University of Illinois at Chicago, Rutgers University and Penn State University. All study procedures were undertaken in accordance with the Declaration of Helsinki.

Note that full information on the approval of the study protocol must also be provided in the manuscript.

Field-specific reporting

Please select the one below that is the best fit for your research. If you are not sure, read the appropriate sections before making your selection.

☒ Life sciences ☐ Behavioural & social sciences ☐ Ecological, evolutionary & environmental sciences

For a reference copy of the document with all sections, see nature.com/documents/nr-reporting-summary-flat.pdf

Life sciences study design

All studies must disclose on these points even when the disclosure is negative.

Sample size	Assuming an ORR of 25% in patients treated with durvalumab alone, a sample size of 36 patients in cohort 1 can detect an improvement to 45% ORR in patients treated with the combination a one-sided 4.6% type I error with an exact binomial test. Additionally, 16 patients will be enrolled on to cohort 2 (patients who did not respond to prior anti-PD-1/PD-L1 therapy) with 80% power an ORR of 25% compared to an expected ORR of 5% or less with a type I error of 4.3% with an exact binomial test. Total of 52 patients evaluable for efficacy.
Data exclusions	All subjects were evaluable for safety. One subject; who began cycle 2 and did not complete due to toxicity; was deemed not evaluable for efficacy, as they did not complete any on-study scans.
Replication	There is increased interest in evaluating epigenetics in solid tumors. The results of our study are worth further evaluation in large randomized clinical trial to validate the efficacy of this regimen in metastatic clear cell renal cell carcinoma.
Randomization	This was a single arm proof of concept study that did not utilize a randomization.
Blinding	Single arm study, no randomization.

Reporting for specific materials, systems and methods

We require information from authors about some types of materials, experimental systems and methods used in many studies. Here, indicate whether each material, system or method listed is relevant to your study. If you are not sure if a list item applies to your research, read the appropriate section before selecting a response.

Materials & experimental systems

n/a	Involved in the study	
☐	☒	Antibodies
☒	☐	Eukaryotic cell lines
☒	☐	Palaeontology and archaeology
☒	☐	Animals and other organisms
☐	☒	Clinical data
☒	☐	Dual use research of concern
☒	☐	Plants

Methods

n/a	Involved in the study	
☒	☐	ChIP-seq
☐	☒	Flow cytometry
☒	☐	MRI-based neuroimaging

Antibodies

Antibodies used	All antibodies used for staining are listed in Supplementary Table 3.
Validation	PBMCs were isolated by density centrifugation using Lymphoprep (StemCell Technology) according to the manufacturer's protocol. Surface staining was performed for 30min at 4 °C in MACS buffer (buffer (PBS, 2% FCS, 1 mM EDTA). For analysis of the expression of transcription factors and cytokines, surface-stained cells were fixed and permeabilized with the Perm/Fix Buffer Set (ThermoFisher) according to the manufacturer's instructions. For intracellular cytokine staining, PBMC were ex vivo restimulated with a leucocyte stimulation cocktail containing ionomycin and PMA (Sigma) for 4h in the presence of brefeldin A and monomycin (BD Biosystem). Cells were acquired by Fortessa (BD Biosciences). Data were analyzed using DIVA software (BD Biosciences).

Clinical data

Policy information about clinical studies

All manuscripts should comply with the ICMJEguidelines for publication of clinical research and a completedCONSORT checklist must be included with all submissions.

Clinical trial registration	The study NCT03308396 was approved by the US FDA
Study protocol	The trial protocol is made available as part of the supplemental material
Data collection	All data was collected by the BIG TEN Cancer Consortium and participating academic institutions.<br>Recruitment: 57 patients were recruited between 01/18/2018 and 09/24/2020.<br>Data collection was performed from January 2018 to October 2022.
Outcomes	Phase Ib<br>Primary Objective<br>1. To estimate the safety and toxicities of durvalumab in combination with guadecitabine in metastatic clear cell renal cell carcinoma<br>Phase II<br>Primary Objective:<br>1. To assess the efficacy (primary endpoint: Objective Response Rate (CR + PR) as measured by RECIST 1.1) of durvalumab plus guadecitabine in patients with advanced RCC in Cohort 1 (patients with no prior anti-PD-1/PD-L1/CTLA4 therapy).<br>Secondary Objectives:<br>1. To assess the efficacy of durvalumab plus guadecitabine in patients with advanced RCC. Secondary endpoints include: the 2-year overall survival proportion, duration of response (DoR), 12-month progression-free survival (PFS), clinical benefit rate (proportion of patients with a best response of partial/complete response/stable disease for at least 6 months), complete response (CR) rate, objective response rate (CR + PR) as measured by the immune related response criteria (irRC) and the objective response rate (CR + PR) of patients treated with durvalumab and guadecitabine in cohort 2<br>Phase II secondary endpoints will be evaluated separately for cohort 1 and 2 and will be based on RECIST 1.1 unless otherwise specified.<br>2. To evaluate the safety of guadecitabine in combination with durvalumab (secondary endpoint: toxicity by CTCAE ver 4 including events of special interest such as immune mediated toxicities) in advanced RCC (Cohorts 1 and 2).<br>Exploratory Objectives:

1. To correlate changes in CXCL9 and CXCL10 in tissue and serum as well as and LINE-1 methylation in blood with clinical response to the combination therapy
2. To correlate CD3+/CD8+ tumor infiltrating lymphocytes (TILs) and PD-L1 expression in baseline tumor tissue/TILs as well as immune cell subsets in the serum with clinical response to the combination therapy
3. To correlate tumor mutational burden and epigenetic changes with response to the combination therapy.
4. Analyze and correlate changes in methylation status and correlate tumor mutation profile in fresh tissue with response to the combination therapy

Plants

Seed stocks: N/A

Novel plant genotypes: N/A

Authentication: N/A

Flow Cytometry

Plots

Confirm that:

- [x] The axis labels state the marker and fluorochrome used (e.g. CD4-FITC).
- [x] The axis scales are clearly visible. Include numbers along axes only for bottom left plot of group (a 'group' is an analysis of identical markers).
- [x] All plots are contour plots with outliers or pseudocolor plots.
- [x] A numerical value for number of cells or percentage (with statistics) is provided.

Methodology

Sample preparation: PBMCs were isolated by density centrifugation using Lymphoprep (StemCell Technology) according to the manufacturer's protocol. Surface staining was performed for 30 min at 4 °C in MACS buffer (buffer (PBS, 2% FCS, 1 mM EDTA). For analysis of the expression of transcription factors and cytokines, surface-stained cells were fixed and permeabilized with the Perm/Fix Buffer Set (ThermoFisher) according to the manufacturer's instructions. For intracellular cytokine staining, PBMC were ex vivo restimulated with a leucocyte stimulation cocktail containing ionomycin and PMA (Sigma) for 4 h in the presence of brefeldin A and monomycin (BD Biosystem). Cells were acquired by Fortessa (BD Biosciences). Data were analyzed using DIVA software (BD Biosciences). All antibodies used for staining are listed in Supplementary Table 1.

Instrument: Lymphoprep (StemCell Technology)

Software: *Describe the software used to collect and analyze the flow cytometry data. For custom code that has been deposited into a community repository, provide accession details.*

Cell population abundance:

Target Fluorchrome Clone Cat,number Company
CD4 Pacyfic Blue RM4-5 MCD0428 ThermoFisher
CD3 PE-CF594 UCHT1 562280 BD Biosciences
CD3 PE-Cyanine7 UCHT1 25-0038-42 ThermoFisher
CD3 Pacyfic Orange UCHT1 CD0330 ThermoFisher
CD7 APC-eFluor® 780 124-1D1 47-0079-42 ThermoFisher
CD8 Alexa Fluor 700 RPA-T8 557945 BD Biosciences
CD8 PerCP-Cyanine5.5 RPA-T8 560662 BD Biosciences
CD11c APC B-ly6 559877 BD Biosciences
CD14 FITC M5E2 555397 BD Biosciences
CD15 eFluor 450 MMA 48-0158-42 ThermoFisher
CD19 PE-Cyanine7 SJ25C1 50-154-71 ThermoFisher
CD33 Alexa Fluor 700 WM-53 56-0338-42 ThermoFisher
CD45 Pacyfic Orange HI30 MHCD4530 ThermoFisher

CD274 PE MIH1 557924 BD Biosciences
CD279 PE MIH4 558694 BD Biosciences
HLA-DR PerCP-eFluor 710 L243 46-9952-42 ThermoFisher
FoxP3 eFluor 450 236A/E7 48-4777-42 ThermoFisher
GATA3 PE-CF594 L50-823 563510 BD Biosciences
RORyt PE Q21-559 563081 BD Biosciences
T-bet PerCP-Cyanine5.5 4B10 45-5825-82 ThermoFisher
Ki-67 Alexa Fluor 488 SolA15 53-5698-82 ThermoFisher
Gran. B PE-CF594 GB11 562462 BD Biosciences
IL-2 PE MQ1-17H12 554566 BD Biosciences
IL-17 Alexa Fluor 700 N49-653 560613 BD Biosciences
IL-22 APC 4F1 generated in lab
IFNy PE-Cyanine7 B27 557643 BD Biosciences
TNFa FITC MAb11 552889 BD Biosciences

Gating strategy

Describe the gating strategy used for all relevant experiments, specifying the preliminary FSC/SSC gates of the starting cell population, indicating where boundaries between "positive" and "negative" staining cell populations are defined.

☒ Tick this box to confirm that a figure exemplifying the gating strategy is provided in the Supplementary Information.
